# The Essential Oil of *Artemisia argyi* H.Lév. and Vaniot Attenuates NLRP3 Inflammasome Activation in THP-1 Cells

**DOI:** 10.3389/fphar.2021.712907

**Published:** 2021-09-16

**Authors:** Pengxiao Chen, Qi Bai, Yanting Wu, Qiongzhen Zeng, Xiaowei Song, Yuying Guo, Pengjun Zhou, Yao Wang, Xiaofeng Liao, Qiaoli Wang, Zhe Ren, Yifei Wang

**Affiliations:** ^1^ Guangzhou Jinan Biomedicine Research and Development Center, Institute of Biomedicine, College of Life Science and Technology, Jinan University, Guangzhou, China; ^2^ Key Laboratory of Bioengineering Medicine of Guangdong Province, Guangzhou, China; ^3^ Biology Postdoctoral Research Station, Jinan University, Guangzhou, China; ^4^ The First Affiliated Hospital of Jinan University, Guangzhou Overseas Chinese Hospital, Guangzhou, China; ^5^ Guangdong Provincial Key Laboratory of Large Animal Models for Biomedicine, School of Biotechnology and Health Sciences, Wuyi University, Jiangmen, China

**Keywords:** *Artemisia argyi* H. Lév. & Vaniot, essential oil, NLRP3 inflammasome, NF- kappa B, ASC oligomerization

## Abstract

*Artemisia argyi* H. Lév. and Vaniot is a traditional medical herb that has been used for a long time in China and other Asian counties. Essential oil is the main active fraction of *Artemisia argyi* H. Lév. and Vaniot, and its anti-inflammatory potential has been observed *in vitro* and *in vivo*. Here, we found that the essential oil of *Artemisia argyi* H. Lév. and Vaniot (EOAA) inhibited monosodium urate (MSU)- and nigericin-induced NLRP3 inflammasome activation. EOAA suppressed caspase-1 and IL-1β processing and pyroptosis. NF-κB p65 phosphorylation and translocation were also inhibited. In addition, EOAA suppressed nigericin-induced NLRP3 inflammasome activation without blocking ASC oligomerization, suggesting that it may inhibit NLRP3 inflammasome activation by preventing caspase-1 processing. Our study thus indicates that EOAA inhibits NLRP3 inflammasome activation and has therapeutic potential against NLRP3-driven diseases.

## Introduction

Pattern recognition receptors (PRRs) are responsible for the detection of microbial components, endogenous danger signals and environmental irritants, leading to the activation of inflammatory cascades in immune cells that contribute to pathogen clearance and tissue repair (Takeuchi and Akira, 2010). NLRP3, a member of the Nod-like receptor family, is a vital pattern recognition receptor mediating the response to broad stimuli, including lipopolysaccharide (LPS), bacteria, virus, pore-forming toxin, adenosine triphosphate (ATP) and monosodium urate (MSU) crystals (Swanson et al., 2019). Once activated, NLRP3 self-oligomerizes and binds with ASC, which subsequently recruits caspase-1 to form a multiprotein complex called the NLRP3 inflammasome, resulting in the self-cleavage and maturation of caspase-1 (Fernandes-Alnemri et al., 2007). Activated caspase-1 further cleaves pro-IL-1β and pro-IL-18 to produce active and mature forms, which are indispensable for the secretion of IL-1β and IL-18 (Martinon et al., 2002). Gasdermin D (GSDMD) is also cleaved by caspase-1 and migrates to the plasma membrane, forming nonselective pores and inducing pyroptosis (He et al., 2015; Kayagaki et al., 2015; Shi et al., 2015). Typically, NLRP3 inflammasome activation requires two steps: priming and activation. During the priming process, NF-κB-mediated upregulation of NLRP3 and pro-IL-1β is critical for the activation of the NLRP3 inflammasome (Bauernfeind et al., 2009). To date, the NLRP3 inflammasome is the most extensively studied inflammasome. Dysfunction of the NLRP3 inflammasome is implicated in various inflammatory diseases, such as type 2 diabetes (Masters et al., 2010), neuron degenerate diseases (Halle et al., 2008; Venegas et al., 2017; Ising et al., 2019) and gout (Martinon et al., 2006). Therefore, regulating the activation of the NLRP3 inflammasome is a promising therapeutic strategy for controlling inflammation-related disorders.

In recent years, *in vitro* and *in vivo* studies have revealed that several phytochemicals possess inhibitory effects on the NLRP3 inflammasome. Oridonin, a major active ingredient of Rabdosia rubescens, directly targets NLRP3 to mediate its preventive effects in mouse models of peritonitis, gouty arthritis and type 2 diabetes (He et al., 2018). Artemisinin attenuates uric acid-induced inflammation by interfering with the interaction between NEK7 and NLRP3 (Kim et al., 2019). Traditional medicine may be potentially useful resource for exploring therapeutic agents for the treatment of NLRP3 inflammasome-related diseases.

*Artemisia argyi* H. Lév. and Vaniot is a traditional Chinese medicinal herb that has been widely used to treat inflammation, dysmenorrhea, abdominal pain, uterine haemorrhage and pruritus (Song et al., 2019). Essential oil is the main component of *Artemisia argyi* H. Lév. and Vaniot. Gas chromatography-mass spectrometry (GC-MS) revealed that the essential oil of *Artemisia argyi* H. Lév. and Vaniot (EOAA) contains eucalyptol, β-caryophyllene, terpinolene and other ingredients (Guan et al., 2019). According to previous research, EOAA suppressed the inflammatory responses in a 12-O-tetradecanoylphorbol-13-acetate (TPA)-induced mouse ear edema model and downregulated the gene expression of the inflammatory mediators iNOS and COX-2 by inhibiting JAK/STAT activation (Chen et al., 2017). In addition, *Artemisia princeps* extract has been reported to suppress NLRP3 inflammasome activation (Kwak et al., 2018). However, the effect of EOAA on the activation of the NLRP3 inflammasome remains unknown.

Here, we found that EOAA extracted by steam distillation (SDAO) and EOAA extracted from CO_2_ supercritical fluid (SFEAO) inhibited the activation of the NLRP3 inflammasome induced by MSU and nigericin. Thus, our results indicate that EOAA has therapeutic potential for NLRP3 inflammasome-related diseases.

## Materials and Methods

### Materials

RPMI 1640 medium, fetal bovine serum (FBS) and penicillin-streptomycin were purchased from Gibco (Grand Island, NY, United States). Phorbol myristate acetate, cocktails of the protease and phosphatase inhibitor, RIPA lysis buffer, PMSF, LDH Cytotoxicity Assay Kit and SDS-PAGE solution kit were obtained from Beyotime Biotech (Shanghai, China). Uric acid (U2625-25G) was from Sigma-Aldrich (St. Louis, MO, United States) which was used to prepare monosodium urate according to the previous study. Nigericin (tlrl-nig) were purchased from InvivoGen (San Diego, CA, United States). Disuccinimidyl suberate (DSS, RH65890-1G) was from BioRuler (Danbury, CT, United States). The ELISA kit for IL-1β (CHE0001) and TNF-α (CHE0019) were purchased from Beijing 4A Biotech (Beijing, China). Antibodies against NLRP3 (D4D8T), Phospho-NF-κB p65 (Ser536), NF-κB p65 (D14E12) were from Cell Signaling Technology (Boston,MA,United States). Antibody against GSDMDC1 (64-Y) was obtained from Santa cruz (Santa Cruz, CA, United States). Antibody against ASC/TMS1 was from ProteinTech (Des Plaines, IL, United States). IL-1 β antibody (AF-401-SP) was from R&D systems (Minneapolis, MN, United States). Anti-pro-Caspase-1 + p10 + p12 antibody (ab179515) was from Abcam (Cambridge, MA, United States). Donkey anti-Rabbit IgG (H+L) Secondary Antibody (Alexa Fluor 488) and Chicken anti-Rabbit IgG (H+L) Cross-Adsorbed Secondary Antibody (Alexa Fluor 594) were purchased from Life technology (Grand Island, NY, United States). Other ordinary chemicals were from Guangzhou chemical reagent factory (Guangzhou, China).

### Extraction of Essential Oils

#### Hydrodistillation

500 g crushed dry leaves of *Artemisia argyi* H. Lév. and Vaniot from Nanyang City in Henan province of China were filled in round bottom boiling flask with 5 L distilled water for 3 h. Then, the essential oil was extracted by hydrodistillation in a glass Clevenger-type apparatus for 5 h as recommended by Chinese Pharmacopoeia and collected into a container. The acquired oil was subsequently dried over anhydrous sodium sulphate and sealed in the dark under refrigeration until used.

### Subcritical Extraction

500 g crushed dry leaves of *Artemisia argyi* H. Lév. and Vaniot were filled in the supercritical extraction device. The subcritical extraction system was provided by Guangzhou Fengze Machinery Equipment Installation Co., Ltd. (Guangzhou, China). The extraction temperature was set as 55°C and the extraction pressure was 20 MPa. The flow rate of CO_2_ was constant at 25 L/h for 1 h. Resover I pressure was 7 MPa and resover II pressure was 5 MPa. After extraction, two times amount of ethanol was mixed the extract of *Artemisia argyi* H. Lév. and Vaniot and stirred overnight. Precipitated wax composition was removed through vacuum filtration. The ethanol in filtrate was removed by vacuum distillation at 30°C to obtain the volatile oil of *Artemisia argyi* H. Lév. and Vaniot.

### Gas Chromatography-Mass Spectrometry Analysis of EOAA

GC/MS analysis of the essential oil was carried out on a GC-MS system (7890A-5975C, Agilent, Little Falls, DE, United States) equipped with Agilent J&W HP-5 ms capillary column (30 m × 0.25 mm × 0.25 μm) in the electron impact mode (Ionization energy: 70 eV). The ion source temperature of mass spectrometer was 230°C. The temperature program was started at 50°C, remaining at this temperature for 2 min, and increased to 200°C with a flow rate of 5°C/min, then held for 2 min, followed by increasing to 240°C by 30°C/min, and then held for 1 min. Split injection (1 μl) was conducted with a split ratio of 1:20. Helium was used as the carrier gas with a flow rate of 1.0 ml/min. Volatile compounds were identified by comparing the obtained mass spectra of the analytes with those of authentic standards from the NIST libraries using MassHunter software. The identified components were analyzed quantitatively by area normalization.

### Cell Culture and Stimulation

THP-1 cells were obtained from the American Type Culture Collection and STR profiling authentication of THP-1 cells (Supplementary material) was conducted by Cellcook Biotech (Guangzhou, China). THP-1 cells were cultured in RPMI 1640 medium containing 10% FBS at 37°C in a humidified incubator with 5% CO2 and 95% air. The cells were routinely tested for mycoplasma contamination. Differentiated THP-1 cells were acquired by incubation with 500 nM phorbol myristate acetate (PMA) for 3 h. Before induction of inflammasome activation, the medium was replaced with Opti-MEM. Then, the cells were treated with 500 μg/ml MSU with or without essential oil for 6 h. Differentiated THP-1 cells were treated with nigericin for 1 h to induce inflammasome activation. Essential oil was added to the cell culture 1 h before nigericin induction.

### Cell Viability Assay

PMA treated THP-1 cells (1 × 10^4^ cells) were added in each well of 96-well plated and allowed the attachment of cells for 12 h. After washing with PBS, cells were exposed to series of diluted SFEAO and SEAO with 1640 medium for additional 24 h. SFEAO and SEAO were original mix with PEG400 at 10% v/v. The medium in each well was then removed followed by washing with PBS. 10ul CCK-8 in 100 ul 1640 medium was added to each well and the plates were incubated at 37°C continually. The absorptance of each well was measured at 450 nm every 30 min using microplate reader (ELX800, BioTek, Winooski, VT, United States). Until it had reached above 1.0, the data of absorptance was recorded. The percentage of viability was calculated according to the following equation. Percentage of viability = (OD of sample-OD of blank)/(OD of control-OD of blank) × 100%. Wells only contained cell culture medium was set as blank. Cells treated without SFEAO or SEAO were set as control and cell treated with SFEAO or SEAO were set as sample.

### ELISA

Cell culture supernatants were collected after separation from the cell debris through centrifugation. Then, the levels of IL-1β and TNF-α in the supernatants were assessed using ELISA kits according to the manufacturer’s guidelines. In brief, a series concentrations of standard and diluted cell supernatants were added to the primary antibody coated plate and incubated at 37°C for 90 min. Wash the plate for four times and flap the plate to dry. Add biotinylated primary antibody to each well and incubated at 37°C for 60 min. Again, wash the plate for four times and flap the plate to dry. Add enzyme conjugated-second antibody and incubate in dark at 37°C for 30 min. Wash the plate for four times and flap the plate to dry. Add chromogenic substrate and incubate in dark at 37°C for 30 min. Finally, add stop solution into wells and measure OD at 450 nm with microplate reader. Calculate the concentration of IL-1β and TNF-α in cell supernatant according to the standard curve fitted by four parameters logistic fitting method.

### Lactate Dehydrogenase Assay

The release of LDH in the culture medium was assayed with an LDH cytotoxicity kit according to the manufacturer’s guidelines. Cells were seeded on 96 wells plate. Select one group of wells as “group of maximal LDH release rate.” Before treating cells as the method in “Cell culture and stimulation” section, wash cells with PBS and change to serum-free medium. 1 h before the end of treatment, add LDH release reagent in wells of “group of maximal LDH release rate.” At the end of treatment, the plate was centrifuged at 400 g for 5 min. Transfer 120 μl supernatant in a new plate. Add INT (2-p-iodophenyl-3-nitrophenyl tetrazolium chloride) and lactic acid solution in each well and incubate the plate in dark at room temperature for 30 min. Measure OD at 490 nm and calculate the release rate of LDH as the following equation:

Release rate of LDH = (OD of treated sample-OD of control)/(OD of “group of maximal LDH release rate”-OD of control) × 100%

### Immunoblotting

Cells were lysed in RIPA buffer on ice for 30 min, and then supernatants were collected through centrifugation at 14,000 g/min for 15 min. The protein concentration of the supernatants was measured using the BCA method. After the addition of 5× loading buffer, the cell lysate was heated to 100°C. The denatured cell lysate sample was loaded on an 8% or 10% polyacrylamide gel. After transfer to the PVDF membrane and incubation in nonfat milk, the membrane was incubated overnight in the indicated antibody solution at 4°C. The signal on the membrane was detected with an enhanced chemiluminescence detection kit (4AW012-1000, Beijing 4A Biotech) and acquired via an imaging system (Tanon 4600 SF, Biotanon, Shanghai, China). Antibodies against NLRP3 (D4D8T), Phospho-NF-κB p65 (Ser536), NF-κB p65 (D14E12), GSDMDC1 (64-Y), ASC/TMS1, IL-1β and pro-Caspase-1 + p10 + p12 were used for detecting the corresponding proteins.

### ASC Oligomerization Assay

After the treatment, the cells were lysed in AO buffer (1% Triton × 100, 20 mM HEPES-KOH, pH 7.5, 150 mM KCl, and complete protease and phosphatase inhibitor cocktail) by syringing 30 times through a 21-gauge needle. The lysates were centrifuged at 3,200 g for 15 min at 4°C and separated into the soluble fraction (the supernatant) and insoluble fraction (the pellet). Equal amount of soluble fraction was collected, and 5× loading buffer was added. The insoluble part was rinsed in PBS twice and resuspended in 60 µl PBS containing 4 mM disuccinimidyl suberate (DSS). The cross-linking reaction was performed at room temperature for 30 min with rotation (Lugrin and Martinon, 2017). Then, the same volume of nonreducing loading buffer was added. All the samples were boiled for 10 min and loaded on polyacrylamide gels for immunoblotting.

### Immunofluorescence

For detection of ASC specks and NF-κB p65 translocation, cells were seeded on coverslips the day before the following procedures. After the indicated treatment, the cells were fixed with 4% paraformaldehyde, permeabilized with PBS containing 0.1% Triton × 100, and blocked with 3% BSA in PBST. The cells were stained with ASC antibody and NF-κB p65 antibody overnight at 4°C. The next day, anti-rabbit antibodies (Alexa Fluor 488 and Alexa Fluor 594) were added for incubation at room temperature for 1 h. The nuclei were counterstained with DAPI. All of the images were acquired with a fluorescence microscope (Zeiss LSM880 Airyscan, Zeiss, Germany).

### Statistical Analysis

All data are expressed as the mean±SD and are representative of at least three different experiments or biological replications. Statistical analysis was performed in GraphPad using Student’s *t*-test for two groups and one-way ANOVA for multiple groups. Differences with *p* values less than 0.05 were considered to be statistically significant.

## Results

### GC-MS Analysis of EOAA

**FIGURE 1:**
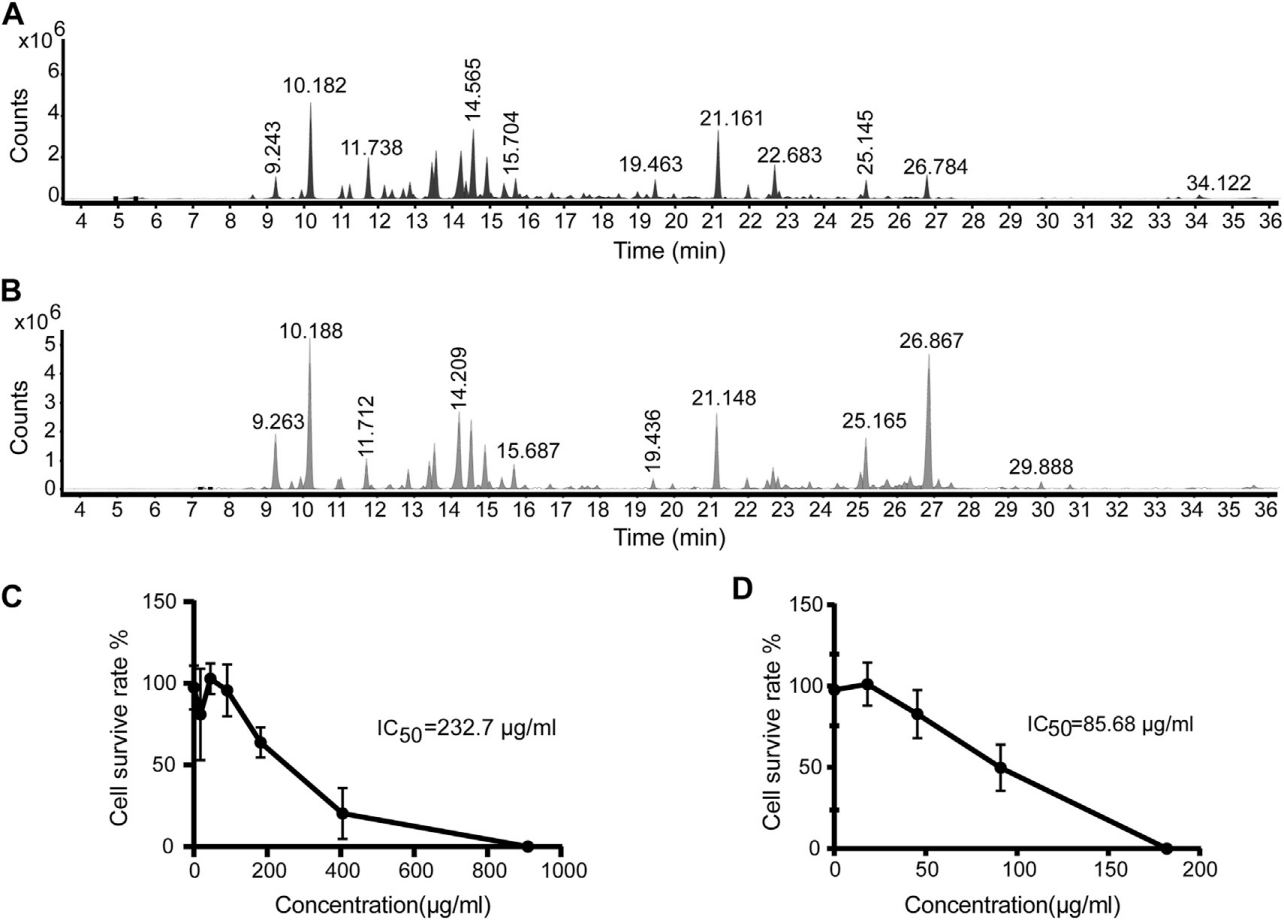
GC-MS analysis of EOAA and the effect of EOAA on cell viability. Typical total ion chromatography (TIC) of SFEAO **(A)** and SDAO **(B)**. THP-1 cells were incubated with different concentrations of SFEAO and SDAO. The effect of SFEAO **(C)** and SDAO **(D)** on cell viability were determined by CCK-8 assay. IC_50_: the half maximal inhibitory concentration. Data were from three independent experiments with biological duplicates in each (**C**,**D**; mean and SD of *n* = 6).

The *Artemisia argyi* H. Lév. and Vaniot used in this study was collected from Henan Province of China and EOAA was extracted with supercritical fluid extraction and hydrodistillation. The chemical composition of the essential oil was analyzed by GC-MS and was showed in Figures 1A,B. As listed in Tables 1, 2, a total of 29 and 26 compounds were identified for the SFEAO and SDAO, accounting for the 85.7 and 81.7% of total oil, respectively. The major components were found to be monoterpenoids, sesquiterpenoids, phenols, ketones, aldehydes in SFEAO and SDAO. Five major compounds in common were eucalyptol, terpinen-4-ol, β-caryophyllene, (+)-borneol, and α-terpineol. The most abundant composition in SFEAO is eucalyptol. Differently, neointermedeol was most abundnant in SDAO, accounting for 16.12 ± 0.95%. The cytotoxicity effects of SFEAO and SDAO were determined by CCK-8 assay. As showed in Figures 1C,D, the IC_50_ concentrations of SFEAO and SDAO were 232.7 and 85.7 μg/ml respectively, which indicated that SFEAO was less cytotoxic than SDAO.

**TABLE 1:** Chemical compositions of SFEAO.

Rt (min)	Compound	CAS	Peak area (%)
9.243	2,5,5-trimethyl-3,6-heptadien-2-ol	26127-98-0	2.33 ± 0.14
9.937	p-Cymene	99-87-6	0.91 ± 0.05
10.182	1,8-Cineole	470-82-6	8.96 ± 0.48
11.030	3,3,6-trimethylhepta-1,5-dien-4-one	546-49-6	1.08 ± 0.04
11.235	Sabinene hydrate	546-79-2	1.55 ± 0.12
11.738	3,3,6-trimethylhepta-1,5-dien-4-ol	27644-04-8	3.50 ± 0.38
12.170	trans-Sabinene hydrate	17699-16-0	1.59 ± 0.15
12.376	Thujone	546-80-5	1.02 ± 0.11
12.678	β-Thujone	471-15-8	1.95 ± 0.54
12.856	4-Isopropyl-1-Methyl-2-Cyclohexen-1-Ol	619-62-5	4.60 ± 1.40
13.449	cis-Sabinol	3310-2-9	5.24 ± 1.11
13.563	(±)-Camphor	464-49-3	0.29 ± 2.12
13.910	cis-Chrysanthenol	55722-60-6	1.26 ± 0.25
14.233	Borneol	507-70-0	6.31 ± 0.27
14.244	6-Camphenol	3570-04-5	1.93 ± 0.41
14.366	1,5-Heptadien-4-ol, 3,3,6-trimethyl-, 4-acetate, (4S)- (ACI)	3465-88-1	1.62 ± 0.08
14.565	Terpinen-4-ol	562-74-3	8.72 ± 0.36
14.931	α-Terpineol	98-55-5	5.26 ± 0.43
15.164	trans-Piperitol	16721-39-4	1.79 ± 0.23
15.388	(S)-Verbenone	1196-01-6	0.87 ± 0.16
15.704	cis-Carveol	1197-06-4	2.44 ± 0.24
18.987	γ-Terpineol	586-81-2	0.71 ± 0.08
19.463	Eugenol	97-53-0	2.07 ± 0.18
21.161	β-Caryophyllene	87-44-5	8.08 ± 0.45
21.968	Humulene	6753-98-6	1.50 ± 0.10
22.683	(±)-β-Copaene	18252-44-3	4.01 ± 0.36
22.805	(+)-β-Selinene	17066-67-0	0.82 ± 0.09
25.145	Caryophyllene oxide	1139-30-6	2.37 ± 0.28
26.784	Neointermedeol	5945-72-2	2.93 ± 0.32

**TABLE 2:** Chemical compositions of SDAO.

Rt (min)	Compound	CAS	Peak area (%)
9.263	2,5,5-trimethyl-3,6-heptadien-2-ol	26127-98-0	4.50 ± 0.13
9.699	4-Carene	29050-33-7	0.36 ± 0.17
9.941	p-Cymene	99-87-6	1.08 ± 0.13
10.068	β-Phellandrene	555-10-2	0.63 ± 0.11
10.188	1,8-Cineole	470-82-6	10.66 ± 1.38
10.947	γ-Terpinene	99-85-4	0.37 ± 0.25
11.003	3,3,6-trimethylhepta-1,5-dien-4-one	546-49-6	0.71 ± 0.06
11.712	3,3,6-trimethylhepta-1,5-dien-4-ol	27644-04-8	2.05 ± 0.12
12.838	trans-4-Isopropyl-1-methyl-2-cyclohexen-1-ol	29803-81-4	1.52 ± 0.07
13.407	cis-Sabinol	3310-2-9	2.06 ± 0.64
13.539	(±)-Camphor	464-49-3	3.39 ± 0.05
14.209	Borneol	507-70-0	7.24 ± 0.10
14.529	Terpinen-4-ol	562-74-3	5.85 ± 0.06
14.905	α-Terpineol	98-55-5	5.17 ± 2.10
15.310	trans-Piperitol	16721-39-4	0.90 ± 0.10
15.687	cis-Carveol	1197-06-4	2.00 ± 0.12
19.436	Eugenol	97-53-0	0.64 ± 0.06
21.148	β-Caryophyllene	87-44-5	5.85 ± 0.16
21.966	Humulene	6753-98-6	0.81 ± 0.04
22.666	β-Copaene	18252-44-3	1.33 ± 0.17
22.792	(+)-β-selinene	17066-67-0	0.80 ± 0.011
25.028	Espatulenol	6750-60-3	1.54 ± 0.19
25.165	Caryophyllene oxide	1139-30-6	4.87 ± 0.66
26.361	Bicyclo [7.2.0]undecan-3-ol, 11,11-dimethyl-4,8-bis(methylene)- (9CI, ACI)	79580-01-1	0.48 ± 0.34
26.867	Neointermedeol	5945-72-2	16.12 ± 0.95
27.123	trans-Longipinocarveol	889109-69-7	0.80 ± 0.11

### EOAA Inhibits the Release of Processed Caspase-1 and IL-1β Induced by MSU

**FIGURE 2:**
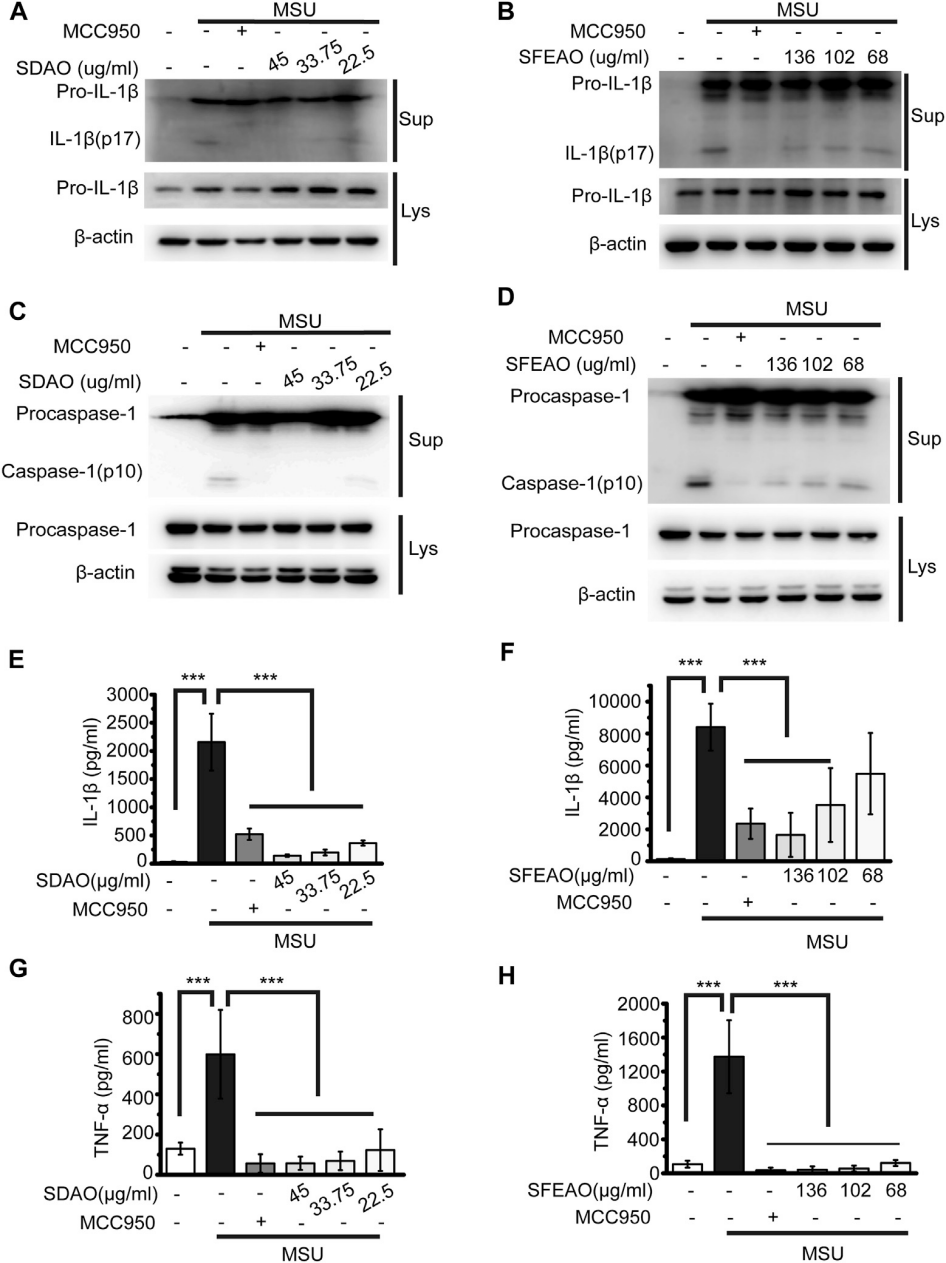
EOAA inhibits the release of processed caspase-1 and secretion of IL-1β and TNF-α induced by MSU. PMA-primed THP-1 cells were treated with or without MCC950 (20 μM) or different concentrations of SDAO or SFEAO and stimulated with MSU (500 μg/ml) for 6 h. The level of mature IL-1β **(A**,**B)** and caspase-1 **(C**,**D)** cleavage were assessed in the supernatant. The levels of pro-IL-1β and procaspase-1 were assessed in whole-cell extracts by immunoblotting. β-Actin was used as an internal control. Concentrations of IL-1β **(E**,**F)** and TNF-α **(G**,**H)** in the supernatant were determined by ELISA. Data were from three independent experiments with biological duplicates in each (**E**–**H**; mean and SD of *n* = 6) or are representative of at least three independent experiments **(A**–**D)**. Statistics were analyzed using one-way ANOVA by Bonferroni post hoc test, *p < 0.05, **p < 0.01, ***p < 0.001.

When the NLRP3 inflammasome is activated, procaspase-1 self-cleaves into activated caspase-1 (p10), which further cleaves pro-IL-1β to form IL-1β (p17). The activated fragments of both caspase-1 (p10) and IL-1β (p17) are secreted and thus can be detected in the cell culture supernatant (Guey and Petrilli, 2016). As shown in Figures 2A,B, SDAO and SFEAO at different concentrations inhibited the processing of IL-1β after MSU treatment for 6 h. Consistent with these results, the abundance of the activated fragment of caspase-1 was decreased in SDAO- and SFEAO-treated cell culture supernatants (Figures 2C,D). In addition, the inhibitory effects of SDAO and SFEAO were dose-dependent. MCC950 was used as a positive control (Coll et al., 2015), and the amounts of activated caspase-1 and IL-1β were lowest with MCC950 among the different treatments. These data indicate that SDAO and SFEAO can inhibit the activation of NLRP3 inflammasome.

**FIGURE 3:**
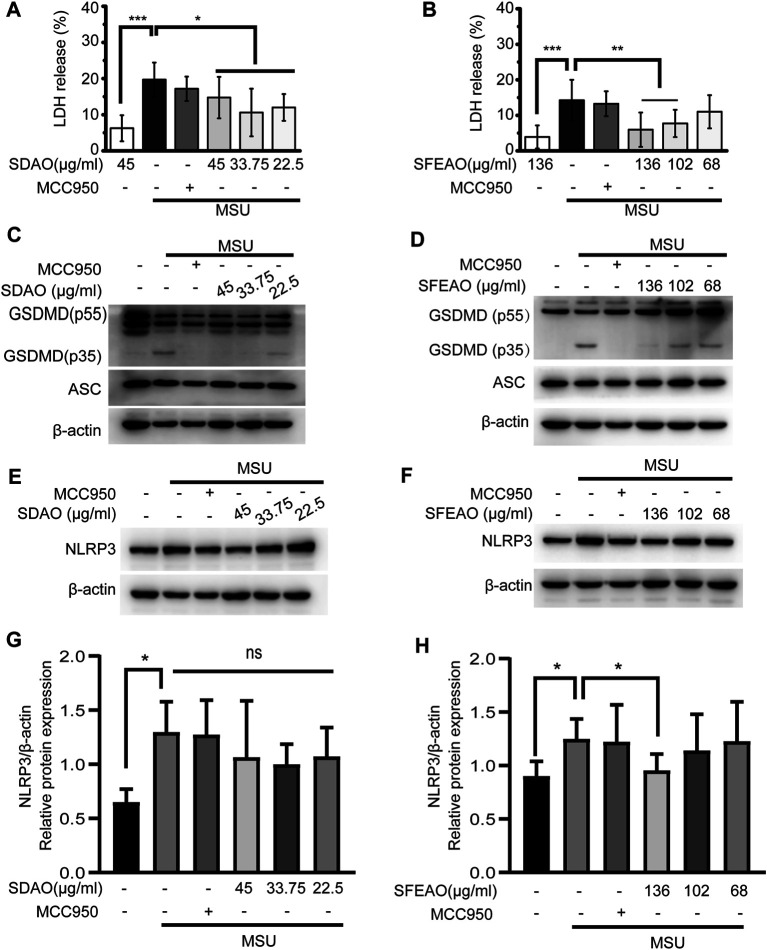
EOAA inhibits the cleavage of GSDMD, the release of LDH and the expression of NLRP3 after MSU treatment. PMA-primed THP-1 cells were treated with or without MCC950 (20 μM) or different concentrations of SDAO or SFEAO upon stimulation with MSU (500 μg/ml) for 6 h. The release of LDH in cell supernatants was measured by LDH assay **(A**,**B)**. The cleavage of GSDMD and the amount of ASC in the whole-cell extract were assessed by immunoblotting **(C**,**D)**. The protein expression levels of NLRP3 **(E**,**F)** in the cytoplasm were measured utilizing immunoblotting. β-Actin was acted as internal reference. NLRP3 expression was analyzed by densitometric quantification **(G**,**H**, *n* = 3 for **G**, *n* = 5 for **H**). Data were from three independent experiments with six biological duplicates in each (**A**,**B**; mean and SD of *n* = 18) or are representative of at least three independent experiments **(C**–**F)**. Arcsine-square root transformation of the percent data was used for statistical analysis. Statistics were analyzed using one-way ANOVA by Bonferroni post hoc test, *p < 0.05, **p < 0.01, ***p < 0.001.

The activation of the NLRP3 inflammasome causes cell membrane rupture, which allows the release of cytokines such as IL-1β and other cellular components. This process is called pyroptosis. LDH was released into the cell culture supernatant only when the integrity of the cell membrane was disrupted (Evavold et al., 2018). Thus, the concentrations of IL-1β and LDH in the supernatant can indicate the extent of cell pyroptosis after different treatments. As shown in Figures 2E,F, both SDAO and SFEAO decreased the concentration of IL-1β after MSU treatment. Moreover, the concentration of TNF-α was decreased by SDAO and SFEAO (Figures 2G,H). The release rate of LDH was also decreased by SDAO and SFEAO (Figures 3A,B). This suggests that SDAO and SFEAO inhibit pyroptosis induced by NLRP3 inflammasome activation.

The mechanism of cell pyroptosis involves the cleavage of GSDMD. Activated caspase-1 cleaves GSDMD into GSDMD-N and other fragments. This cleavage induces the oligomerization of GSDMD-N on the cell membrane, which leads to the formation of a nonselective pore and cell membrane collapse (He et al., 2015; Kayagaki et al., 2015; Shi et al., 2015). We found that SDAO and SFEAO decreased the amount of GSDMD-N (GSDMD p35) (Figures 3C,D), which indicates their inhibitory effect on pyroptosis induced by MSU.

### EOAA Inhibits the Activation of NF-κB and has Little Effect on Protein Expression of NLRP3

There are two processes involved in the activation of the NLRP3 inflammasome. The first process is priming, in which the activation of NF-κB is needed to promote the transcription of NLRP3 and IL-1β. Without the accumulation of sufficient amounts of NLRP3, the NLRP3 inflammasome cannot be activated (Bauernfeind et al., 2009). We found that NLRP3 expression increased after MSU treatment. SDAO didn’t significantly inhibited the expression of NLRP3 in MSU-treated cells (Figures 3E,G). However, SFEAO (136 μg/ml) induced a slight decrease on expression of NLRP3 in MSU-treated cells (Figures 3F,H). Additionally, MSU induced a mild increase in gene expression of NLRP3 and IL-1β (Supplementary Figure S1), which was consistent with the result of protein expressions. Notably, both SFEAO (136 μg/ml) and SDAO (45 μg/ml) inhibited the gene expression of NLRP3 and IL-1β upon MSU stimulation (Supplementary Figure S1).

**FIGURE 4:**
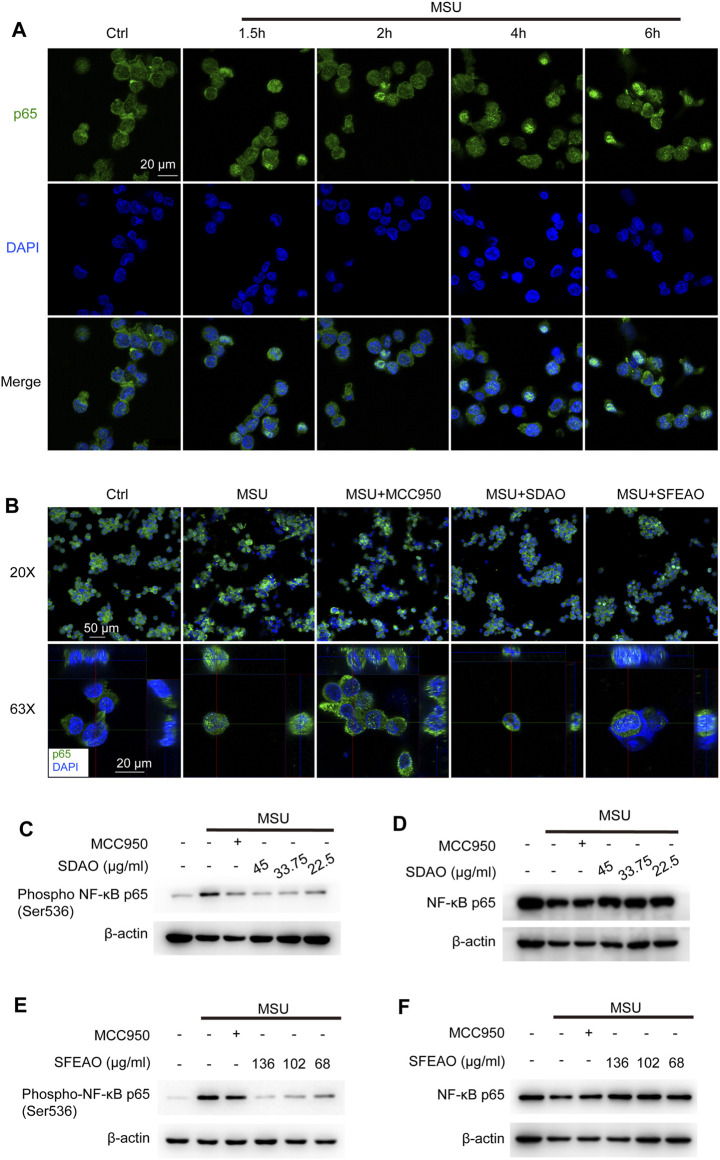
EOAA inhibits the nuclear translocation of p65 and phosphorylation of p65 after MSU stimulation. The subcellular localization of p65 was determined by indirect immunofluorescence using fluorescence microscopy after staining with a p65 antibody (green). Nuclear DNA was revealed by DAPI staining (blue). **(A)** PMA-primed THP-1 cells were stimulated with MSU (500 μg/ml) for different durations. **(B)** PMA-primed THP-1 cells were treated with or without MCC950 (20 μM), SDAO (45 μg/ml) or SFEAO (136 μg/ml) upon stimulation with MSU (500 μg/ml) for 6 h. Two magnifications of immunofluorescence pictures were acquired, and Z-stack pictures at 63X were processed using the Image software package for orthogonal view. The green and red lines in 63X images indicate the positions within the image of the projection given on the upper (XZ surface) and right (YZ surface), respectively. PMA-primed THP-1 cells were treated with or without MCC950 (20 μM) or different concentrations of SDAO or SFEAO upon stimulation with MSU (500 μg/ml) for 6 h. The protein expression levels of p-p65 **(C**,**E)** and NF-κB p65 **(D**,**F)** in the cytoplasm were measured utilizing immunoblotting. β-Actin was acted as internal reference.

NF-κB is a crucial transcription factor in inflammatory reaction and is composed of p50/p105 (NF-κB1) and p65 (RelA). In the resting state, NF-κB localizes to cells and binds to an inhibitory protein, IκBα (Hayden and Ghosh, 2004). IKKβ is the kinase to phosphorylate IκBα. Phosphorylated IκBα is ubiquitinated and translocated to the proteosome for degradation, which causes the release of p65 and exposure of nuclear-located guide peptides (Karin and Ben-Neriah, 2000). At the same time, p65 is phosphorylated by IKKβ, which is helpful for the maximum activity of NF-κB (Chen and Greene, 2004). Former study has shown that MSU induced NF-κB dependent transcriptional activation of IL-8 promotor (Liu et al., 2000). We detected the location of p65 in cells after MSU treatment for different times. The data showed that nuclear p65 began to translocate to the nucleus after MSU treatment for 2 h and was sustained in the nucleus after MSU treatment for 6 h (Figure 4A). Then, we detected the effect of SDAO and SFEAO on the translocation of p65. The results shown in Figure 4B suggested that SDAO and SFEAO inhibited the translocation of NF-κB after MSU treatment for 6 h. Further, we detected the phosphorylation of p65 at Ser536. Different concentrations of SDAO and SFEAO decreased the phosphorylation of p65 at Ser536 (Figures 4C,E). However, neither SDAO nor SFEAO changed the expression of total p65 (Figures 4D,F). Although SDAO and SFEAO inhibited the activation of NF-κB and decreased the transcriptional level of NLRP3 slightly, the protein expression of NLRP3 was not altered by SDAO and SFEAO.

### EOAA Blocks the Oligomerization of ASC

**FIGURE 5:**
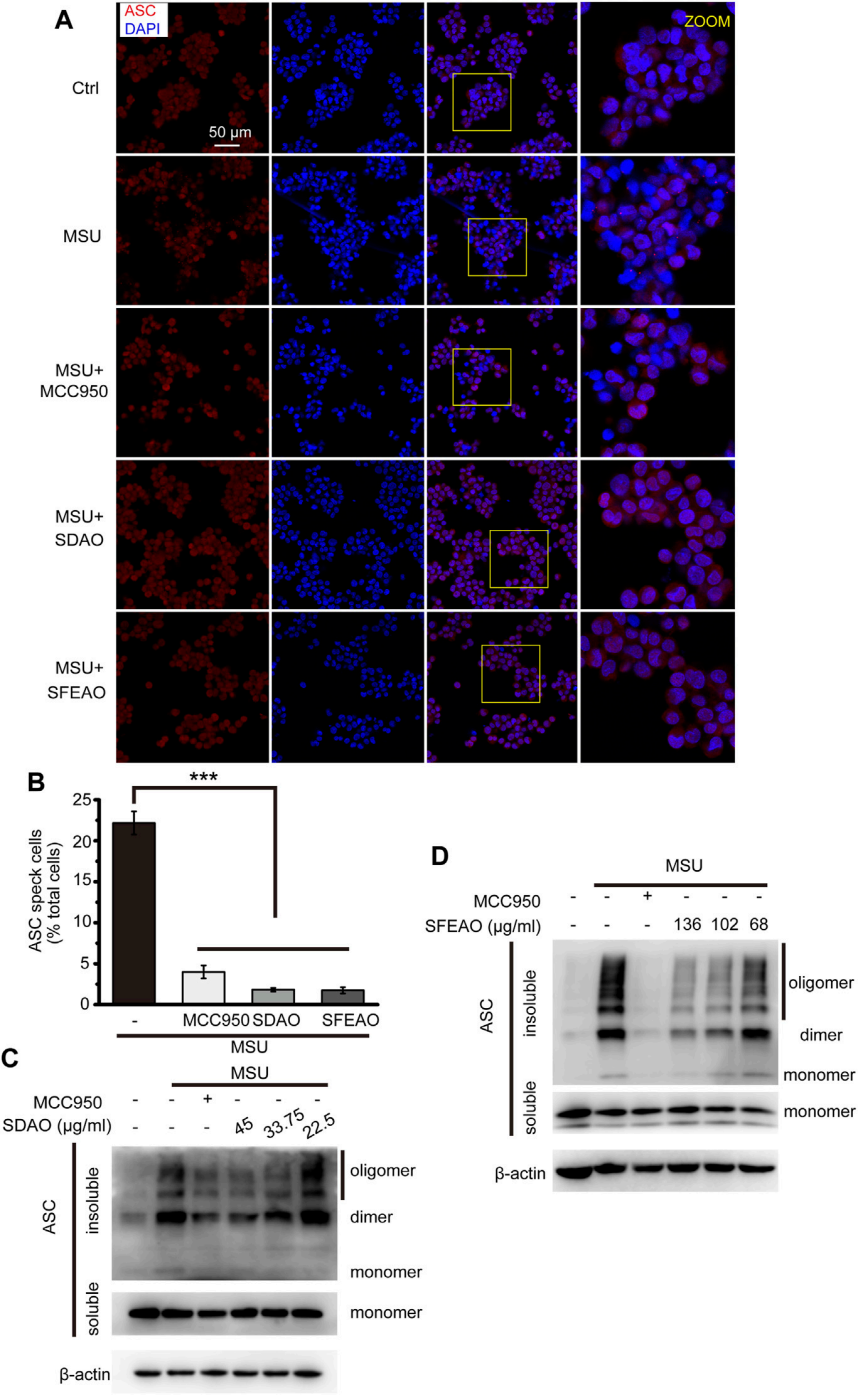
EOAA inhibits the formation of ASC oligomers induced by MSU. PMA-primed THP-1 cells were treated with or without MCC950 (20 μM) or different concentrations of SDAO or SFEAO upon stimulation with MSU (500 μg/ml) for 6 h. **(A)** Cells stained with anti-ASC antibody for ASC specks (red) and DAPI for nuclei (blue). The concentrations of SDAO and SFEAO were 45 and 136 μg/ml, respectively. **(B)** The percentage of cells with ASC specks among all cells is presented. **(C**,**D)** ASC oligomers were extracted from the Triton X-100 insoluble part of the cell extract by DSS crosslinking and measured by immunoblotting. ASC speck was quantified by the cells with ASC specks relative to the total cells from three individual fields in each experiment. Data were from three independent experiments (**B**; mean and SEM of *n* = 3) or are representative of at least three independent experiments **(A**,**C**,**D)**. Arcsine-square root transformation of the percent data was used for statistical analysis. Statistics were analyzed using one-way ANOVA by Bonferroni post hoc test, *p < 0.05, **p < 0.01, ***p < 0.001.

After the priming process, NLRP3 assembles into an oligomer under the stimulation of some molecules. Then, ASC links to the NLRP3 oligomer through the PYD domain, and ASC molecules aggregate with each other via the PYD domain, forming a large scaffold for the combination of pro-caspase-1 at the CARD domain that can be visualized as a speck (Fernandes-Alnemri et al., 2007). The specks were visible under a microscope when the cells were stained with a fluorescent antibody recognizing ASC (Stutz et al., 2013). As shown in Figure 5A, cells treated with MSU exhibited many ASC specks. The percentage of cells with ASC specks was 22.2 ± 2.4% among the total cells when the cells were treated with MSU. However, MCC950, SDAO and SFEAO significantly decreased the percentage of cells with ASC specks to respectively 4.0 ± 1.1%,1.8 ± 0.4% and 1.7 ± 0.7% (Figure 5B). We found that both the dimerization and oligomerization of ASC were blocked by SDAO and SFEAO (Figures 5C,D). These results suggest that SDAO and SFEAO block the organization process of the NLRP3 inflammasome, thus inhibiting its activation.

### Effect of EOAA on Phosphorylation of MAPKs and IκBα

**FIGURE 6:**
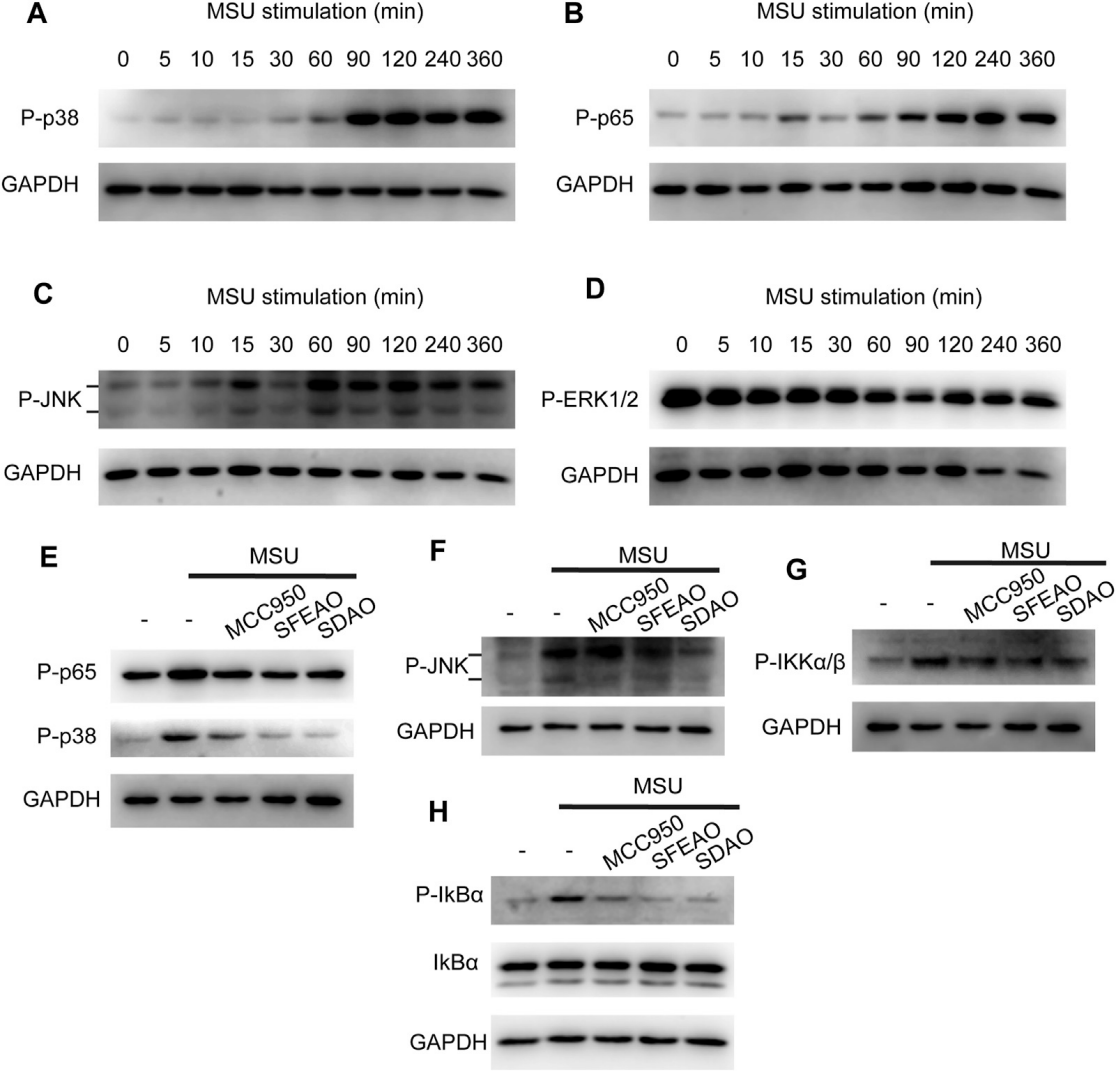
EOAA inhibited the phosphorylation of p65, p38, IKK, IkBα and JNK at 90 min after MSU treatment. PMA-primed THP-1 cells were stimulated with MSU (500 μg/ml) for different durations. The protein expression levels of p-p38 **(A)**, p-p65 **(B)**, p-JNK **(C)** and p-ERK1/2 **(D)** in the cytoplasm were measured utilizing immunoblotting. PMA-primed THP-1 cells were treated with or without MCC950 (20 µM), SDAO (45 µg/ml) or SFEAO (136 µg/ml) upon stimulation with MSU (500 µg/ml) for 90 min. The phosphorylation of p65, p38 **(E)**, JNK **(F)**, IKKα/β **(G)**, and IkBα **(H)** in whole-cell extracts was measured by immunoblotting. GAPDH was acted as internal reference.

Some researchers have reported that the MAPK signaling pathway is involved in the inflammation caused by MSU (Jaramillo et al., 2004; Campillo-Gimenez et al., 2018; Bousoik et al., 2020). In addition, the MAPK signaling pathway is upstream of NF-κB. Therefore, we detected the phosphorylation of several MAPKs, including p38, JNK1/2, ERK1/2 and p65, to determine whether MAPKs could be phosphorylated before p65. As shown in Figures 6B,C, JNK and p65 were phosphorylated 60 min after MSU treatment. p38 was significantly phosphorylated at 90 min (Figure 6A). However, the phosphorylation of ERK1/2 appeared in the resting state and decreased gradually (Figure 6D). Our data showed that MAPKs were phosphorylated after MSU treatment and that JNK and p65 were phosphorylated at almost the same time point.

Then, we detected whether EOAA inhibited the phosphorylation of p38, JNK, ERK and p65 at 90 min after MSU treatment. Figures 6E,F show that SDAO (45 µg/ml) and SFEAO (136 µg/ml) inhibited the phosphorylation of p38, JNK and p65. Further, we detected the phosphorylation of IKKα/β and IkBα, which are indicators of the initiation of NF-κB activation. Our results showed that MSU treatment increased the phosphorylation of IKKα/β and IκBα (Figures 6G,H). SDAO (45 µg/ml) and SFEAO (136 µg/ml) inhibited the phosphorylation of IKKα/β and IκBα (Figures 6G,H). These results indicate that EOAA inhibits the NF-κB and MAPK signaling pathways.

### EOAA Inhibits the Release of Processed Caspase-1 and IL-1β Induced by Nigericin

**FIGURE 7:**
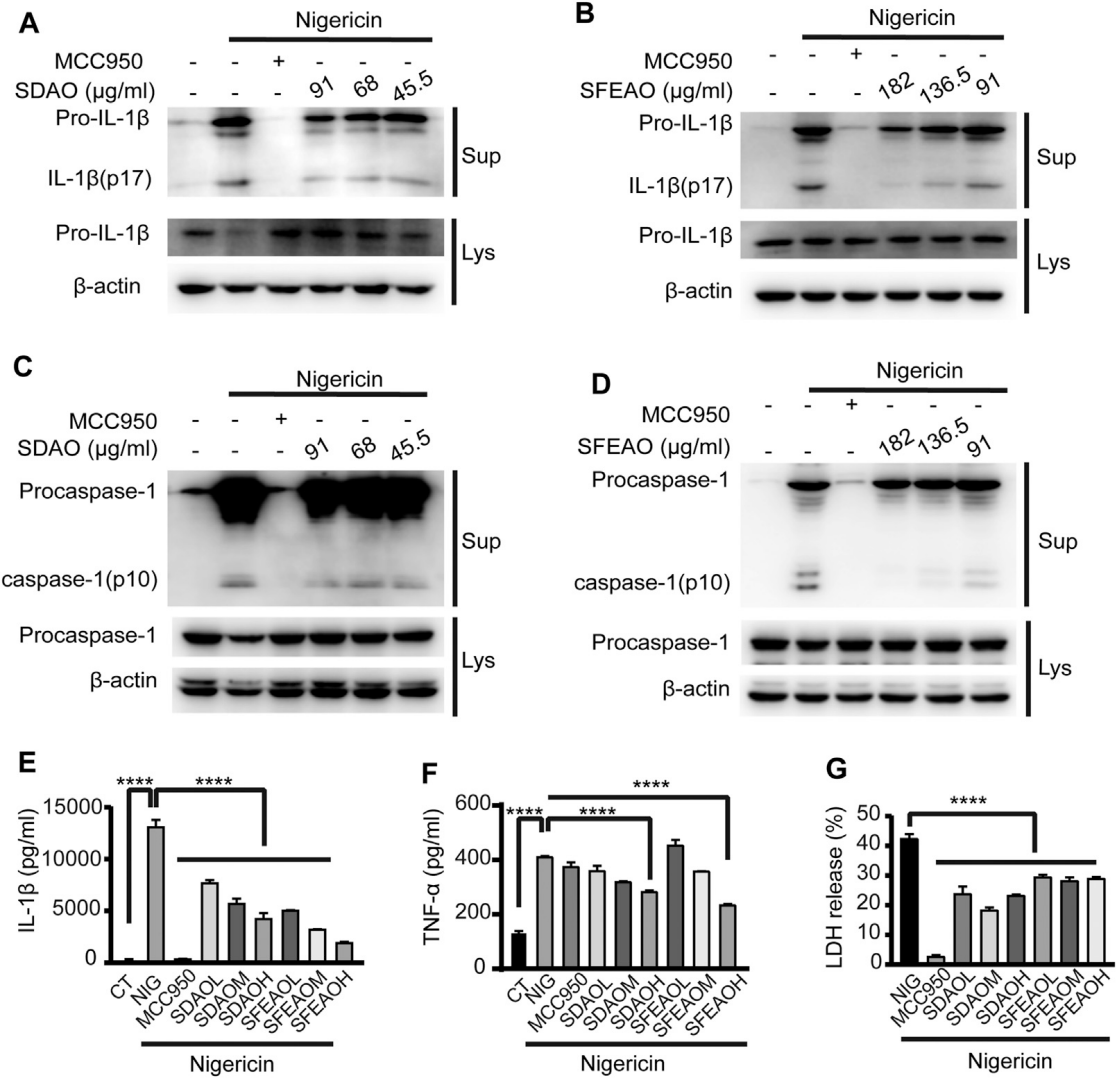
EOAA inhibits the release of processed caspase-1 and IL-1β induced by nigericin. PMA-primed THP-1 cells were treated with or without MCC950 (20 μM) or different concentrations of SDAO or SFEAO and stimulated with nigericin (10 μM) for 1 h. Mature IL-1β and caspase-1 cleavage were measured in the supernatant. Pro-IL-1β **(A**,**B)** and procaspase-1 **(C**,**D)** levels were measured in whole-cell extracts by immunoblotting. β-Actin was used as an internal control. IL-1β **(E)** and TNF-α **(F)** secretion was determined by ELISA. The release of LDH in cell supernatants was assessed by LDH assay **(G)**. CT:Ctrl; NIG:nigericin; SDAOL, SDAOM, SDAOH mean 45.5, 68, 91 μg/ml SDAO, respectively; SFEAOL, SFEAOM, SFEAOH mean 91, 138.5, 182 μg/ml SFEAO, respectively. Data were from three independent experiments with biological duplicates in each (**E**,**F**; mean and SD of *n* = 6) or from three independent experiments with six biological duplicates in each (**G**; mean and SD of *n* = 18) or were representative of at least three independent experiments **(A**–**D)**. Arcsine-square root transformation of the percent data was used for statistical analysis. Statistics were analyzed using one-way ANOVA by Bonferroni post hoc test, *p < 0.05, **p < 0.01, ***p < 0.001, ****p < 0.0001.

To determine whether EOAA could inhibit the activation of the NLRP3 inflammasome induced by other stimulators, we activated the NLRP3 inflammasome with nigericin. In this model, we found that high concentrations of SDAO and SFEAO were needed to significantly inhibit the activation of the NLRP3 inflammasome. The data showed that both SDAO and SFEAO decreased the level of mature IL-1β (Figures 7A,B) and caspase-1 (Figures 7C,D) in the cell culture supernatant. MCC950 and different concentrations of EOAA significantly inhibited the secretion of IL-1β (Figure 7E). The secretion of TNF-α was not induced by NLRP3 inflammasome activation after nigericin treatment (Figure 7F). Nigericin treatment induced the secretion of TNF-α in an NLRP3 inflammasome activation-independent way. In addition, MCC950 did not inhibit the secretion of TNF-α. We found that only high concentrations of SDAO and SFEAO inhibited the secretion of TNF-α. LDH release was also inhibited by MCC950 and different concentrations of SDAO and SFEAO (Figure 7G). Our results indicate that SDAO and SFEAO inhibit the activation of the NLRP3 inflammasome induced by nigericin.

### EOAA Inhibits the Cleavage of GSDMD Induced by Nigericin

**FIGURE 8:**
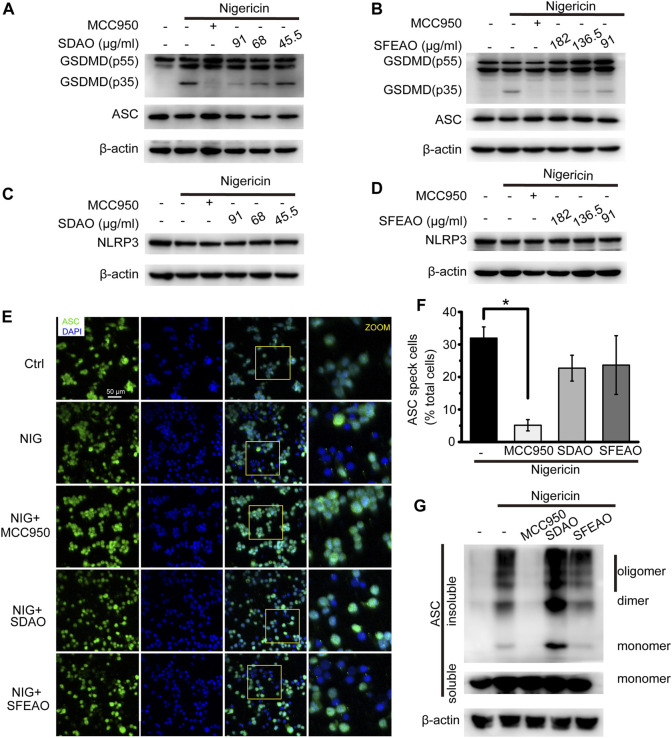
EOAA inhibits GSDMD slicing and has no effect on NLRP3 expression and the formation of ASC oligomers after nigericin treatment. PMA-primed THP-1 cells were treated with or without MCC950 (20 μM) or different concentrations of SDAO or SFEAO and stimulated with nigericin (10 μM) for 1 h. The levels of cleaved GSDMD **(A**,**B)** and NLRP3 in whole-cell extracts were measured by immunoblotting **(C**,**D)**. β-Actin was used as an internal control. PMA-primed THP-1 cells were treated with or without MCC950 (20 µM), SDAO (91 µg/ml) or SFEAO (182 µg/ml) upon stimulation with nigericin (10 µM) for 1 h. **(E)** Cells stained with anti-ASC antibody to detect ASC specks (green) and with DAPI to detect nuclei (blue). **(F)** The percentage of cells with ASC specks among all cells is presented. **(G)** ASC oligomers were extracted from the Triton X-100 insoluble part of the cell extract by DSS crosslinking and measured by immunoblotting. NIG:nigericin. ASC speck was quantified by the cells with ASC specks relative to the total cells from three individual fields in each experiment. Data were from three independent experiments (**F**; mean and SEM of *n* = 3) or representative of at least three independent experiments **(A**–**G)**. Arcsine-square root transformation of the percent data was used for statistical analysis. Statistics were analyzed using one-way ANOVA by Bonferroni post hoc test, *p < 0.05, **p < 0.01, ***p < 0.001.

Along with the inhibitory effect of SDAO and SFEAO on LDH release, we further detected the cleavage of GSDMD in nigericin-treated cells. As shown in Figures 8A,B, we found that both SDAO and SFEAO decreased the expression of the GSDMD-N fragment. However, the expression of NLRP3 was not influenced under any condition, including nigericin treatment and SDAO and SFEAO treatment (Figures 8C,D). These data indicate that SDAO and SFEAO inhibits cell pyroptosis by blocking the cleavage of GSDMD during activation of the NLRP3 inflammasome.

Surprisingly, we found by detecting ASC specks (Figures 8E,F) and oligomers (Figure 8G) that the SDAO and SFEAO did not influence oligomerization. These results indicate that SDAO and SFEAO do not influence the organization process of the NLRP3 inflammasome. Given that SDAO and SFEAO eventually inhibit the activation of the NLRP3 inflammasome, we hypothesized that EOAA might influence the downstream process of the ASC oligomerization and then inhibit the processing of caspase-1 in nigericin-treated cells.

## Discussion

*Artemisia argyi* H. Lév. and Vaniot is a traditional Chinese medicine that is effective against inflammation. However, the mechanism underlying it is not clear. Here, we demonstrated that EOAA inhibits the activation of the NLRP3 inflammasome induced by MSU and nigericin. Moreover, different mechanisms underlie the inhibitory effects of EOAA on NLRP3 inflammasome activation induced by MSU and nigericin, suggesting that components of EOAA may act on multiple targets to hinder multiple processes during NLRP3 inflammasome activation.

IL-1β is a critical proinflammatory cytokine belonging to the IL-1 cytokine family. IL-1β binds with IL-1R to induce the upregulation of MHC-II, adhesion molecules and IFN-γ in innate immune cells (Dinarello, 2009). IL-1β exists in the cytosol as a 31-kDa precursor that can be cleaved into 17–18-kDa mature forms by caspase-1 during inflammasome activation (Martinon et al., 2002). According to our results, both MSU and nigericin treatment increased the amount of the mature forms of caspase-1 and IL-1β in the cell supernatant, which was significantly decreased by EOAA. Thus, our results demonstrate that EOAA is effective at inhibiting NLRP3 inflammasome activation.

Unlike most cytokines, IL-1 cytokines lack an N-terminal secretion signal sequence and thus are not released through a classical ER–Golgi system (Piccioli and Rubartelli, 2013). Rather, processed IL-1β is alternatively released via the pyroptotic and the nonpyroptotic effects of GSDMD. Cleaved from GSDMD by active caspase-1, the GSDMD-N domain aggregates at the cell membrane, forming a pore structure with a diameter wide enough for the passage of IL-1 cytokines (He et al., 2015; Evavold et al., 2018). Furthermore, IL-1β leaks out through the ruptured cell membrane during cell pyroptosis (Kayagaki et al., 2015; Shi et al., 2015). In our research, EOAA inhibited GSDMD cleavage and pyroptosis, as indicated by the release of LDH upon stimulation with MSU and nigericin. These results suggest that EOAA could contribute to suppressing the secretion of IL-1β by blocking the cleavage of GSDMD and the subsequent induction of pyroptosis.

*Artemisia argyi* H. Lév. and Vaniot is a well-known medical herb with strong aromatic odours. It contains multiple phytochemicals including essential oil, flavonoids, organic acids, coumarins, and polysaccharides, among which essential oil is the main composition (Ge et al., 2016). Previous studies have demonstrated the anti-inflammatory effect of *Artemisia argyi* H. Lév. and Vaniot extract. *Artemisia argyi* H. Lév. and Vaniot extract from different research groups showed significant inhibitory effect on dermatitis related lesions (Han et al., 2016; Yun et al., 2016). *Artemisia argyi* H. Lév. and Vaniot extract could play a protective role on gastric mucosa in ethanol-induced rat model by decreasing the amount of inflammatory mediators, superoxide dismutase, and malonaldehyde (Li et al., 2018), suggesting that *Artemisia argyi* H. Lév. and Vaniot contains effective anti-inflammatory components.

Essential oil is the main component of *Artemisia argyi* H. Lév. and Vaniot, and it can be extracted via steam distillation and CO_2_ supercritical fluid. EOAA is mainly composed of abundant monoterpenoids, sesquiterpenoids, alcohols, ketones, ethers, phenols and a small quantity of aldehydes, organic acids, esters, and aromatic compounds (Song et al., 2019). In our research, a total of 29 compounds and 26 compounds were identified in SFEAO and SDAO separately using GC–MS. Additionally, monoterpenoids, sesquiterpenoids, took up large proportions of SFEAO and SDAO. These results are in line with those of previous studies (Ge et al., 2016; Guan et al., 2019). The reported chemical composition of essential oil of *Artemisia argyi* H. Lév. and Vaniot varied with localities of growth (Pan et al., 1992), extracting and processing methods of *Artemisia argyi* H. Lév. and Vaniot (Guan et al., 2019). *Artemisia argyi* H. Lév. and Vaniot used in our research was grown at Nanyang City in Henan Province, one of the main producing places of *Artemisia argyi* H. Lév. and Vaniot in China. *Artemisia argyi* H. Lév. and Vaniot from Qichun City in Hubei Province, named as Qi Ai, has long been considered as geo-authentic medial plant (Ge et al., 2016). Thus, many researches on essential oil of Qi Ai have been conducted. Extraction methods and experimental parameters may cause the deviation on chemical composition analysis in different researches on essential oil of Qi Ai. However, the dominant chemical compounds in essential oil of Qi Ai were 1,8-cineole, β-caryophyllene, α-thujone, borneol, terpinen-4-ol, cis-sabinol, caryophyllene oxide and so on (Zheng-You et al., 2009). Compared with the research of Xiao Guan et al. on chemical compounds in essential oil of Qi Ai extracted by CO2 subcritical fluid (Guan et al., 2019), 18 common compounds, accounting for 41.15% of the whole content, were found in SFEAO of our study. 12 compounds making up a portion of 53.24% in SDAO of our study were same as that in essential oil of Qi Ai. The common compounds separately accounted for 58.68 and 51.51% of essential oil of Qi Ai obtained by subcitical extraction and hydrodistillation. These results indicate that the main chemical composition of essential oil in our research is similar to that of Qi Ai. However, 1,8-cineole and camphor, the major compounds in SFEAO and SDAO, were not detected in research of Xiao Guan et al. Thujone, a common compound made up a portion less than 1% in SFEAO and SDAO, accounted for 7.989 and 11.312% in essential oil of Qi Ai obtained by subcitical extraction and hydrodistillation. Thus, the proportion of some common compounds in essential oil of *Artemisia argyi* H. Lév. and Vaniot variated with its producing area. Remarkably, compared with the research of essential oil extracted from Nanyang Artemisia argyi (Duan et al., 2017), we also found many major common compounds with different percentages in SFEAO and SEAO.

EOAA has also been reported to inhibit the release of proinflammatory mediators (NO, PGE2 and ROS) and cytokines (TNF-α, IL-6, IFN-β and MCP-1) in LPS-induced RAW264.7 macrophages, suggesting that EOAA has anti-inflammatory potential. In addition, EOAA inhibited the release of cytokines by inhibiting the phosphorylation of JAK2 and STAT1/3, but not the activation of MAPK and NF-κB (Chen et al., 2017). Contrary to this report, our research found that EOAA inhibits the phosphorylation of p65 and the translocation of NF-κB upon treatment with MSU. The reason for these opposite results may attribute to the difference of cell model and essential oil components. The amount of eucalyptol, also known as 1,8-cineole, account for most in both our research and Chen et al. (2017)’s. However, it was quantified to 33.4% in Chen et al. (2017)’ study, which was 12.3% in our study. Meanwhile, the five most abundant compounds were eucalyptol, terpinen-4-ol, β-caryophyllene, borneol, and alpha-terpineol in SFEAO and SDAO, which is different from eucalyptol, cyclohexanol, α -(-)-thujone, camphor, (-)-borneol in Chen et al. (2017)’ study.

Previous studies showed that multiple factors interfered with the assembly of NLRP3 inflammasome, including but not limited to the amount of NLRP3 protein (Bauernfeind et al., 2009), post-translational modification and self-oligomerization of NLRP3 (Py et al., 2013), the interaction of ASC and NLRP3 (Lu et al., 2014), phosphorylation of ASC (Hara et al., 2013) and interaction of NLRP3 with other regulatory proteins (Zhou et al., 2010). In addition to being a transcription factor of inflammatory genes, NF-κB plays a vital role in priming NLRP3 activation. In the resting state, the intracellular amount of NLRP3 and pro-IL-1β is not sufficient to initiate activation of the NLRP3 inflammasome. Activation of NF-κB mediated by pattern recognition and cytokine receptors upregulates the transcription of pro-IL-1β and NLRP3, which is necessary but not sufficient for NLRP3 activation (Bauernfeind et al., 2009). Our results showed that EOAA inhibits the activation of NF-κB by blocking the phosphorylation of its upstream kinase IKK, resulting in low gene expression of NLRP3 after MSU treatment. However, only SFEAO mildly inhibited the protein expression of NLRP3. Thus, the main reason for EOAA inhibit the activation of NLRP3 inflammasome is not lowing the protein expression of NLRP3. Meanwhile, MSU only induced a less than 2-fold increase of NLRP3 at mRNA and protein expression. THP-1 cells used in our study were primed with PMA at 0.5 μM for 3 h to differentiate into macrophages, which may mask the effect of other stimuli (Park et al., 2007).

Besides, phosphorylation of NLRP3 also indeed regulated the assembly of NLRP3 inflammasome. JNK1-mediated NLRP3 S194 phosphorylation induced NLRP3 deubiquitylation, which is essential for its oligomerization and assembly with ASC (Song et al., 2017). Phosphorylation of NLRP3 on Ser295 by PKD at the Golgi is required for release NLRP3 from MAMs, resulting in assembly of the active inflammasome (Zhang et al., 2017). However, it is contradictory about the effect of phosphorylation on NLRP3. NLRP3 phosphorylation at S295 via NPR1/cGMP/PKG-1 axis contributed to disassembly of NLRP3 inflammasome (Mezzasoma et al., 2020). Additionally, the activation of JNK1 drive the phosphorylation of ASC at CARD domain, which is critical for the assembling of ASC speck (Hara et al., 2013).

Previous studies have noted that MSU treatment induces the phosphorylation of JNK, p38, and ERK1/2, which contributes to the activation of NF-κB and the inflammatory reaction of MSU (Jaramillo et al., 2004; Campillo-Gimenez et al., 2018; Bousoik et al., 2020). The results of this study showed that MSU treatment induced the phosphorylation of p38 and JNK and slightly decreased the phosphorylation of ERK1/2. EOAA inhibited the phosphorylation of both p38 and JNK in MSU-stimulated THP-1 cells. Therefore, it is possible that EOAA inhibit the phosphorylation of NLRP3 or ASC due to phosphorylation of JNK, which need to be proved based on credible experimental evidence.

Nigericin is an inducer of the NLRP3 inflammasome that causes K^+^ influx and initiates its structural organization (Perregaux and Gabel, 1994). In PMA-induced THP-1 cells, nigericin activated the NLRP3 inflammasome. Administration of EOAA to PMA-induced THP-1 cells before nigericin treatment did not influence NLRP3 inflammasome priming but interfered with the subsequent activation procedure. Our results showed that EOAA did not inhibit the oligomerization of ASC but indeed blocked the activation of the NLRP3 inflammasome, as indicated by the blockade of caspase-1 cleavage. IL-1β and GSDMD cleavage and the release of LDH are the results of caspase-1 activation. Thus, EOAA might block the process of caspase-1 activation.

At present, it is unclear how EOAA blocks the process of caspase-1 activation. There are many ways to block this process, such as interfering with the binding of ASC to caspase-1 or inactivating the enzymatic activity of caspase-1 directly. In addition, we did not study whether EOAA could inhibit the activation of other forms of inflammasomes, such as AIM and NLRC4 inflammasomes and the nonconventional NLRP3 inflammasome. Caspase-1 is the common effector of the above inflammasomes. Our data indicate that EOAA could inhibit the activation of caspase-1 without interfering with the oligomerization of ASC. Thus, EOAA might inhibit the activation of other inflammasomes. However, more experiments need to be done to verify this assumption.

## Data Availability

The raw data supporting the conclusions of this article will be made available by the authors, without undue reservation.
