# Supplementary figures and images for: The Essential Oil of Artemisia argyi H.Lév. and Vaniot Attenuates NLRP3 Inflammasome Activation in THP-1 Cells
Source: Front Pharmacol. 2021 Sep 16;12:712907. doi: 10.3389/fphar.2021.712907 (PMC8481632; doi:10.3389/fphar.2021.712907)

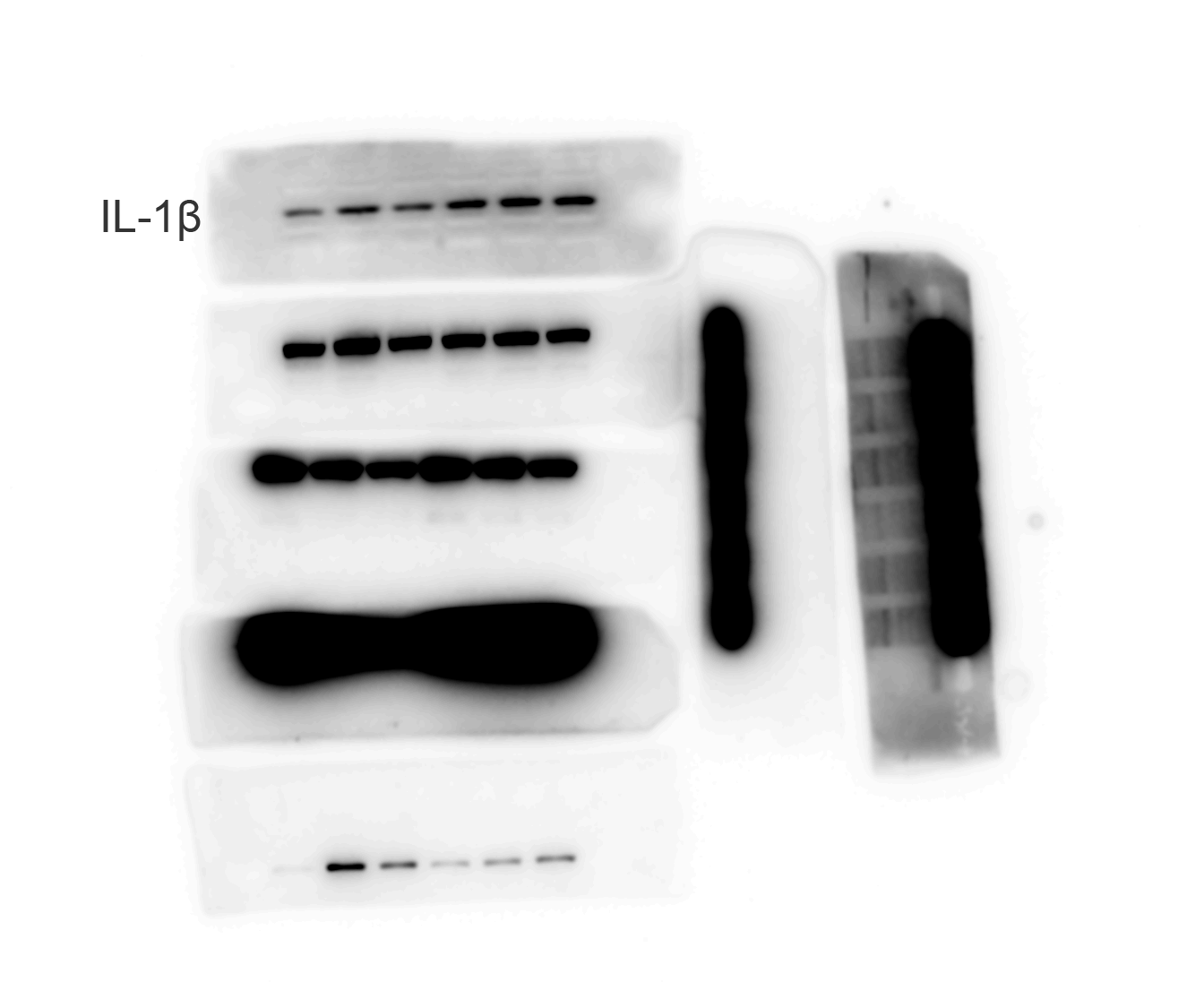

Supplement: Supplementary file 1 [file DataSheet1.ZIP › original WB figures/Figure2/Figure2A-IL-1beta-in Lys.tif]

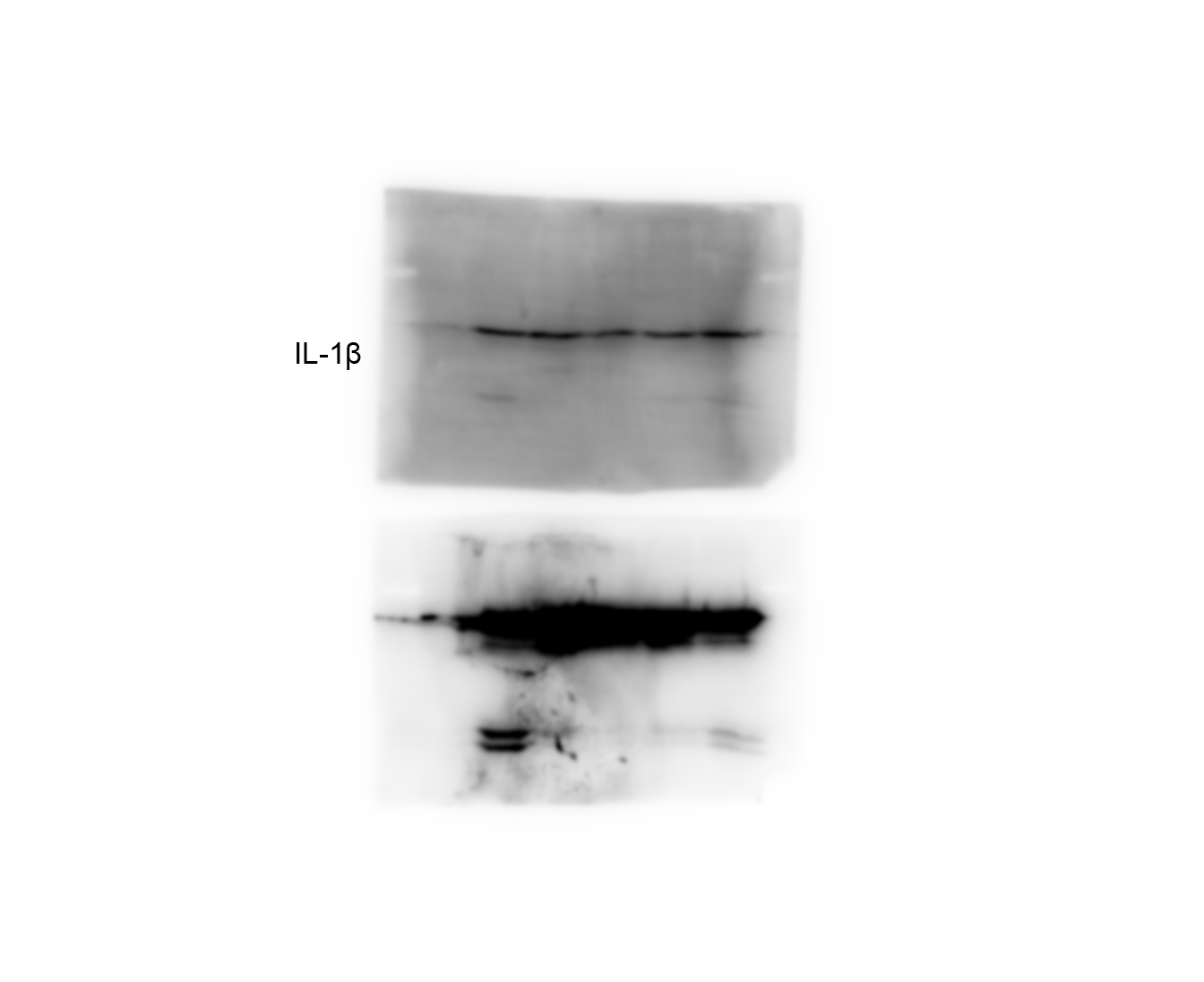

Supplement: Supplementary file 1 [file DataSheet1.ZIP › original WB figures/Figure2/Figure2A-IL-1beta-in Sup.tif]

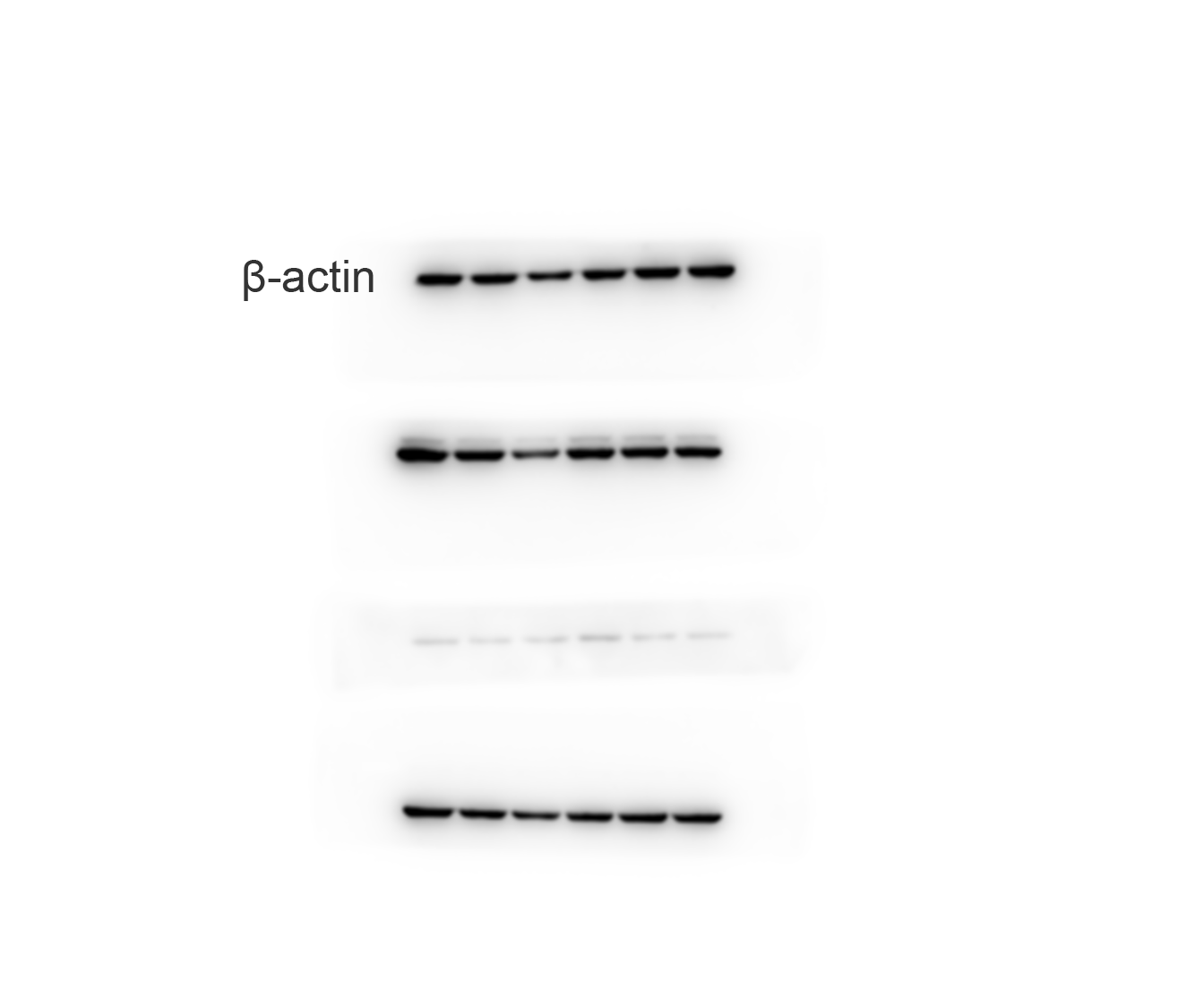

Supplement: Supplementary file 1 [file DataSheet1.ZIP › original WB figures/Figure2/Figure2A-β-actin-in Lys.tif]

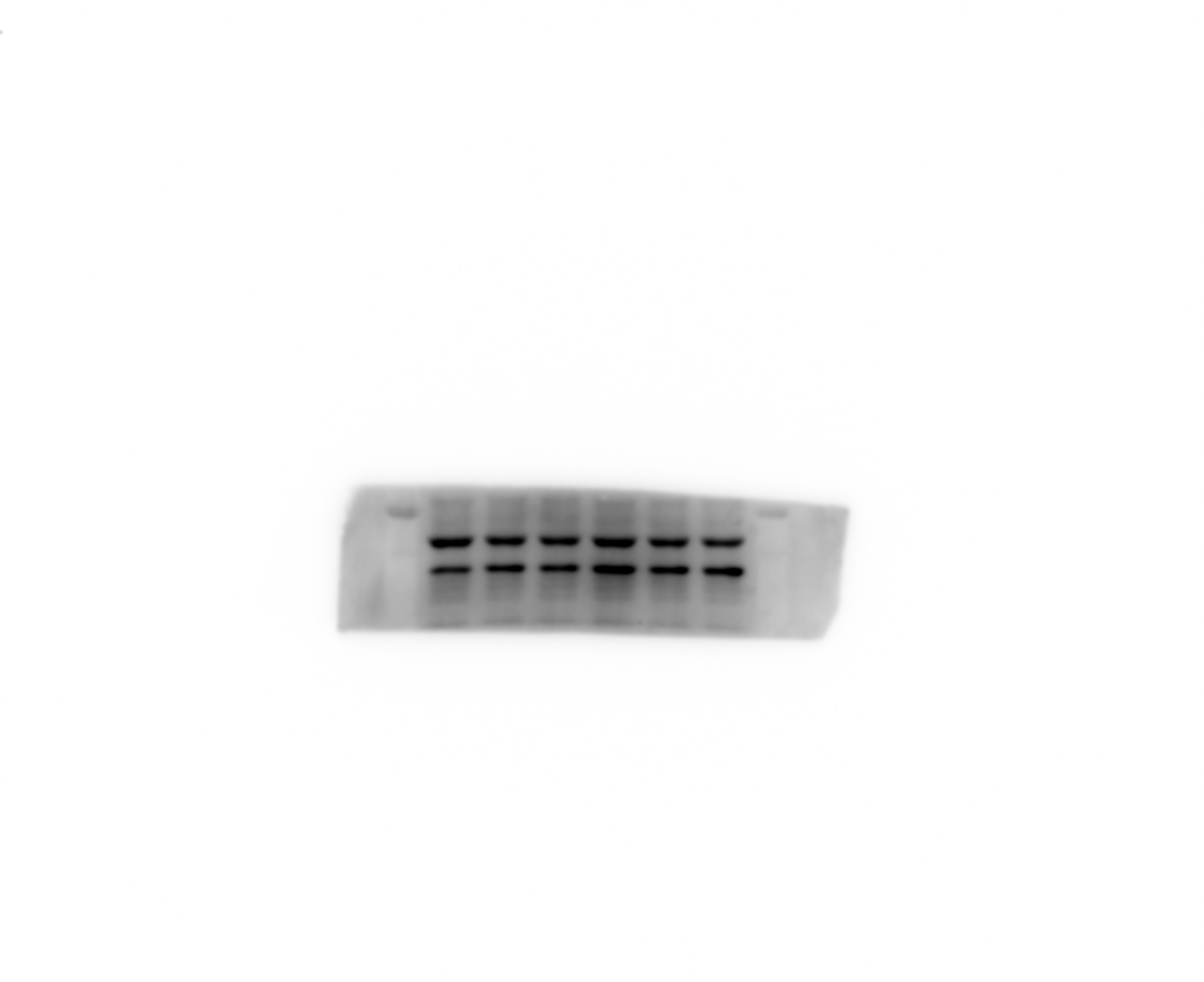

Supplement: Supplementary file 1 [file DataSheet1.ZIP › original WB figures/Figure2/Figure2B-IL-1β-in Lys.tif]

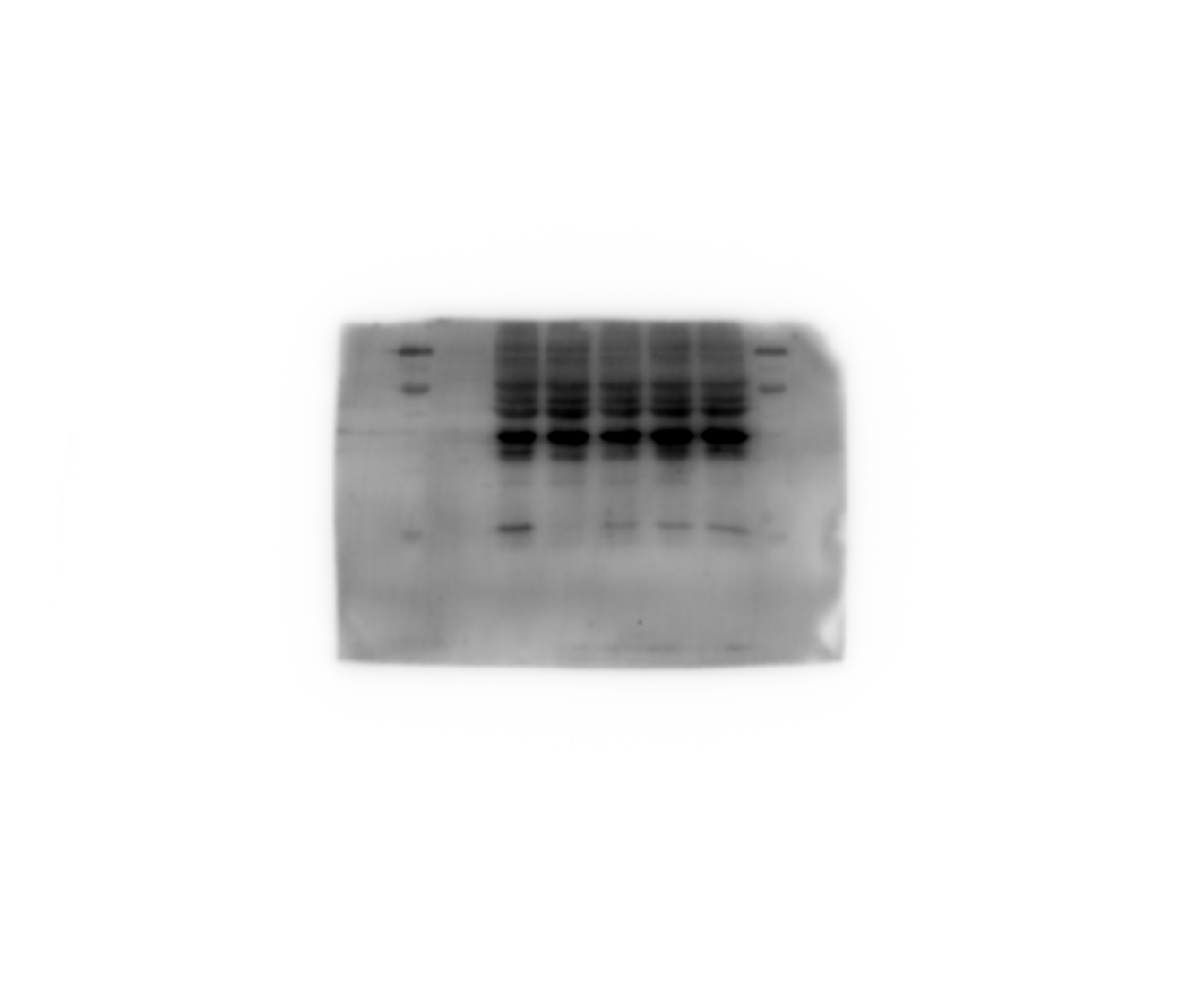

Supplement: Supplementary file 1 [file DataSheet1.ZIP › original WB figures/Figure2/Figure2B-IL-1β-in Sup.tif]

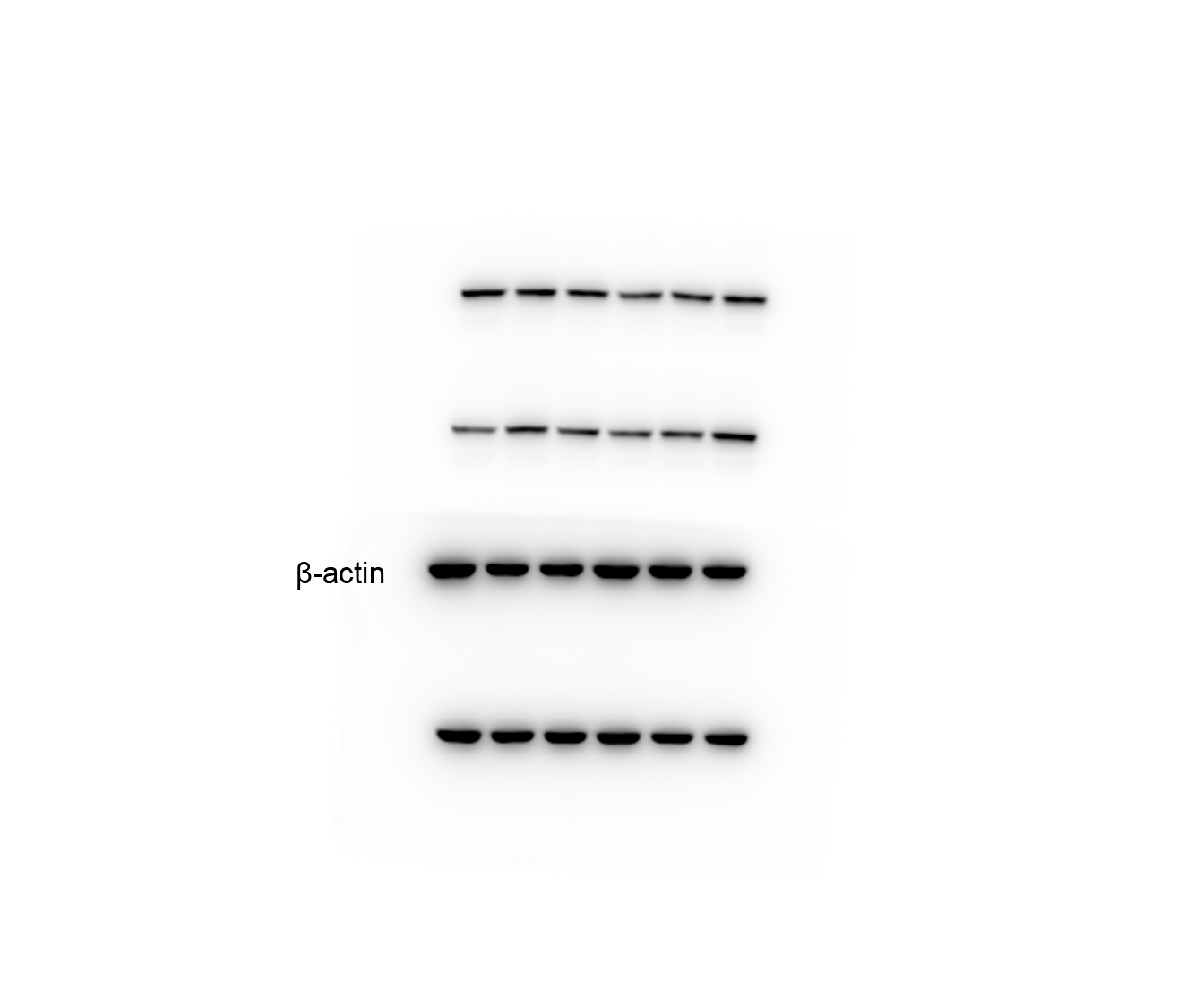

Supplement: Supplementary file 1 [file DataSheet1.ZIP › original WB figures/Figure2/Figure2B-β-actin-in Lys.tif]

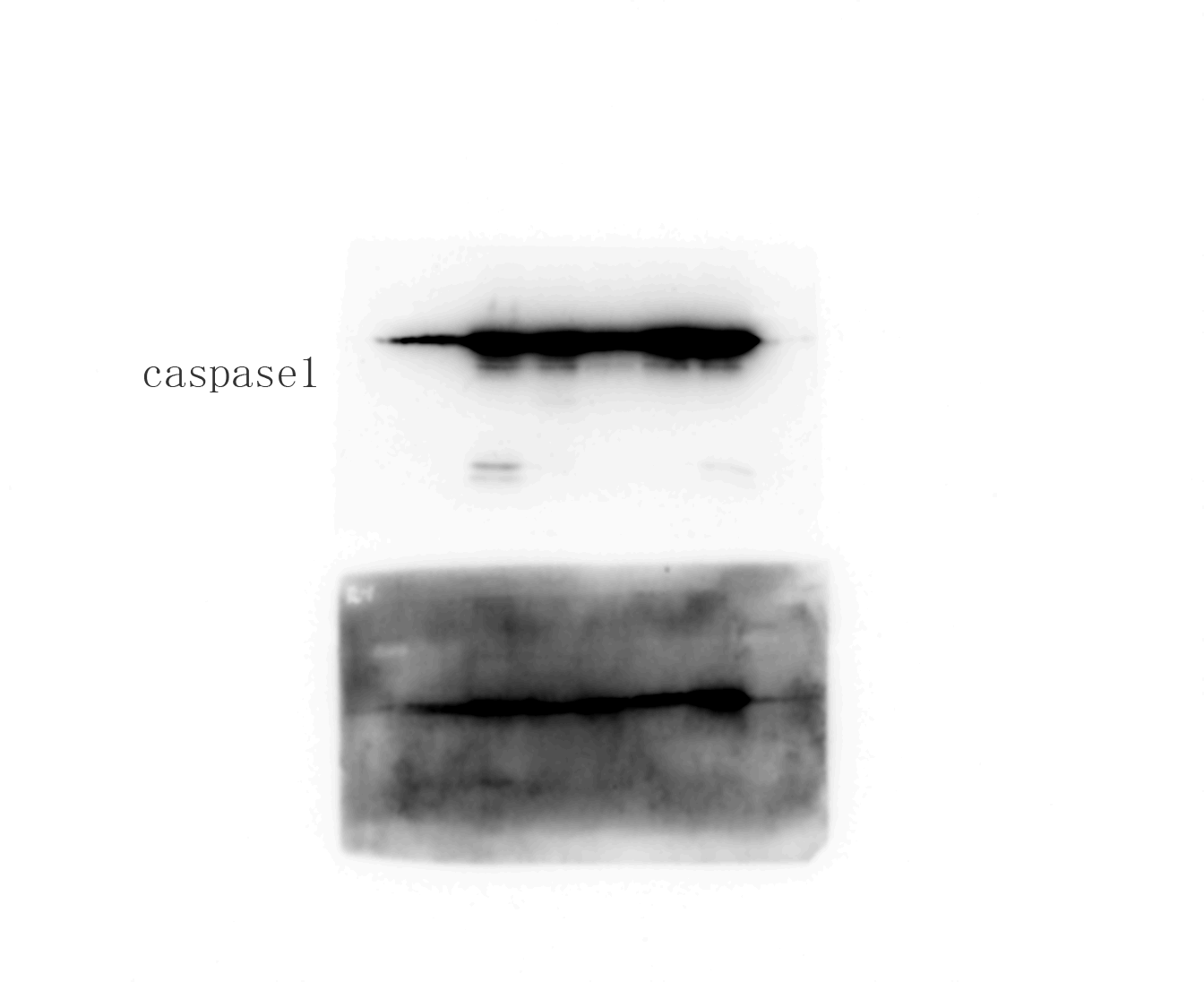

Supplement: Supplementary file 1 [file DataSheet1.ZIP › original WB figures/Figure2/Figure2C-procaspase1-in Sup.tif]

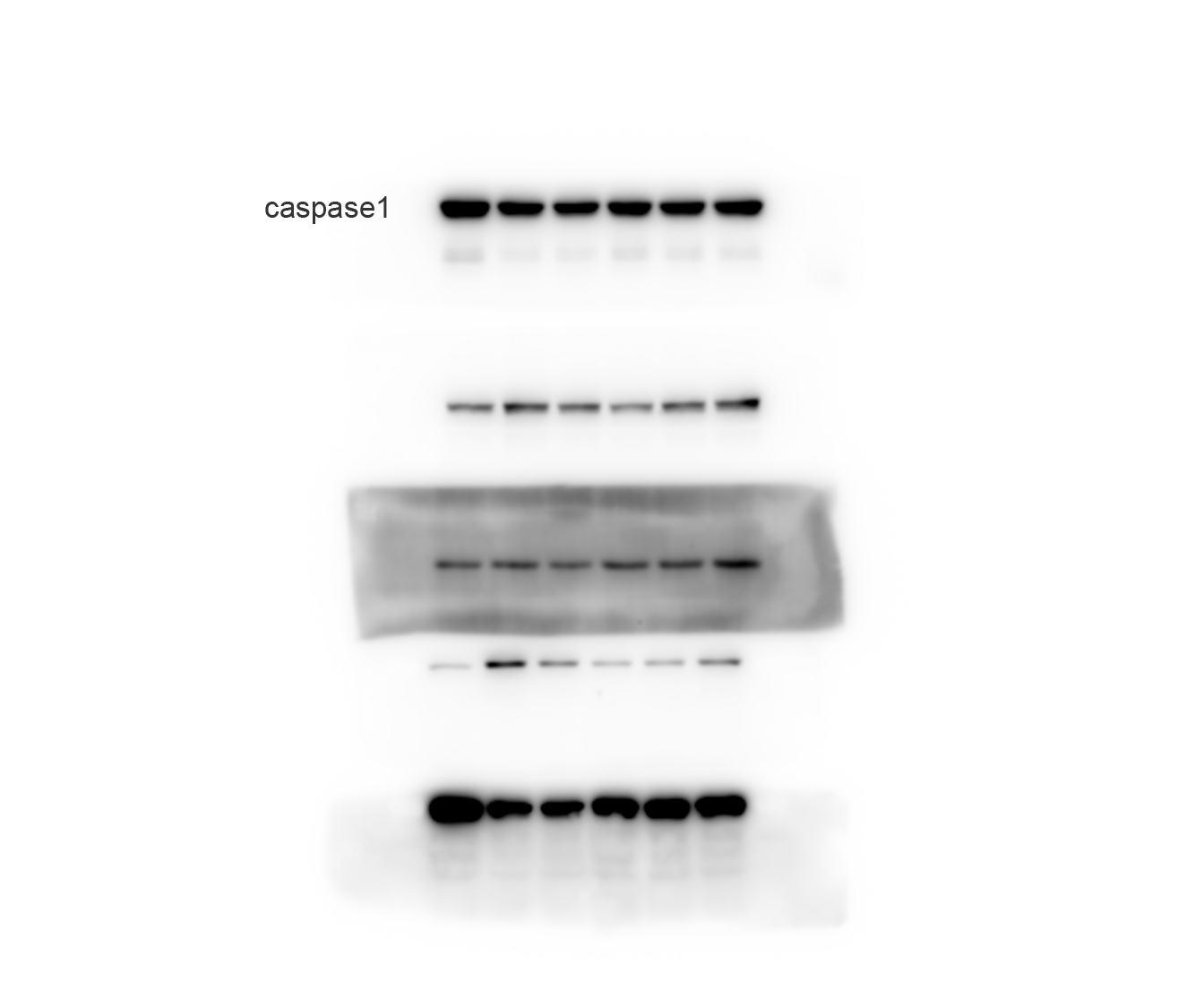

Supplement: Supplementary file 1 [file DataSheet1.ZIP › original WB figures/Figure2/Figure2C-procaspse1-in Lys.tif]

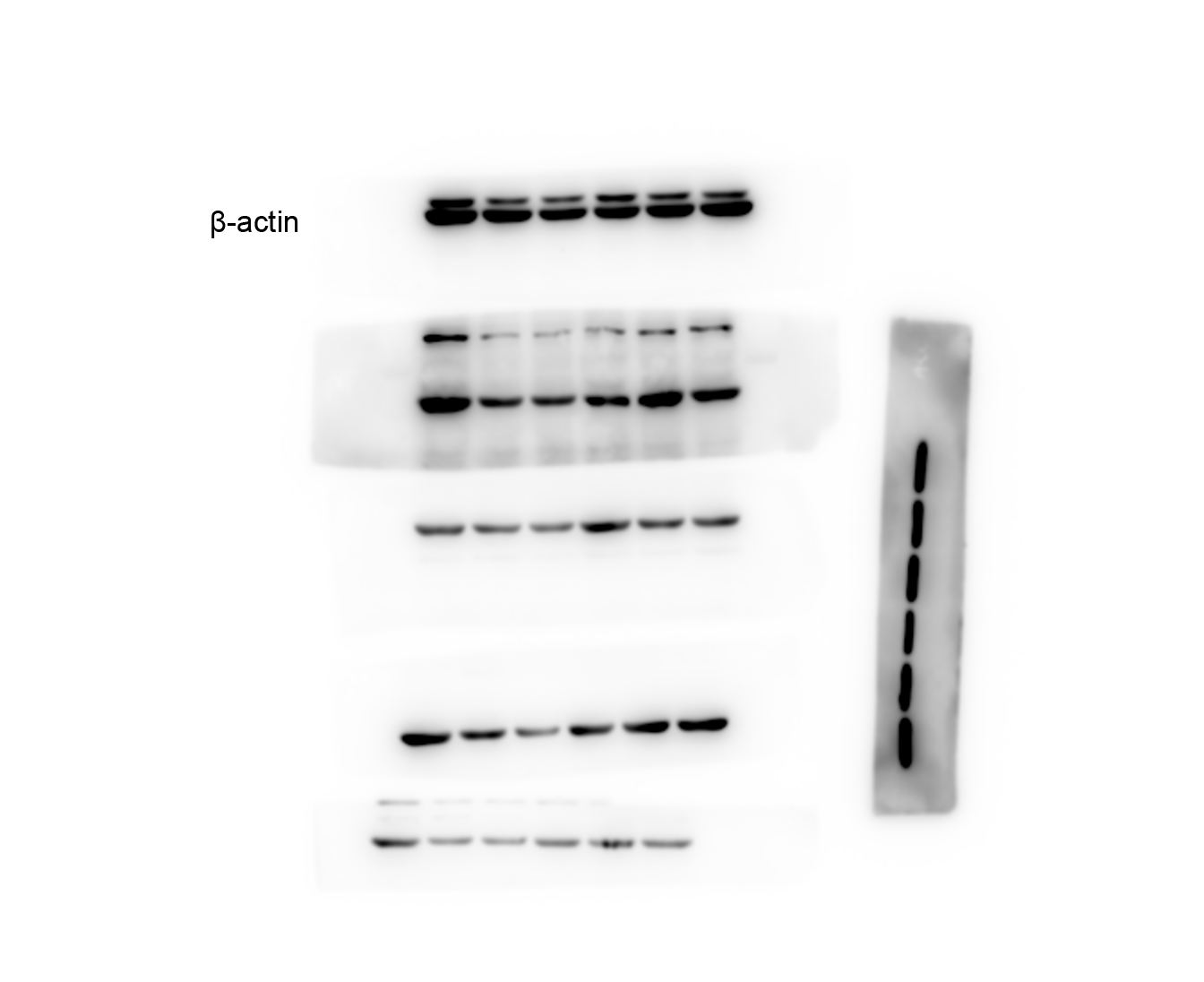

Supplement: Supplementary file 1 [file DataSheet1.ZIP › original WB figures/Figure2/Figure2C-β-actin-in Lys.tif]

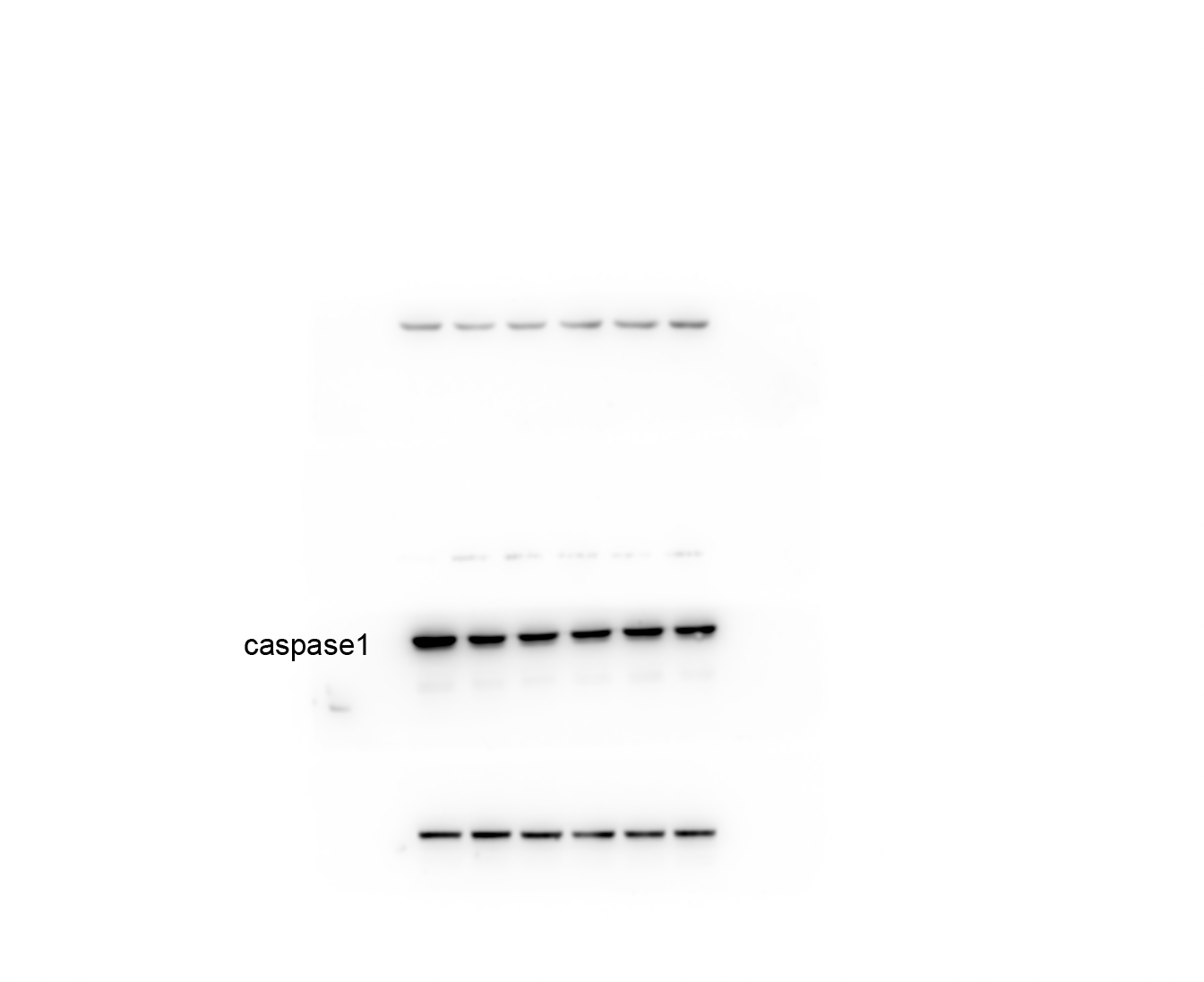

Supplement: Supplementary file 1 [file DataSheet1.ZIP › original WB figures/Figure2/Figure2D-caspase1-in Lys.tif]

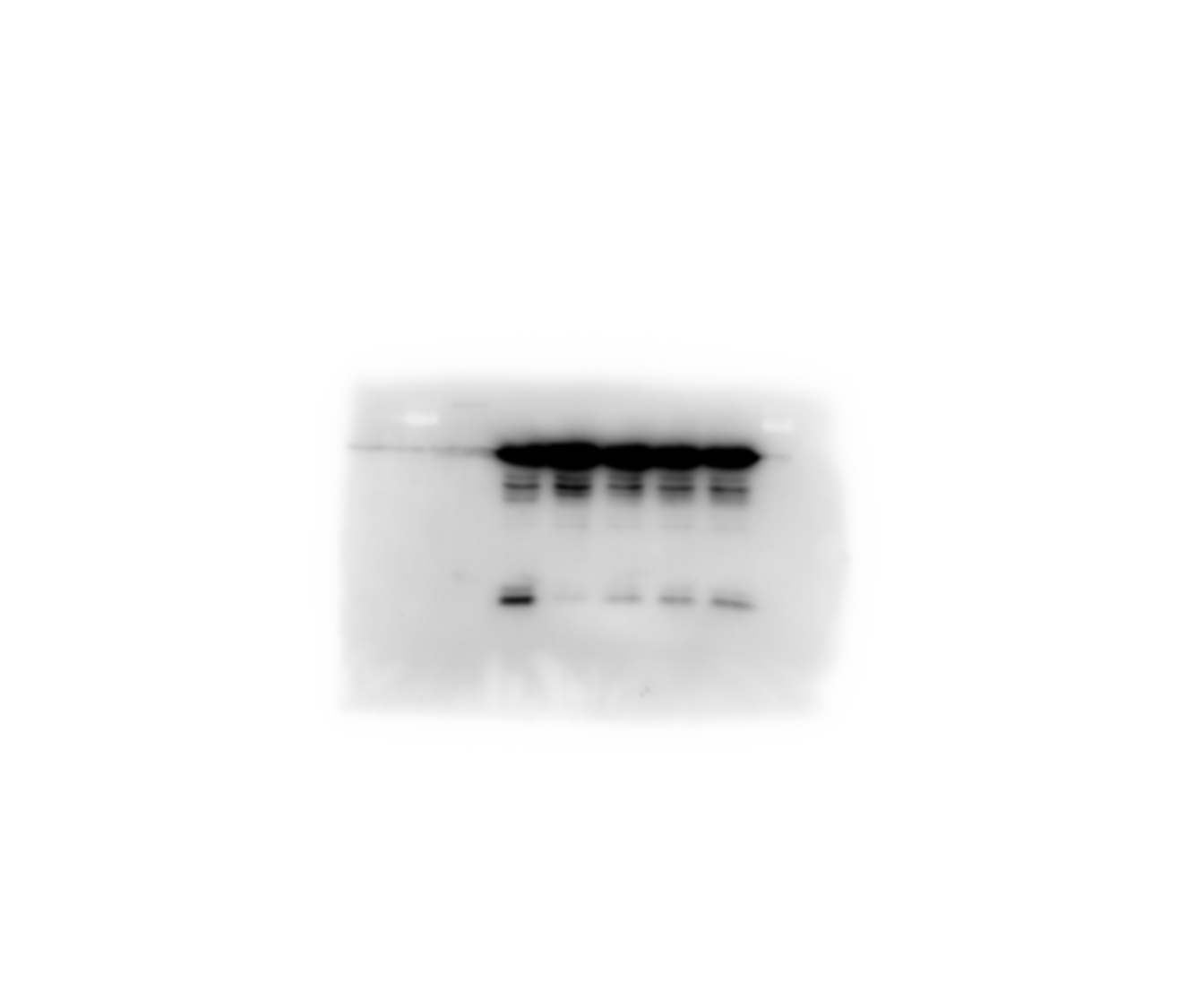

Supplement: Supplementary file 1 [file DataSheet1.ZIP › original WB figures/Figure2/Figure2D-caspase1-in Sup.tif]

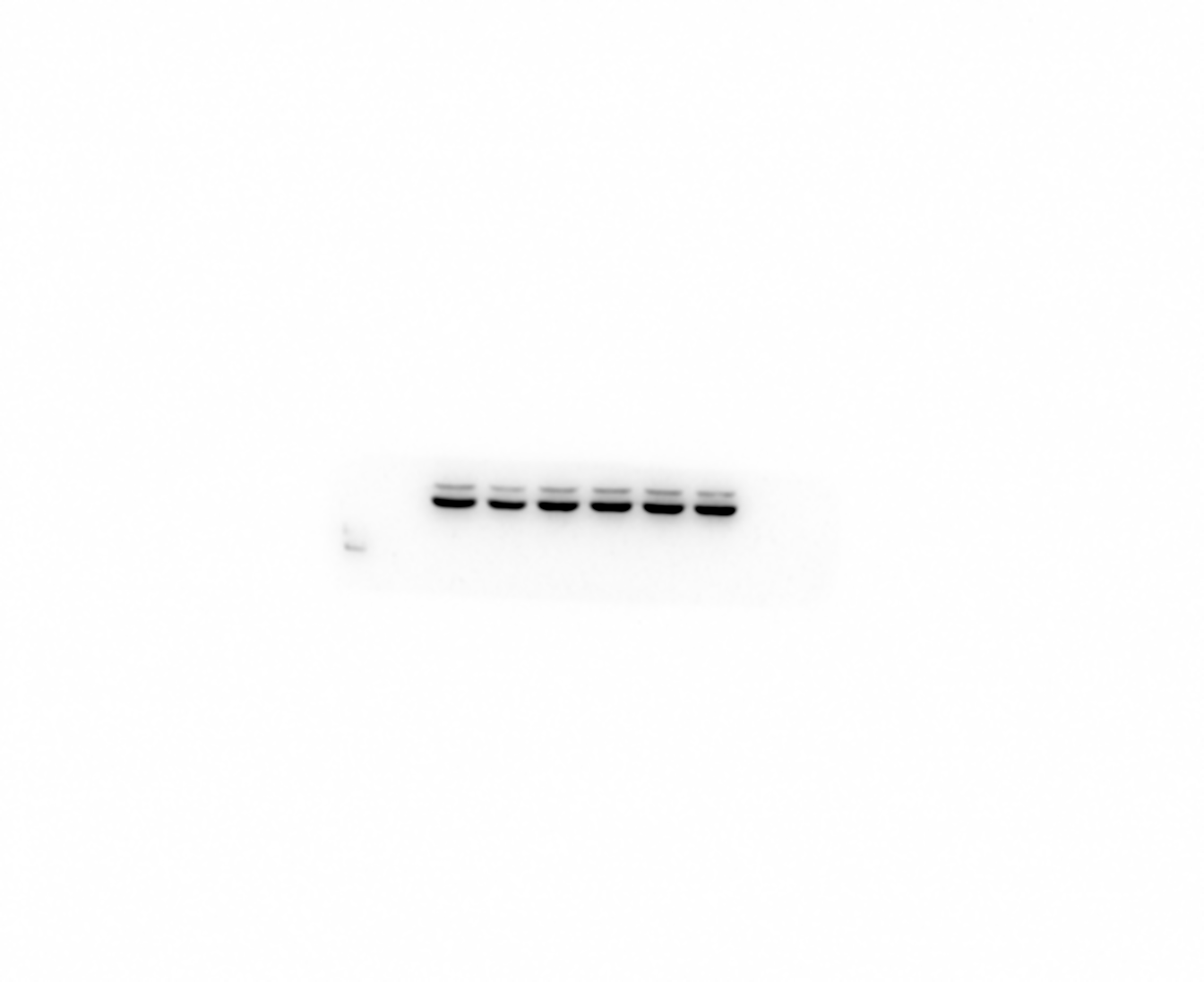

Supplement: Supplementary file 1 [file DataSheet1.ZIP › original WB figures/Figure2/Figure2D-β-actin-in Lys.tif]

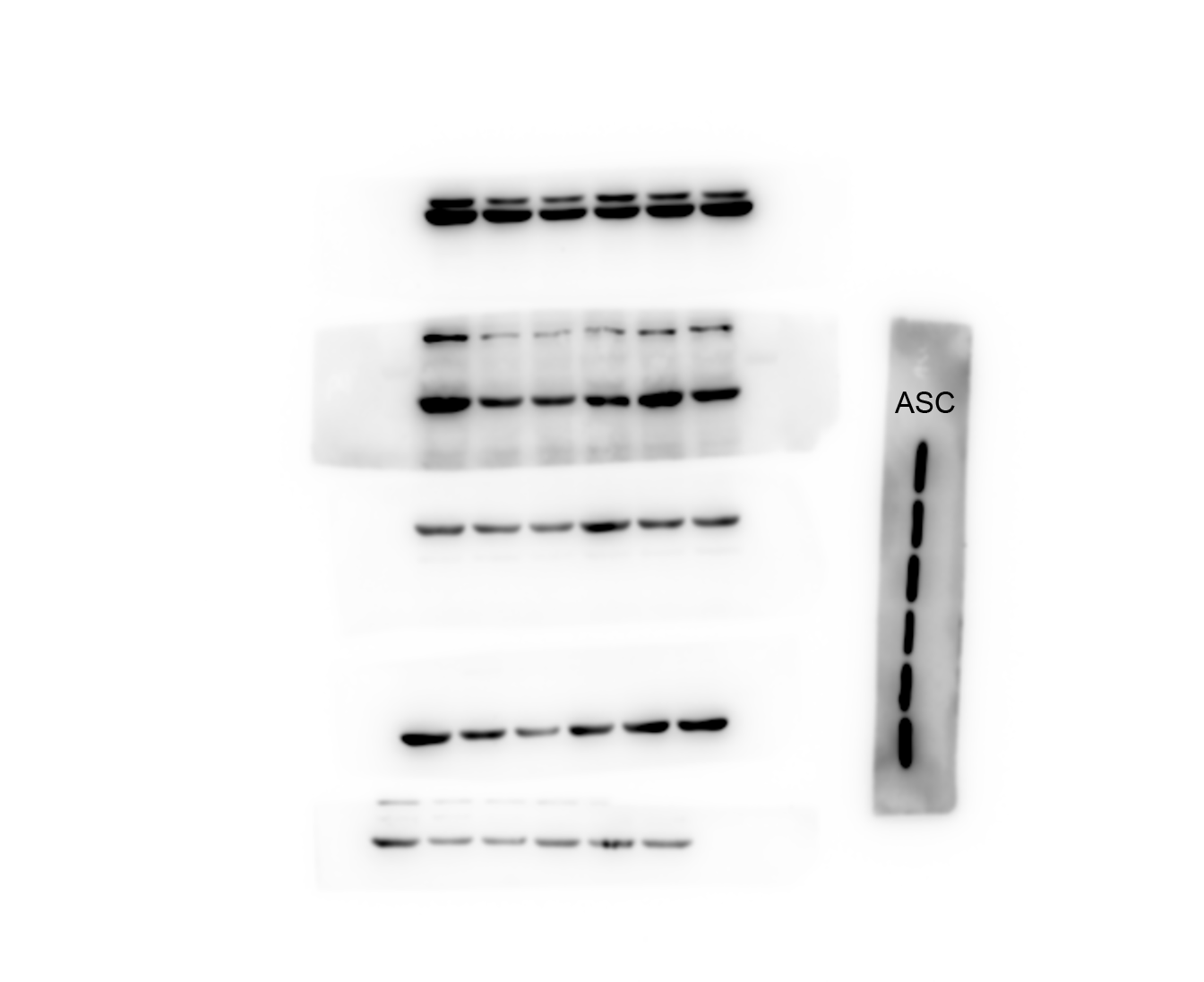

Supplement: Supplementary file 1 [file DataSheet1.ZIP › original WB figures/Figure3/Figure3C-ASC.tif]

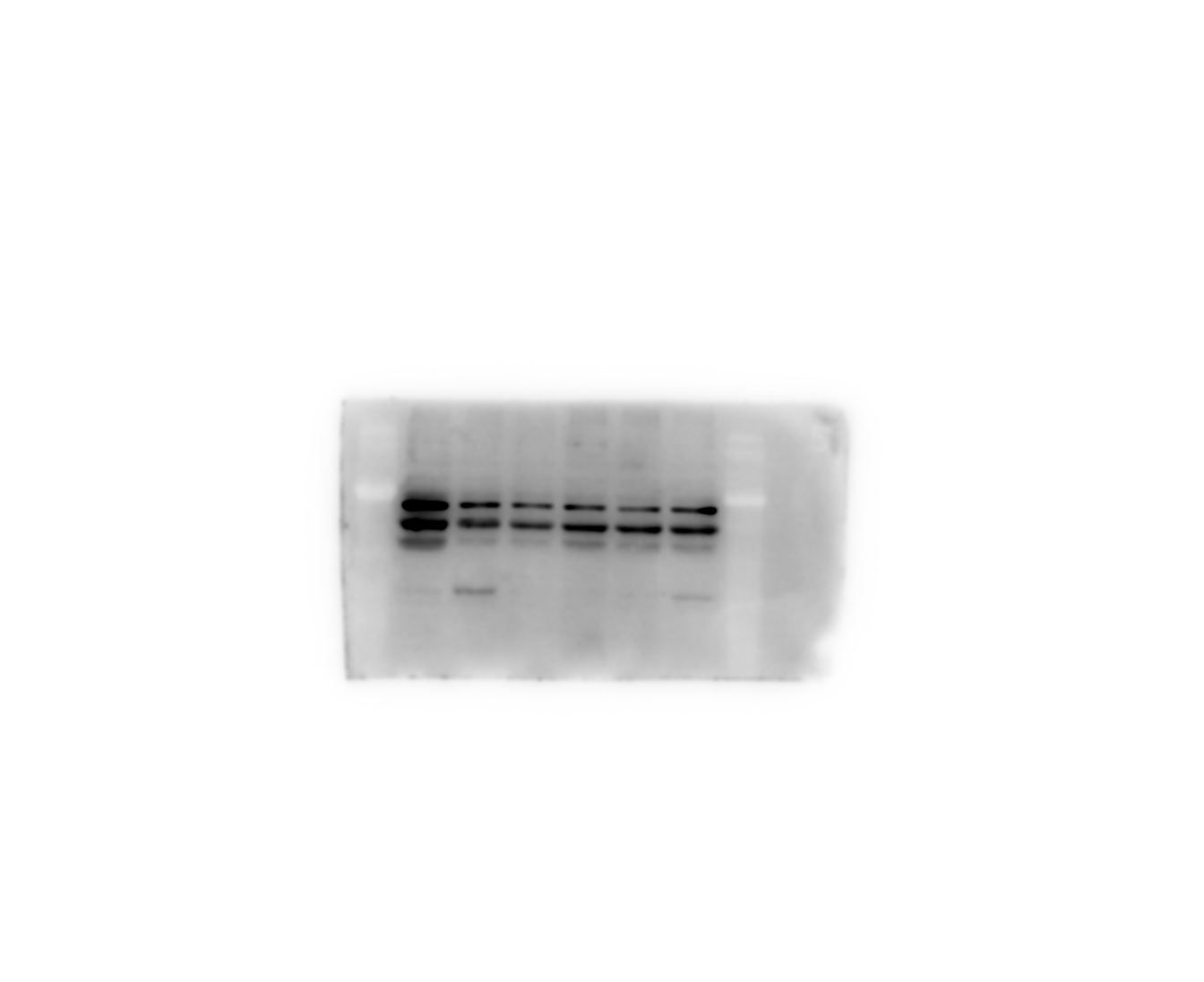

Supplement: Supplementary file 1 [file DataSheet1.ZIP › original WB figures/Figure3/Figure3C-GSDMD.tif]

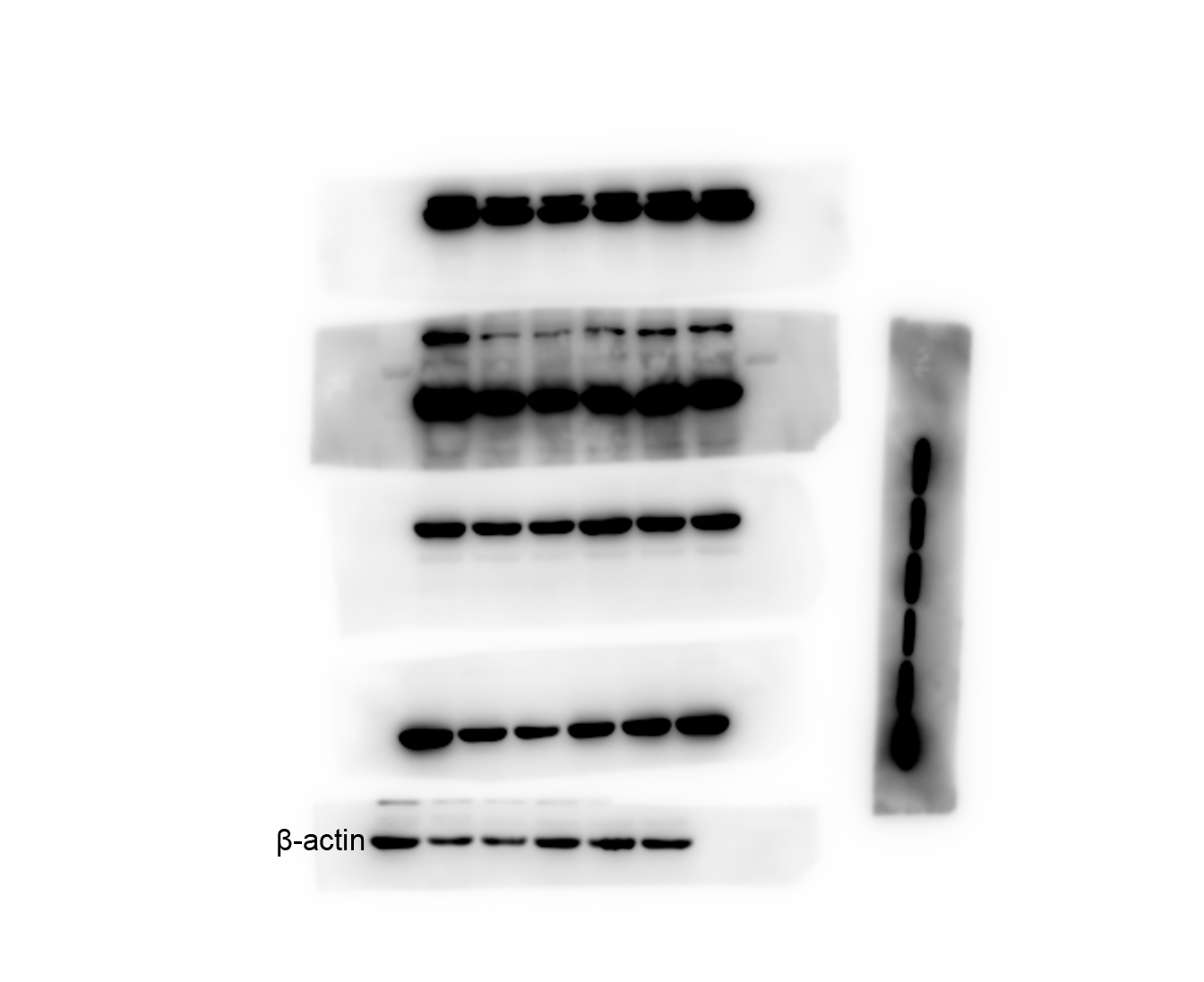

Supplement: Supplementary file 1 [file DataSheet1.ZIP › original WB figures/Figure3/Figure3C-β-actin.tif]

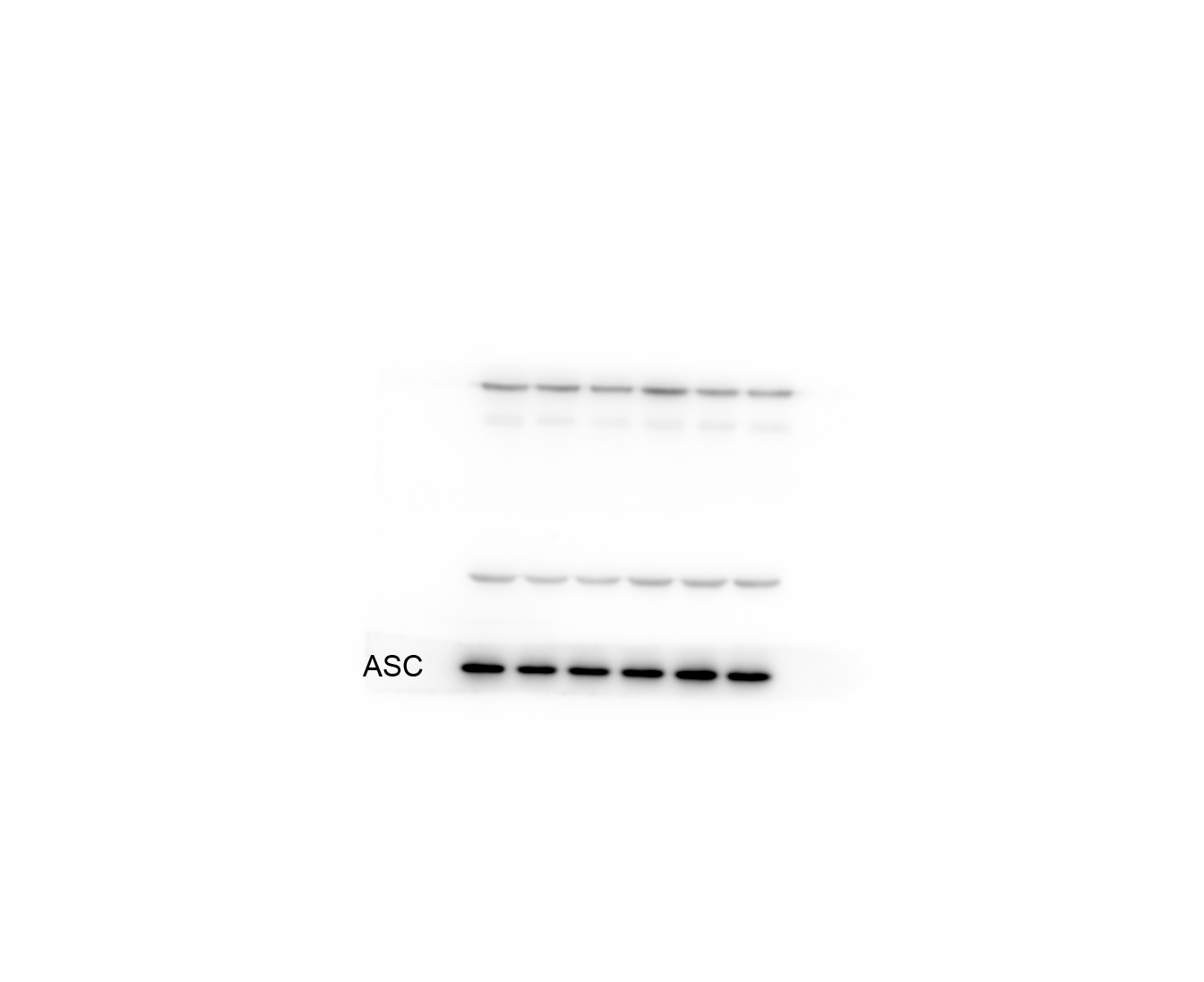

Supplement: Supplementary file 1 [file DataSheet1.ZIP › original WB figures/Figure3/Figure3D-ASC.tif]

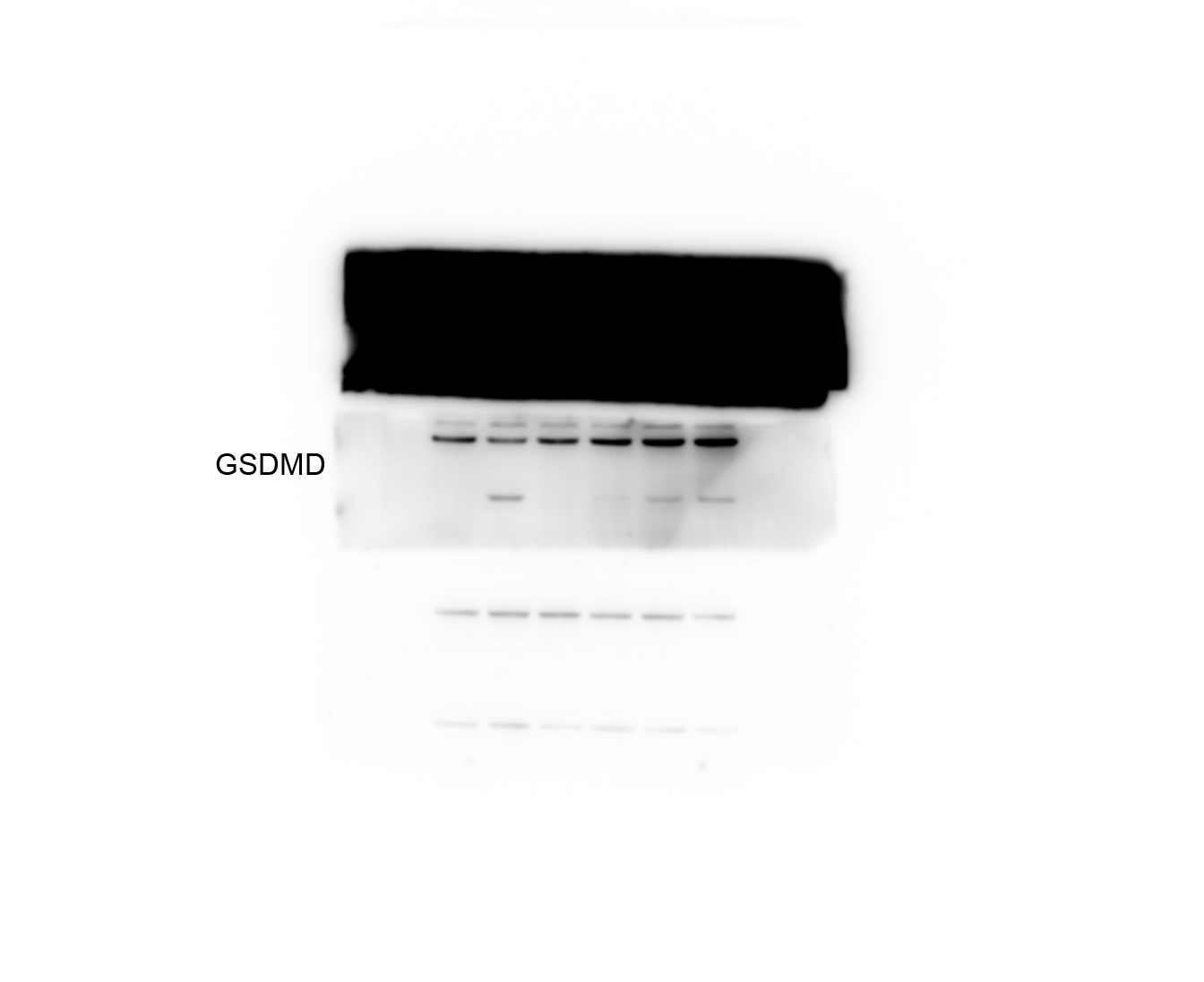

Supplement: Supplementary file 1 [file DataSheet1.ZIP › original WB figures/Figure3/Figure3D-GSDMD.tif]

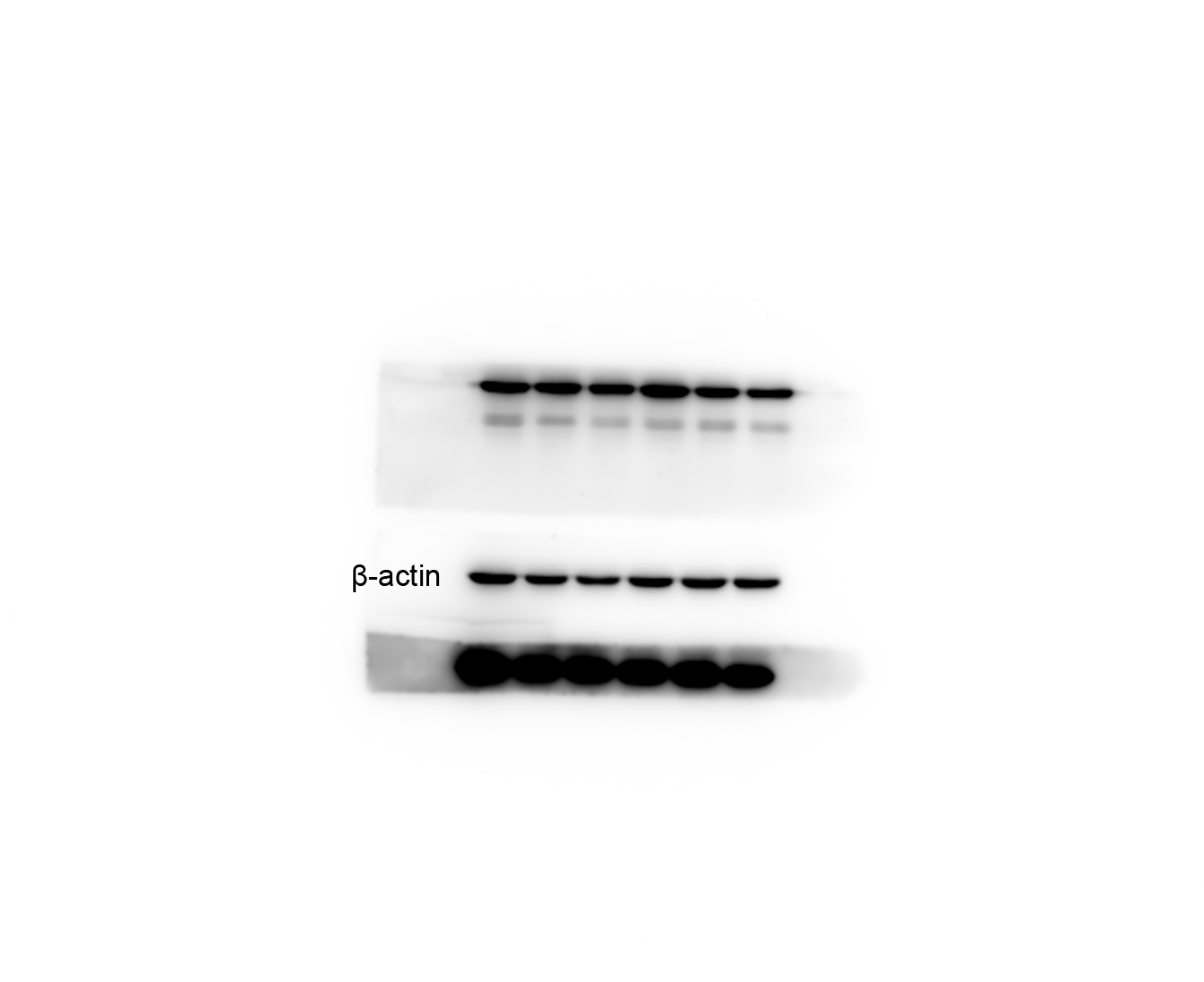

Supplement: Supplementary file 1 [file DataSheet1.ZIP › original WB figures/Figure3/Figure3D-β-actin.tif]

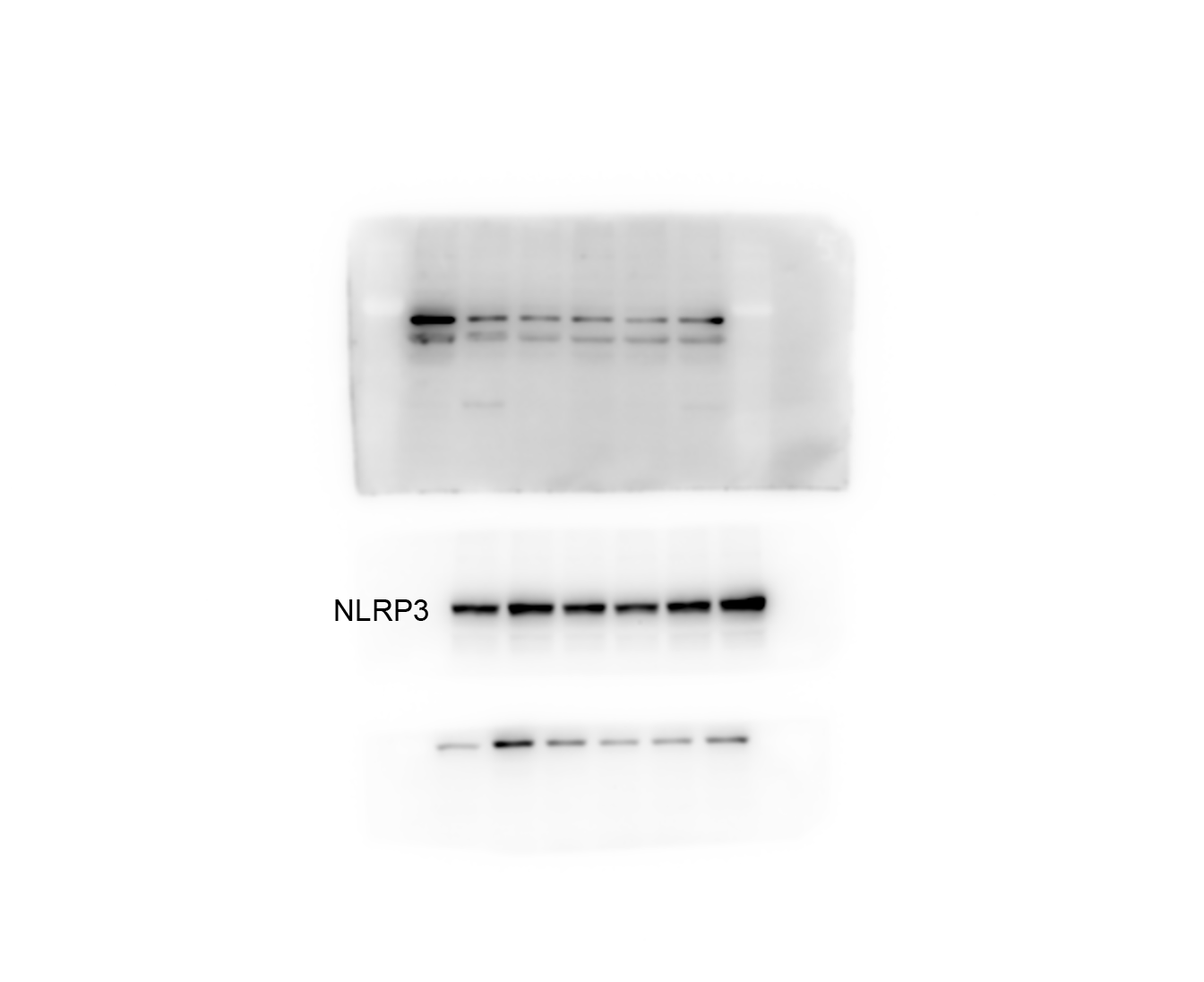

Supplement: Supplementary file 1 [file DataSheet1.ZIP › original WB figures/Figure3/Figure3E-NLRP3.tif]

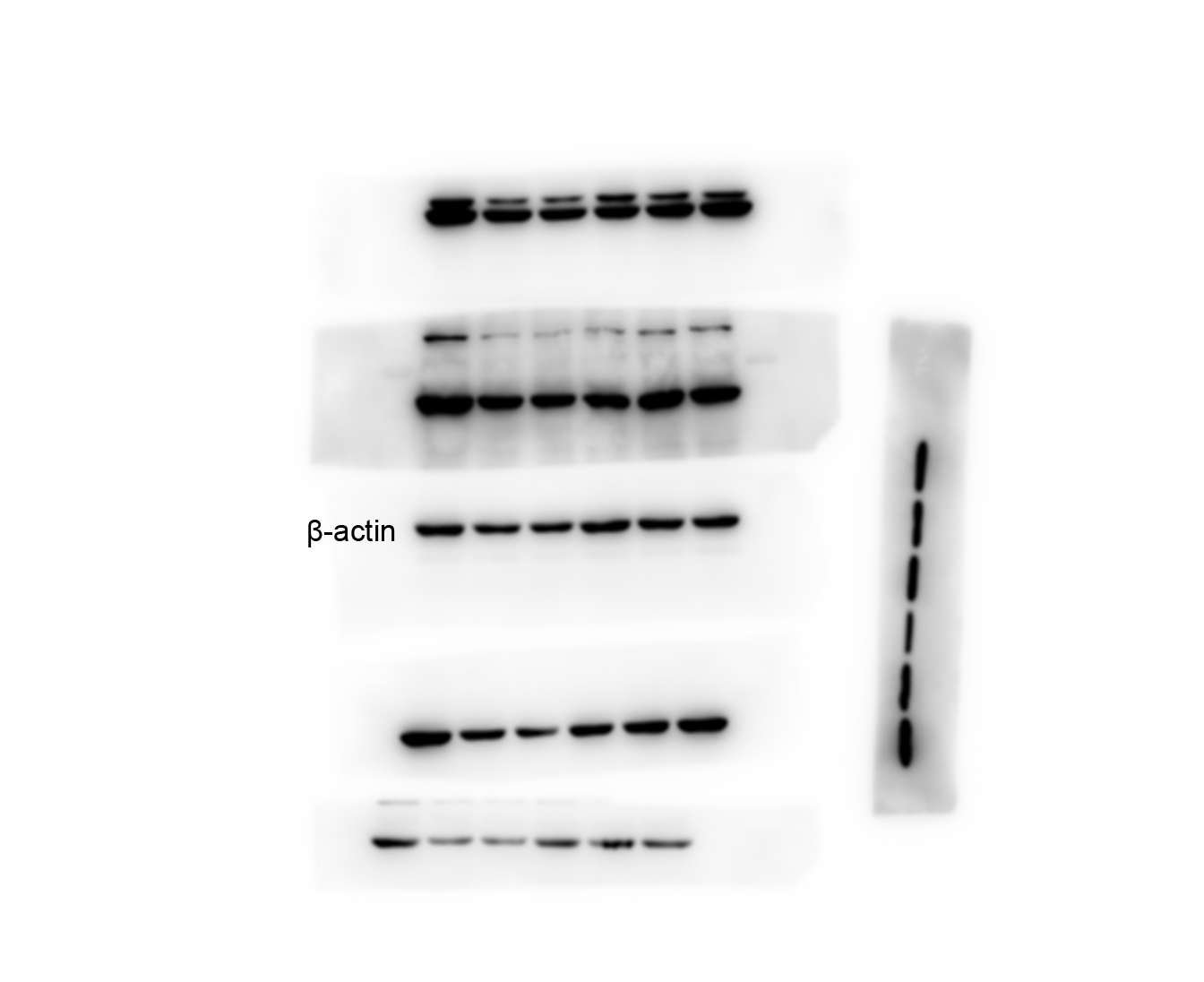

Supplement: Supplementary file 1 [file DataSheet1.ZIP › original WB figures/Figure3/Figure3E-β-actin.tif]

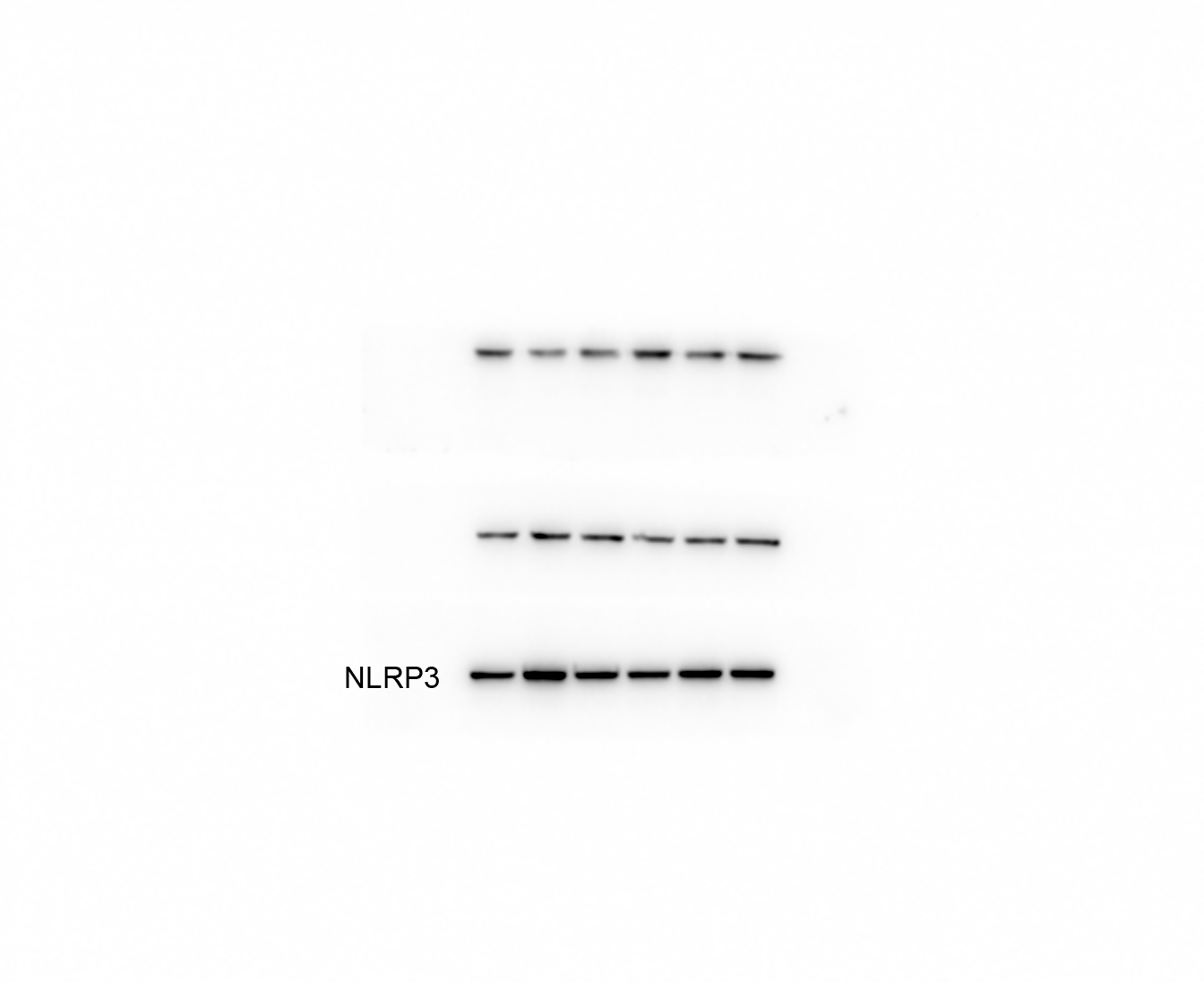

Supplement: Supplementary file 1 [file DataSheet1.ZIP › original WB figures/Figure3/Figure3F-NLRP3.tif]

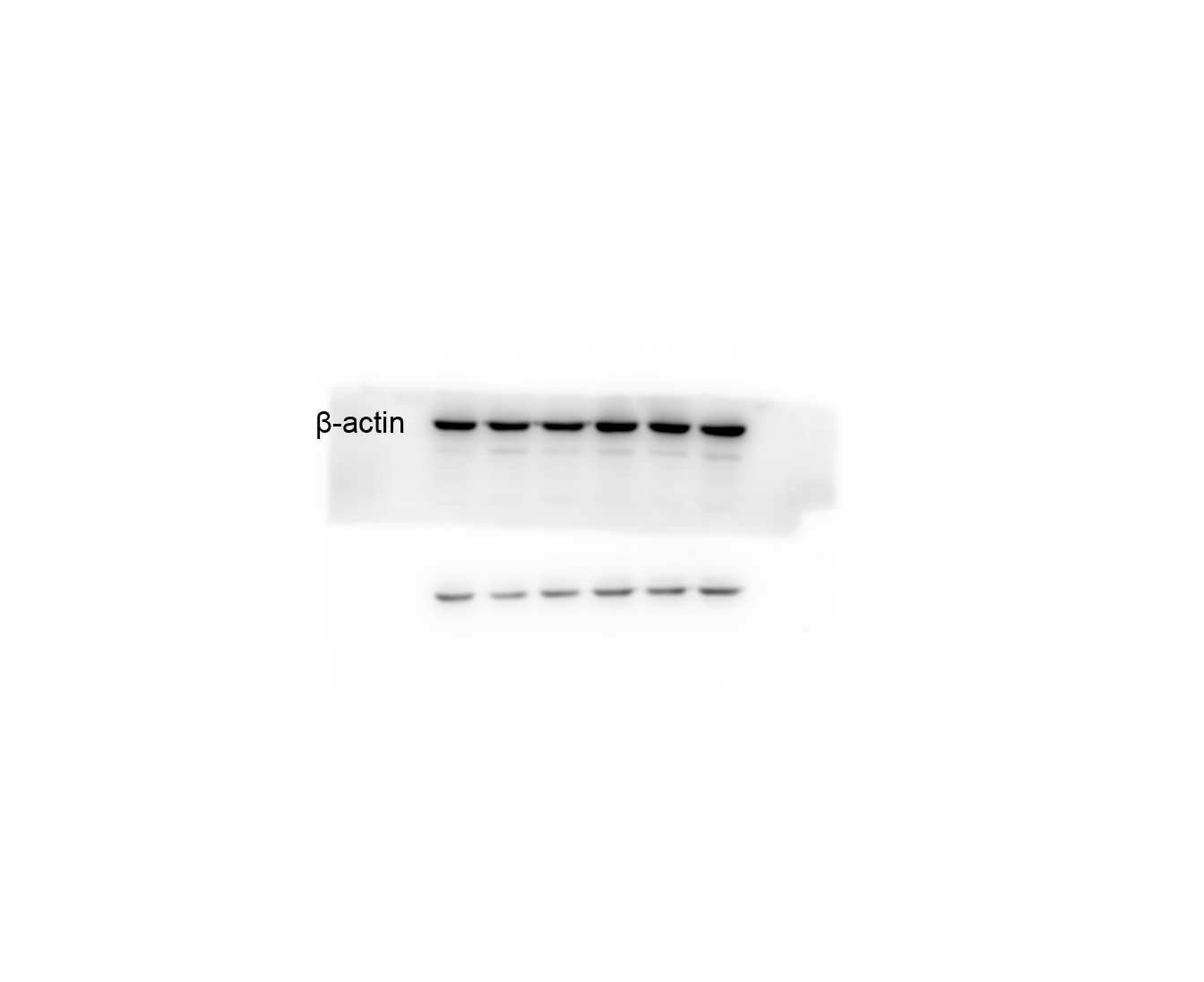

Supplement: Supplementary file 1 [file DataSheet1.ZIP › original WB figures/Figure3/Figure3F-β-actin.tif]

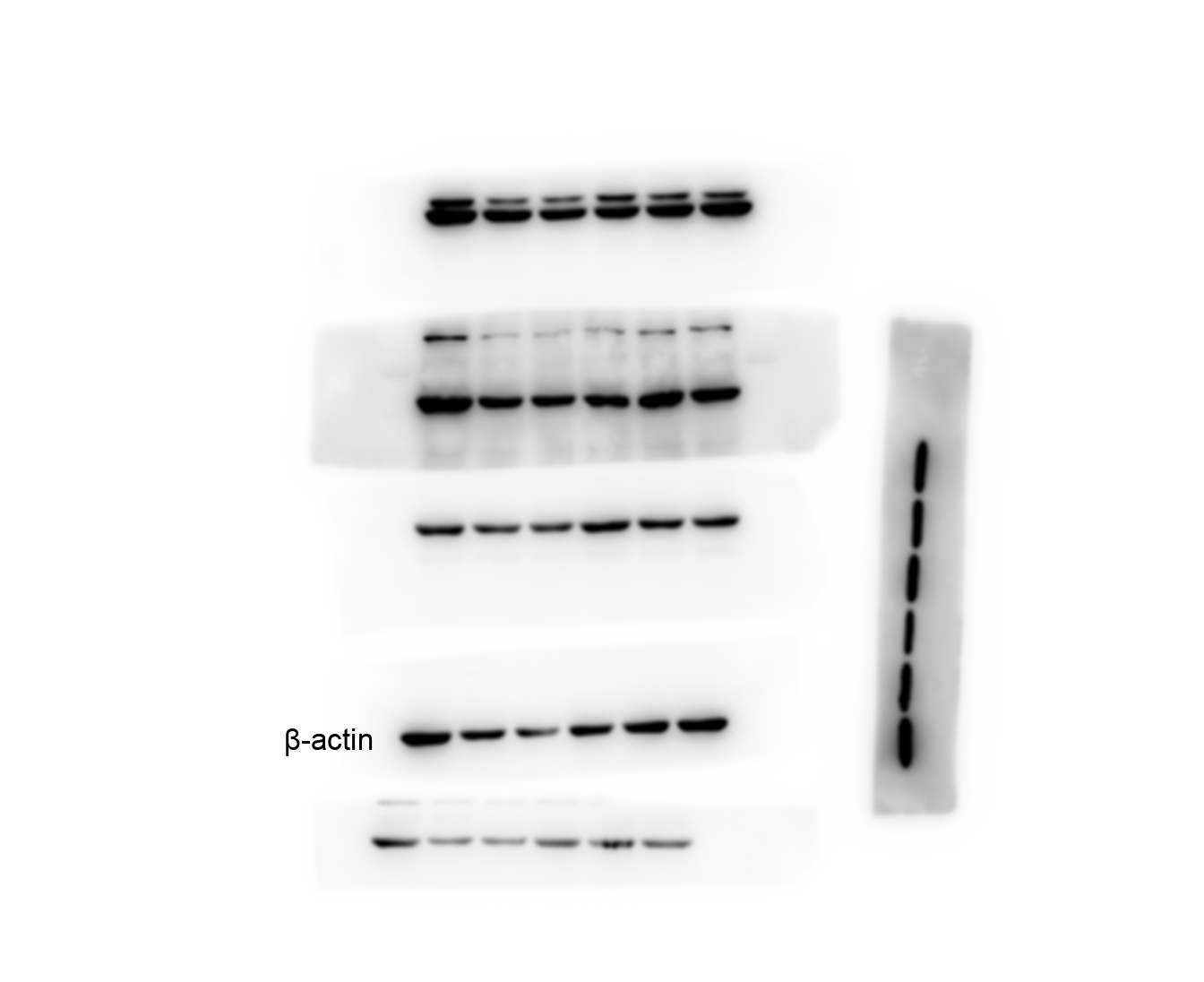

Supplement: Supplementary file 1 [file DataSheet1.ZIP › original WB figures/Figure4/Figure4C-actin.tif]

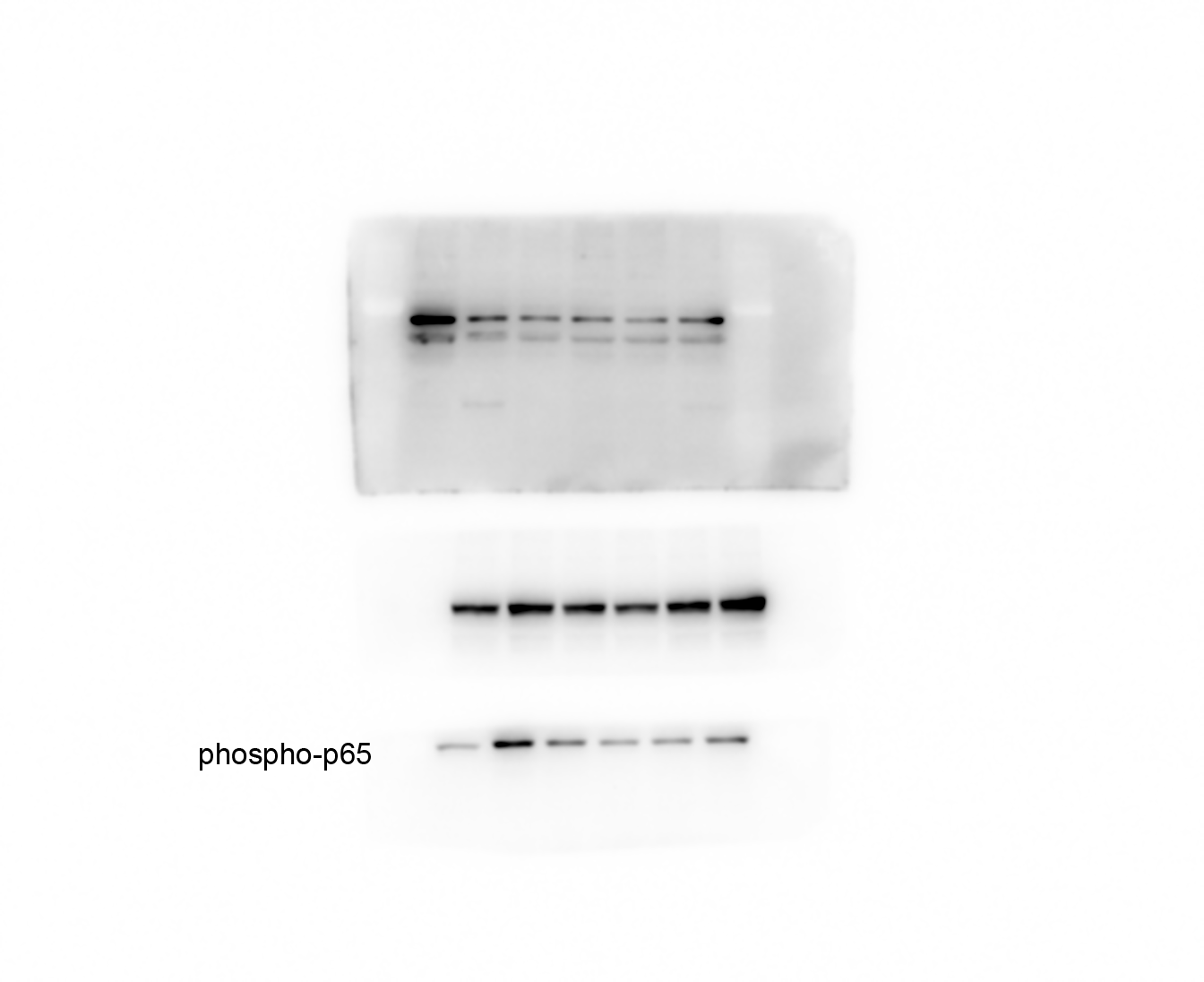

Supplement: Supplementary file 1 [file DataSheet1.ZIP › original WB figures/Figure4/Figure4C-P-p65-30s.tif]

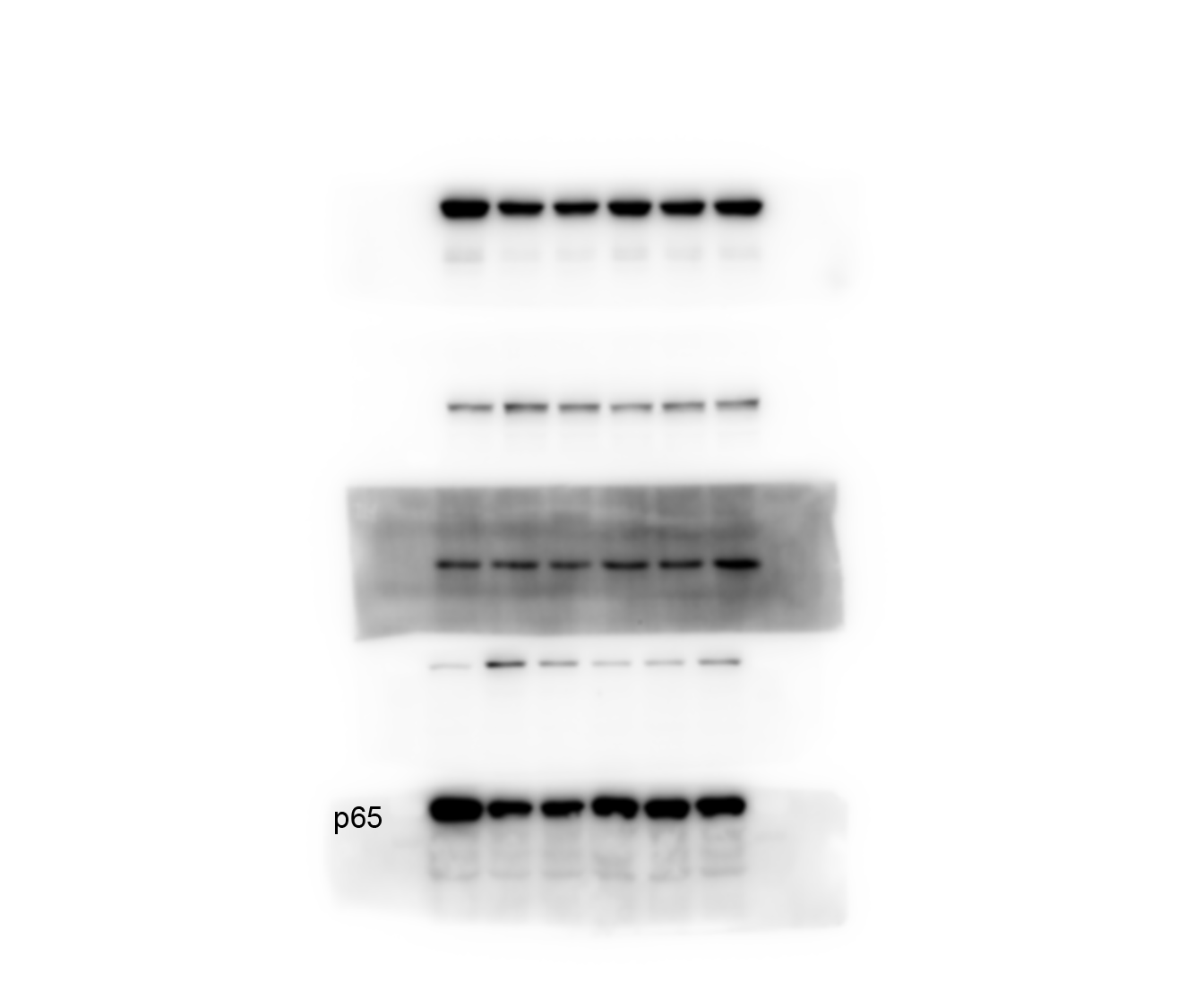

Supplement: Supplementary file 1 [file DataSheet1.ZIP › original WB figures/Figure4/Figure4D-p65.tif]

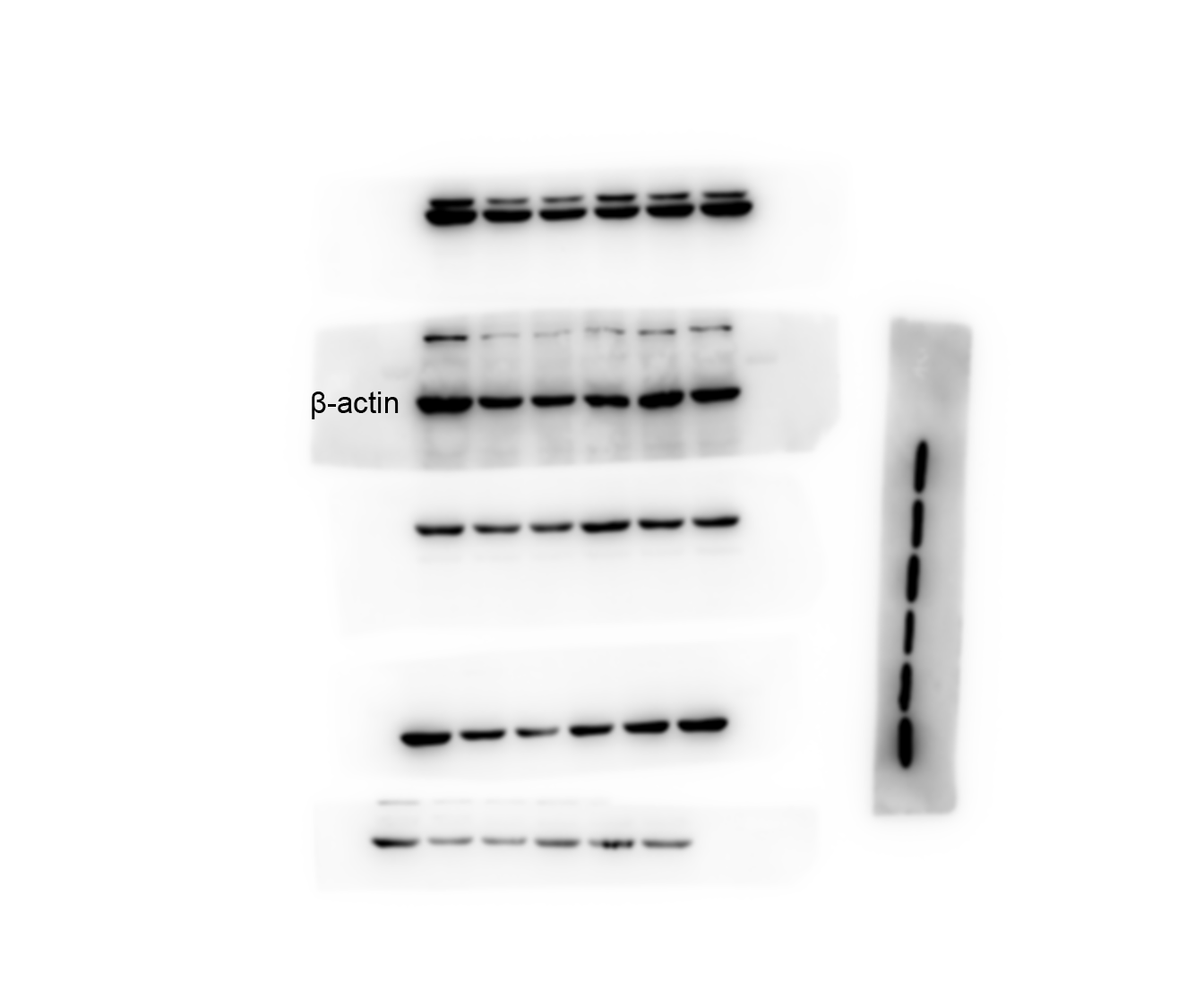

Supplement: Supplementary file 1 [file DataSheet1.ZIP › original WB figures/Figure4/Figure4D-β-actin.tif]

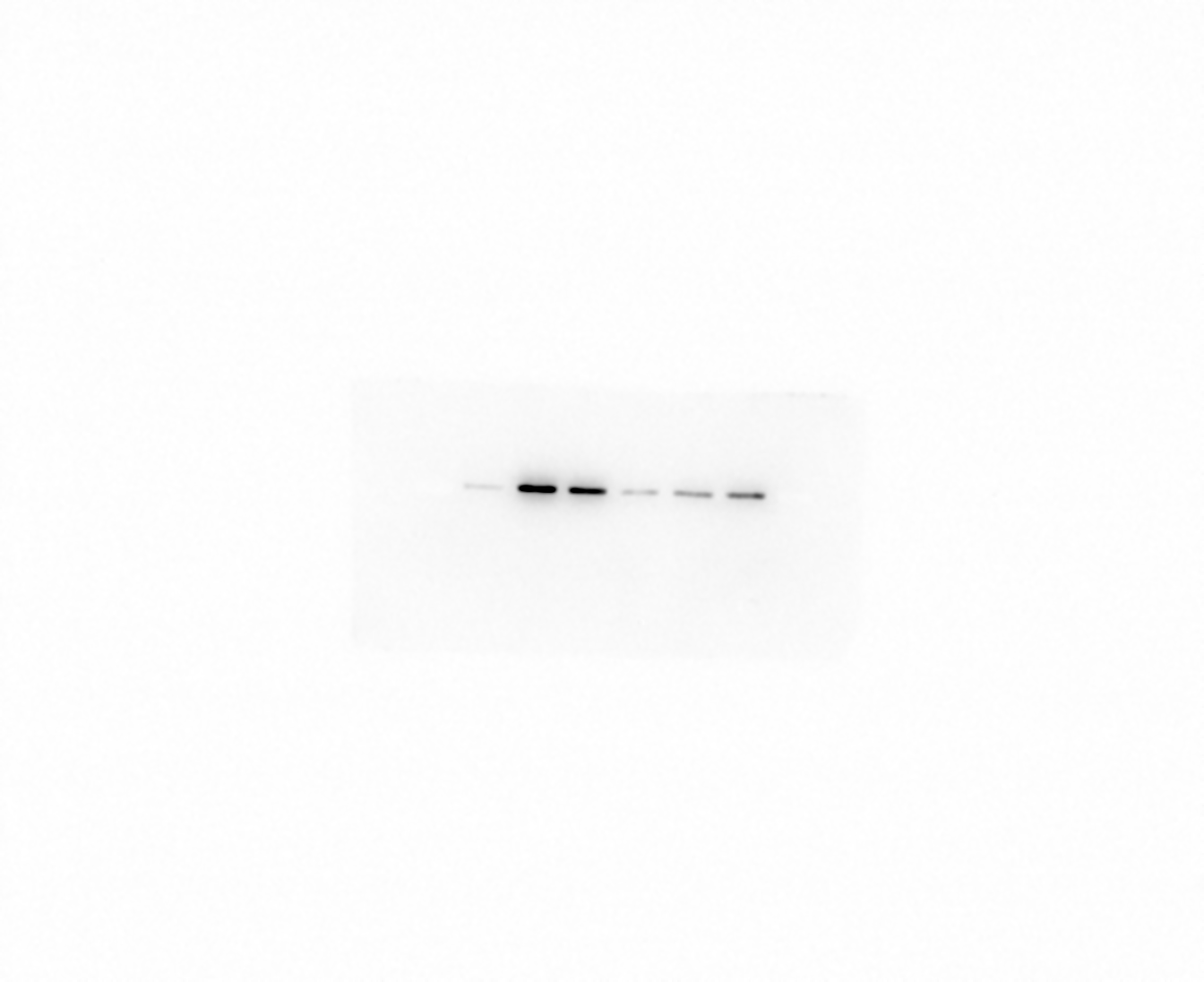

Supplement: Supplementary file 1 [file DataSheet1.ZIP › original WB figures/Figure4/Figure4E-p-p65.tif]

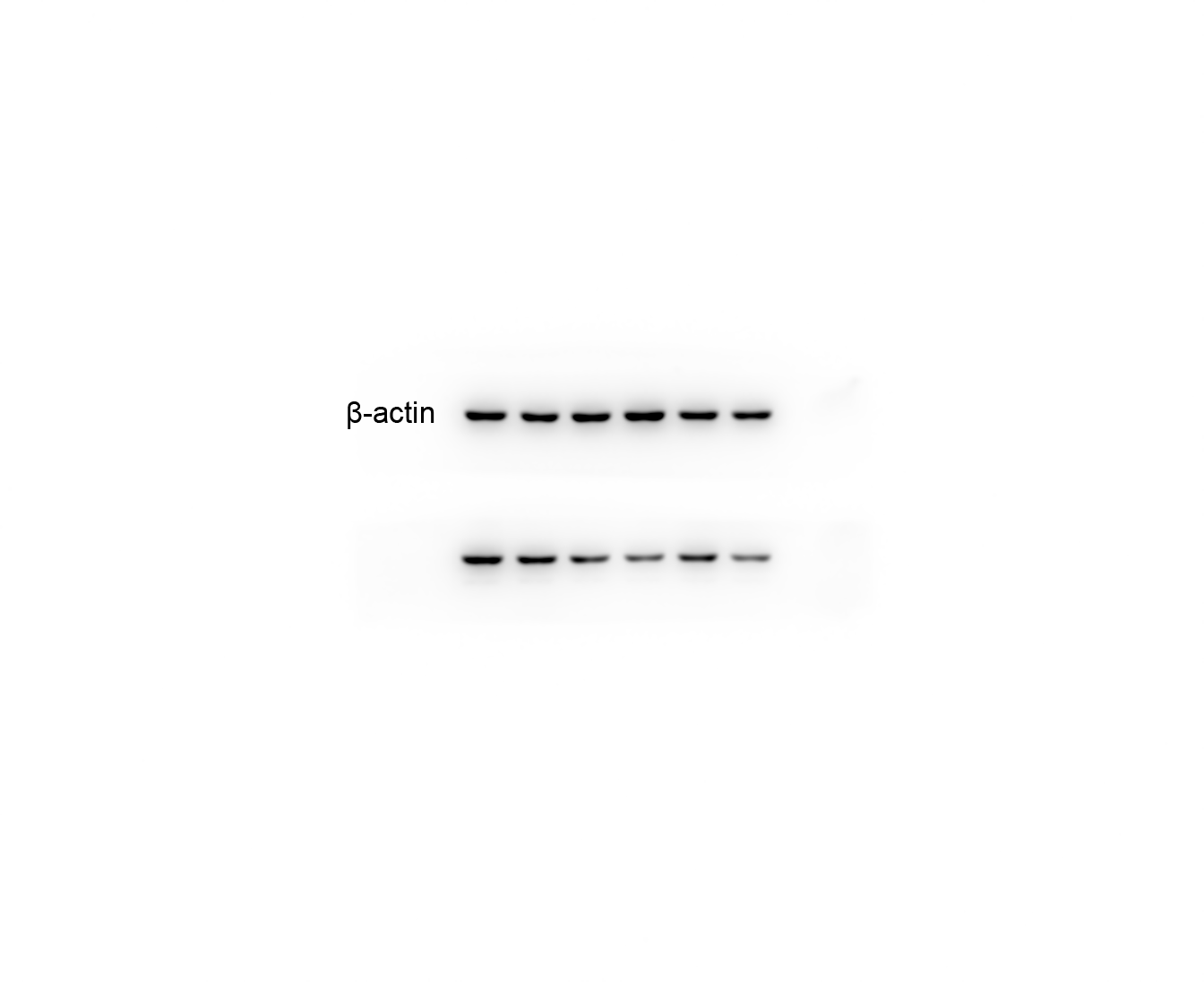

Supplement: Supplementary file 1 [file DataSheet1.ZIP › original WB figures/Figure4/Figure4E-β-actin.tif]

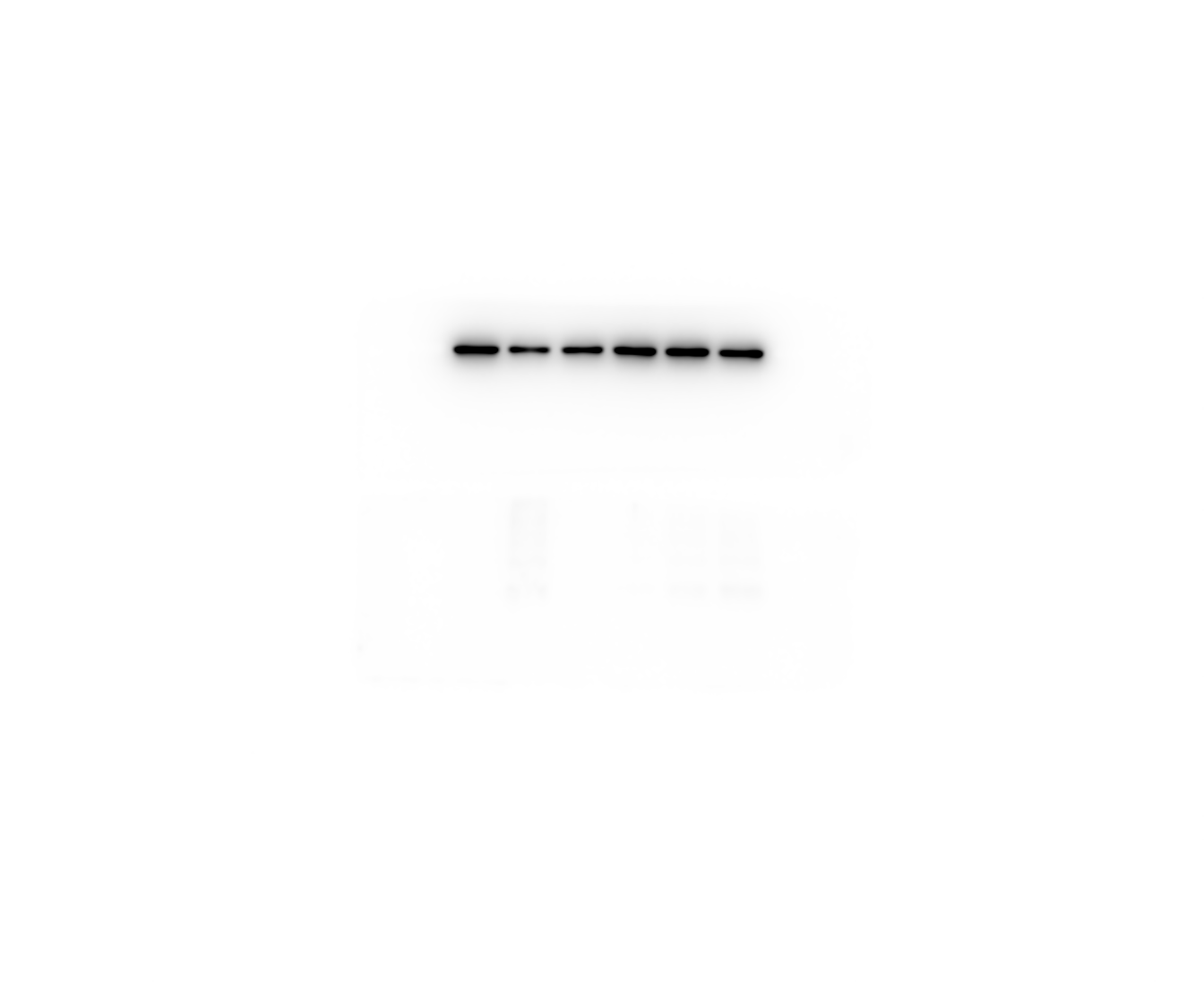

Supplement: Supplementary file 1 [file DataSheet1.ZIP › original WB figures/Figure4/Figure4F-p65.tif]

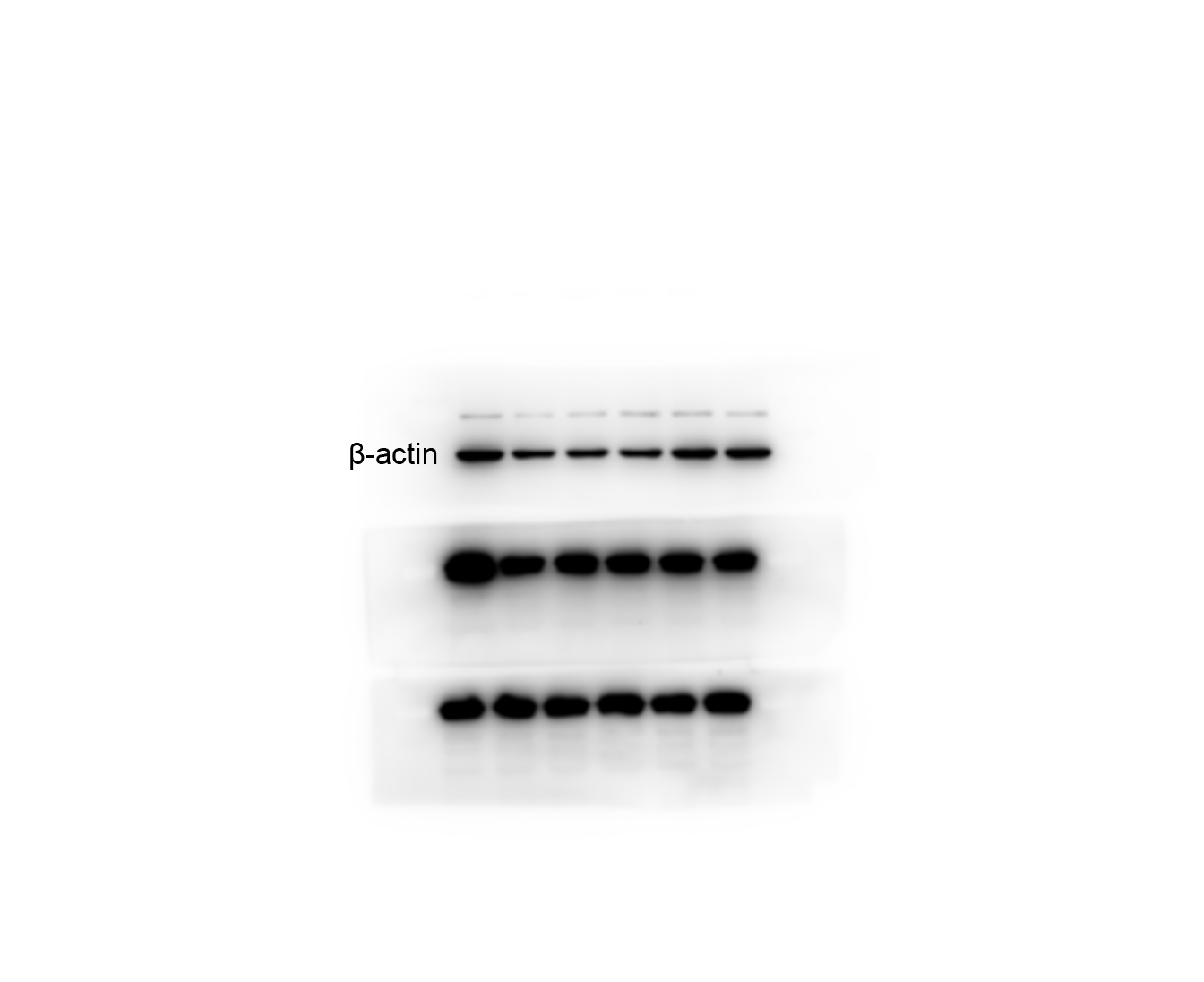

Supplement: Supplementary file 1 [file DataSheet1.ZIP › original WB figures/Figure4/Figure4F-β-actin.tif]

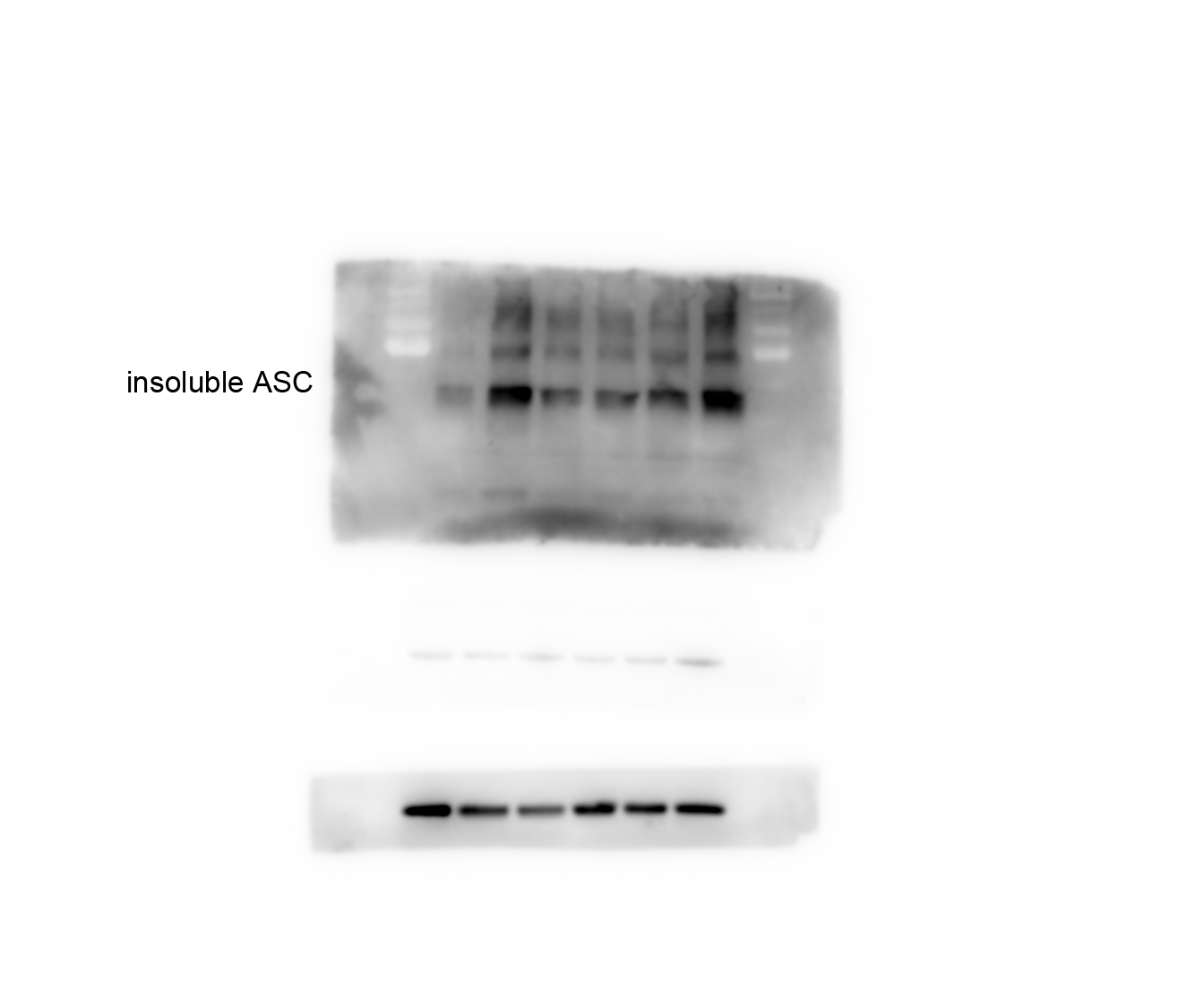

Supplement: Supplementary file 1 [file DataSheet1.ZIP › original WB figures/Figure5/Figure5C-insoluble ASC.tif]

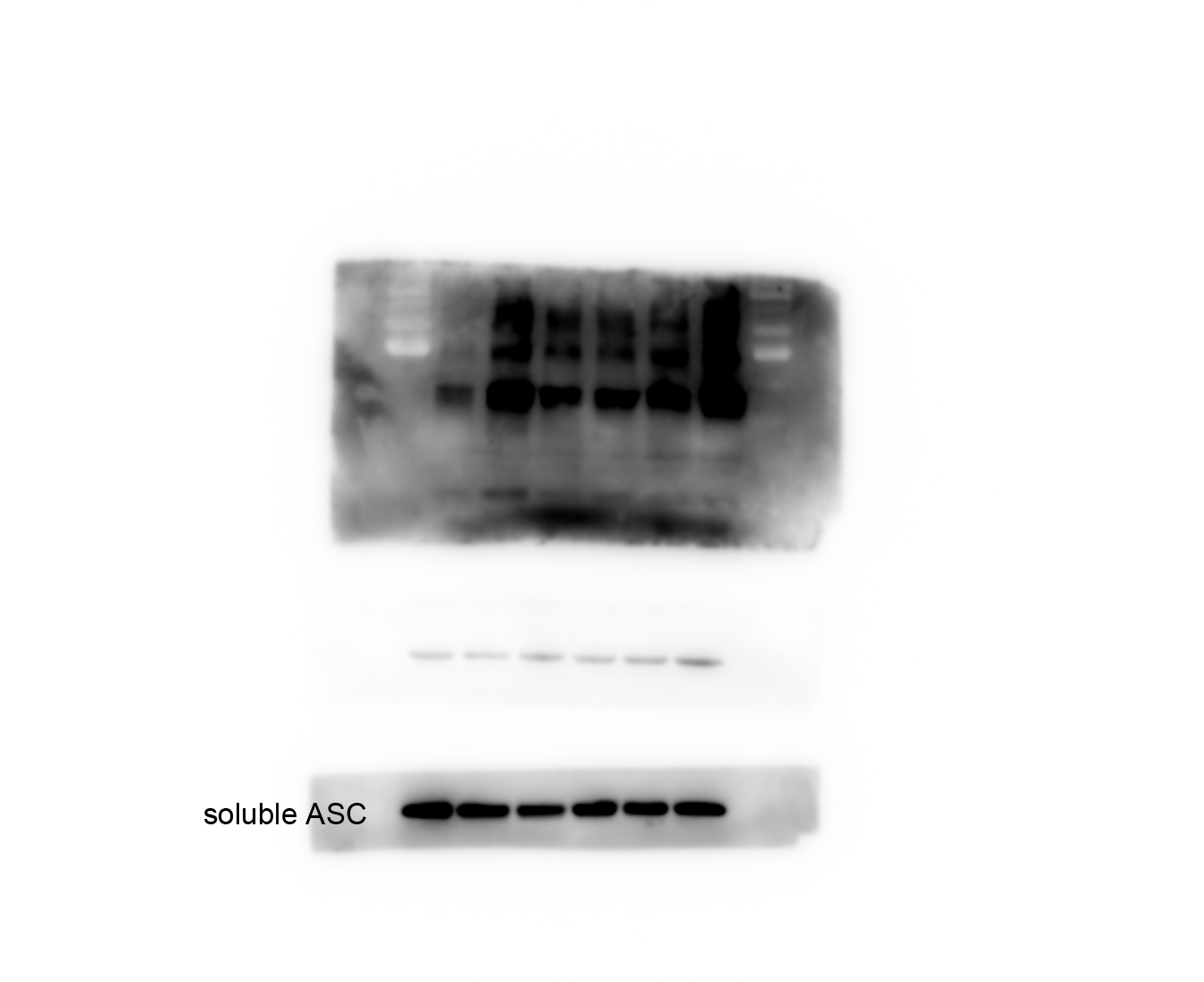

Supplement: Supplementary file 1 [file DataSheet1.ZIP › original WB figures/Figure5/Figure5C-soluble ASC.tif]

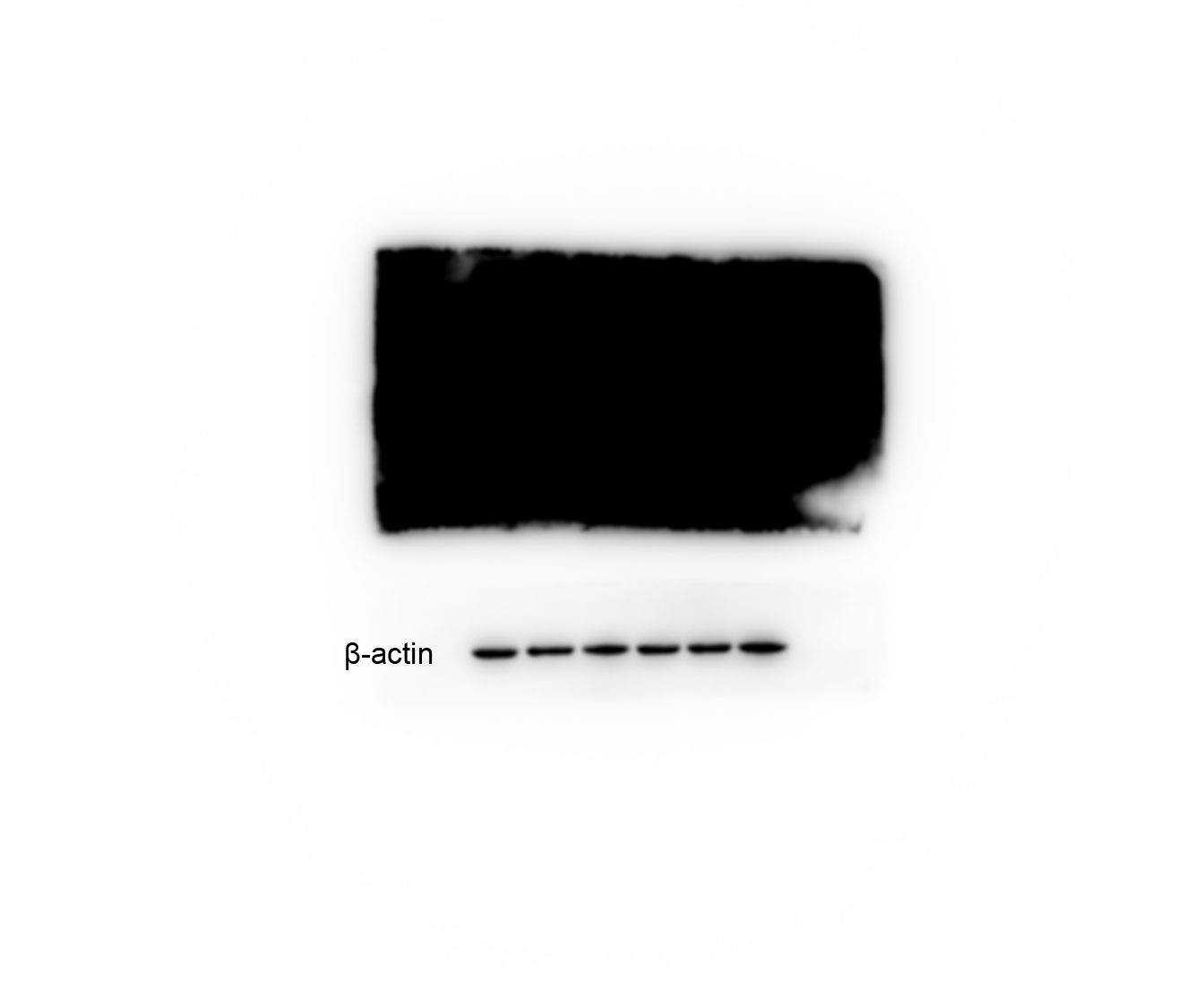

Supplement: Supplementary file 1 [file DataSheet1.ZIP › original WB figures/Figure5/Figure5C-β-actin.tif]

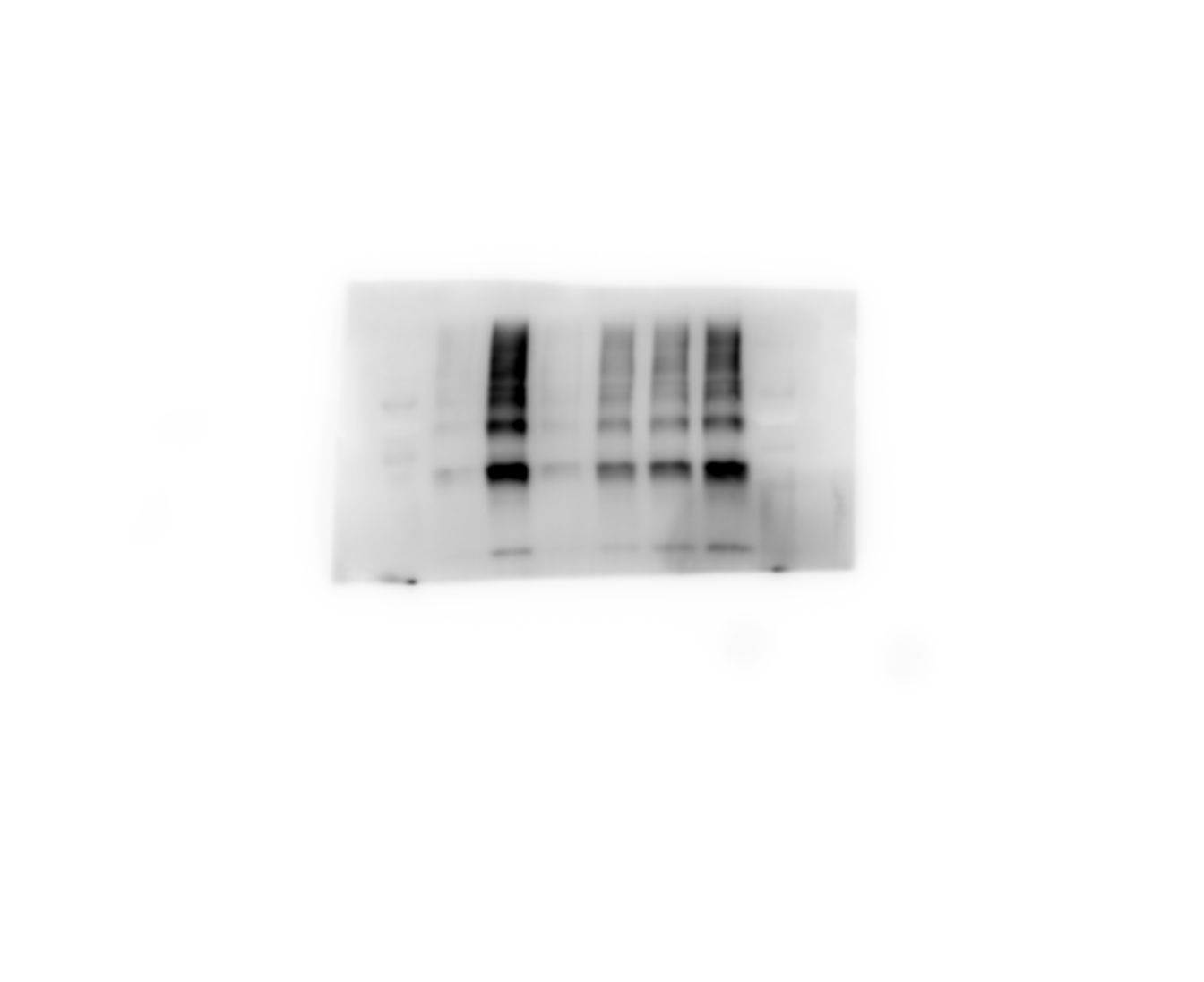

Supplement: Supplementary file 1 [file DataSheet1.ZIP › original WB figures/Figure5/Figure5D-insoluble ASC.tif]

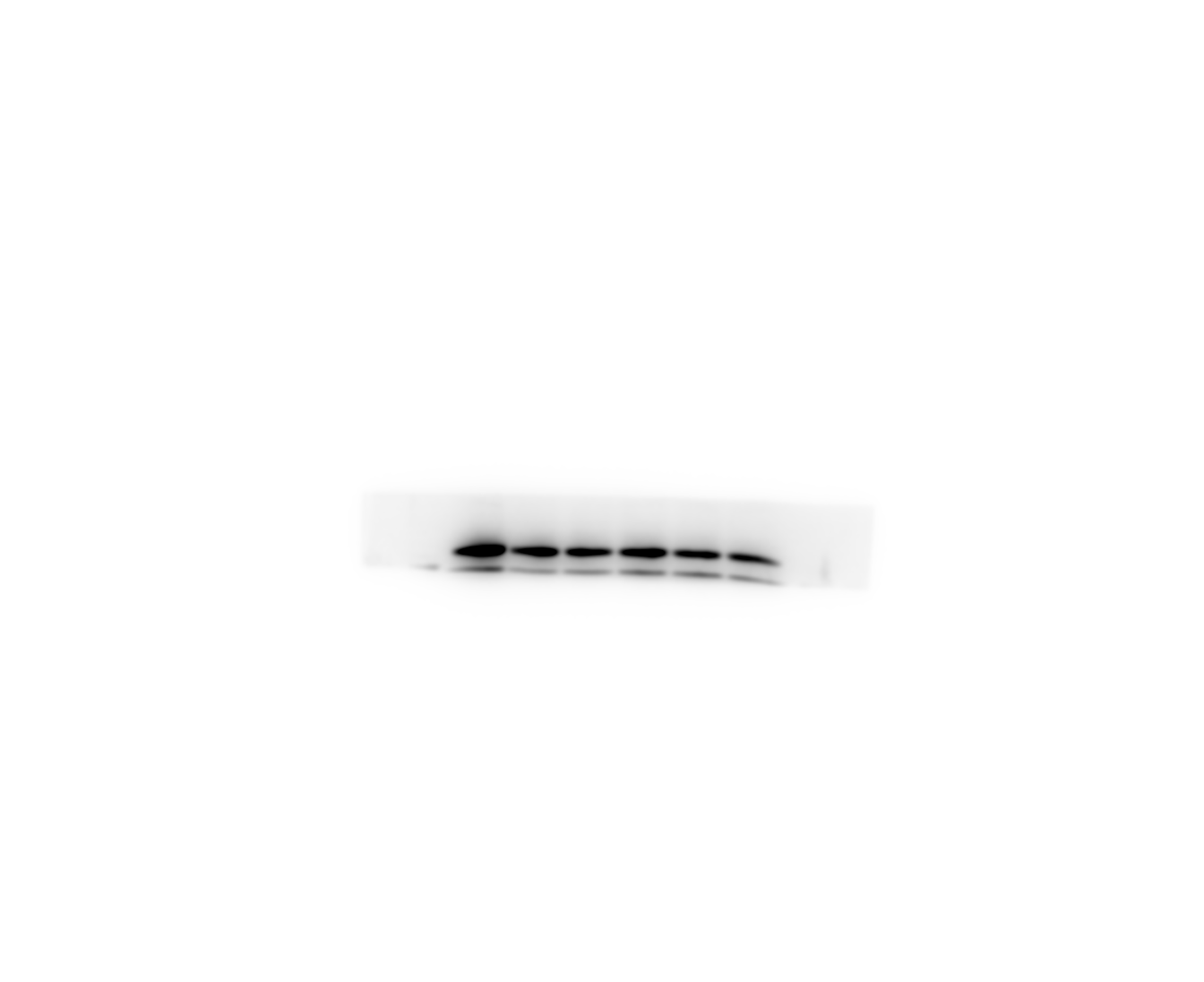

Supplement: Supplementary file 1 [file DataSheet1.ZIP › original WB figures/Figure5/Figure5D-soluble ASC.tif]

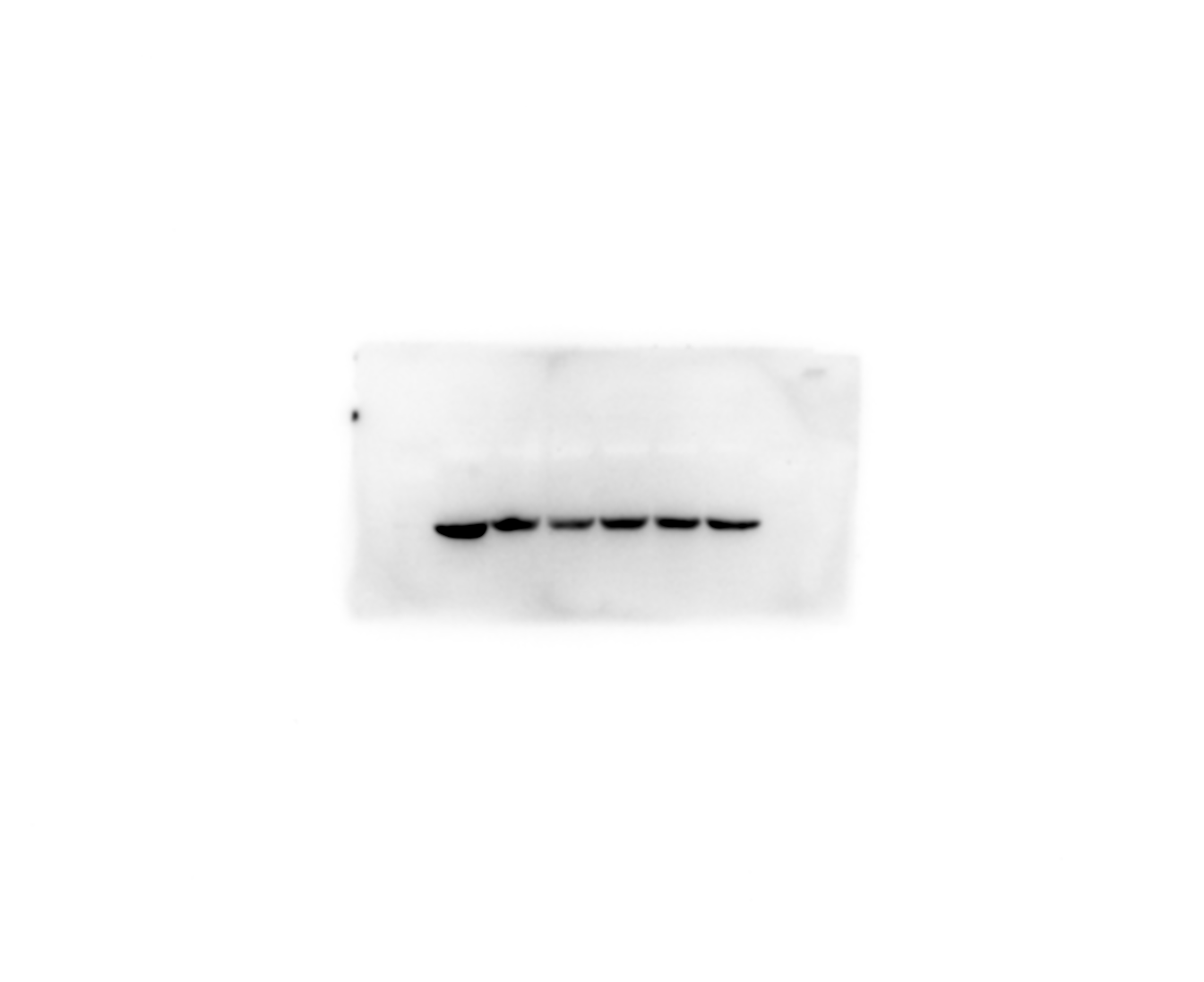

Supplement: Supplementary file 1 [file DataSheet1.ZIP › original WB figures/Figure5/Figure5D-β-actin.tif]

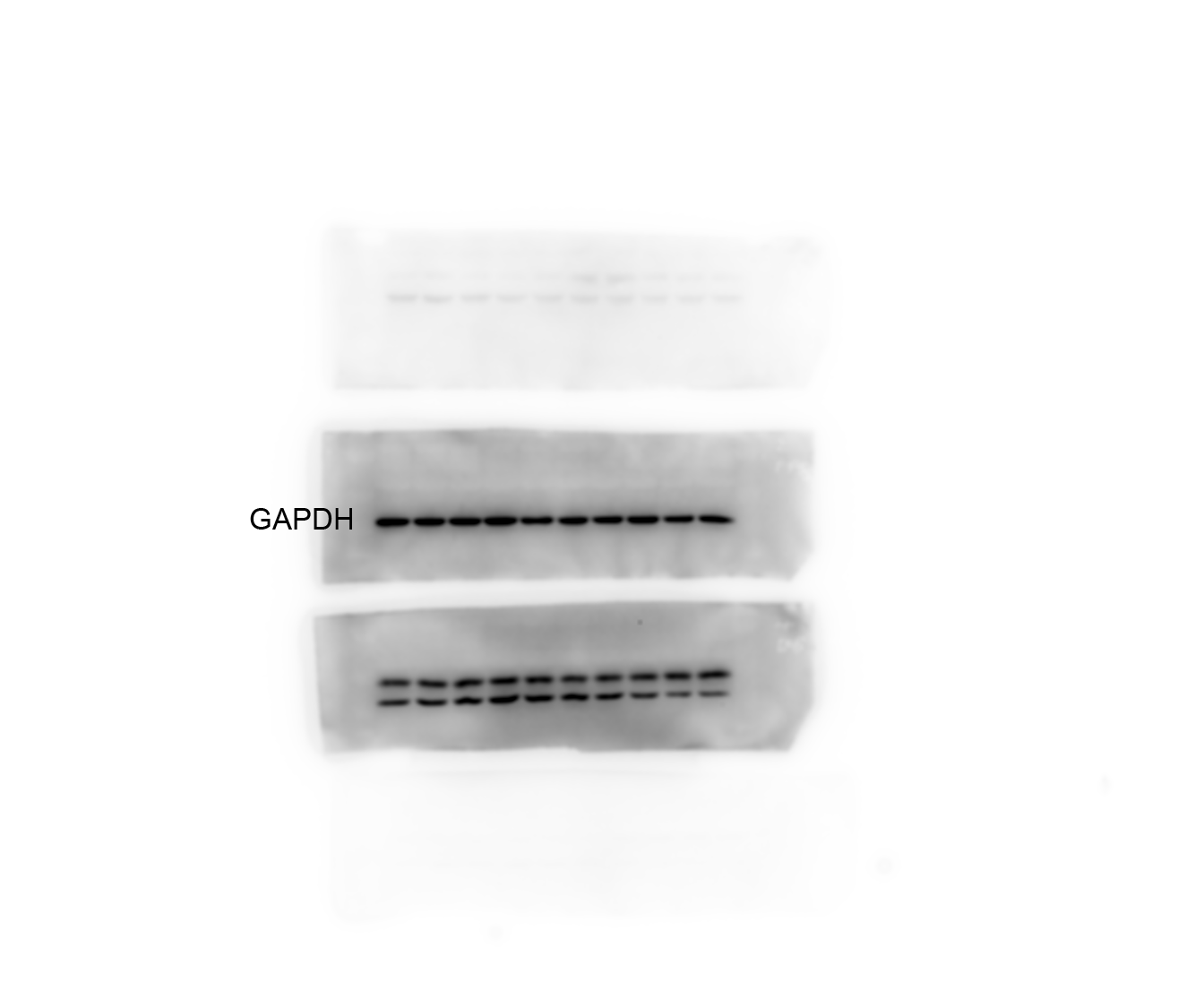

Supplement: Supplementary file 1 [file DataSheet1.ZIP › original WB figures/Figure6/Figure6A-GAPDH.tif]

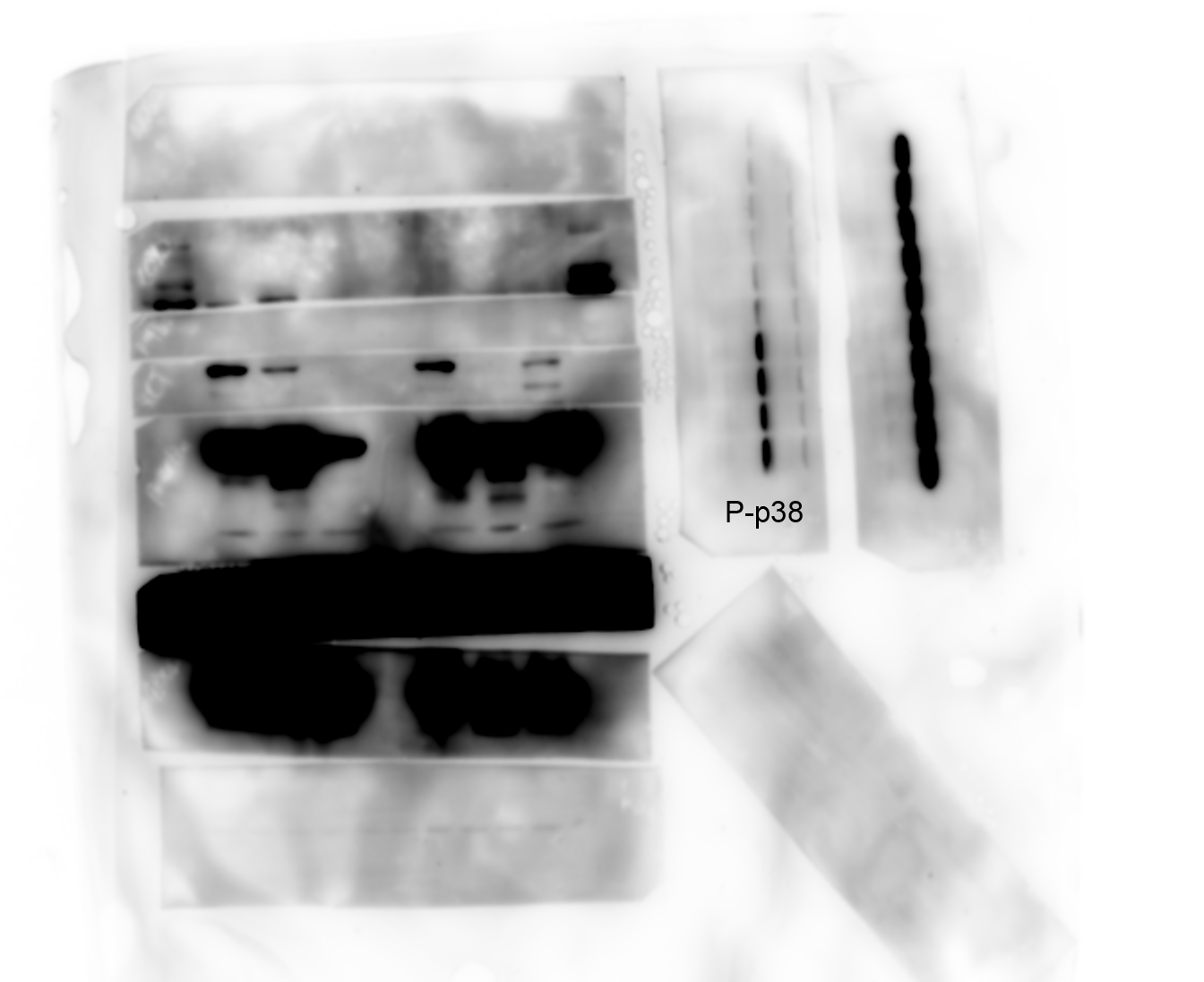

Supplement: Supplementary file 1 [file DataSheet1.ZIP › original WB figures/Figure6/Figure6A-P-p38.tif]

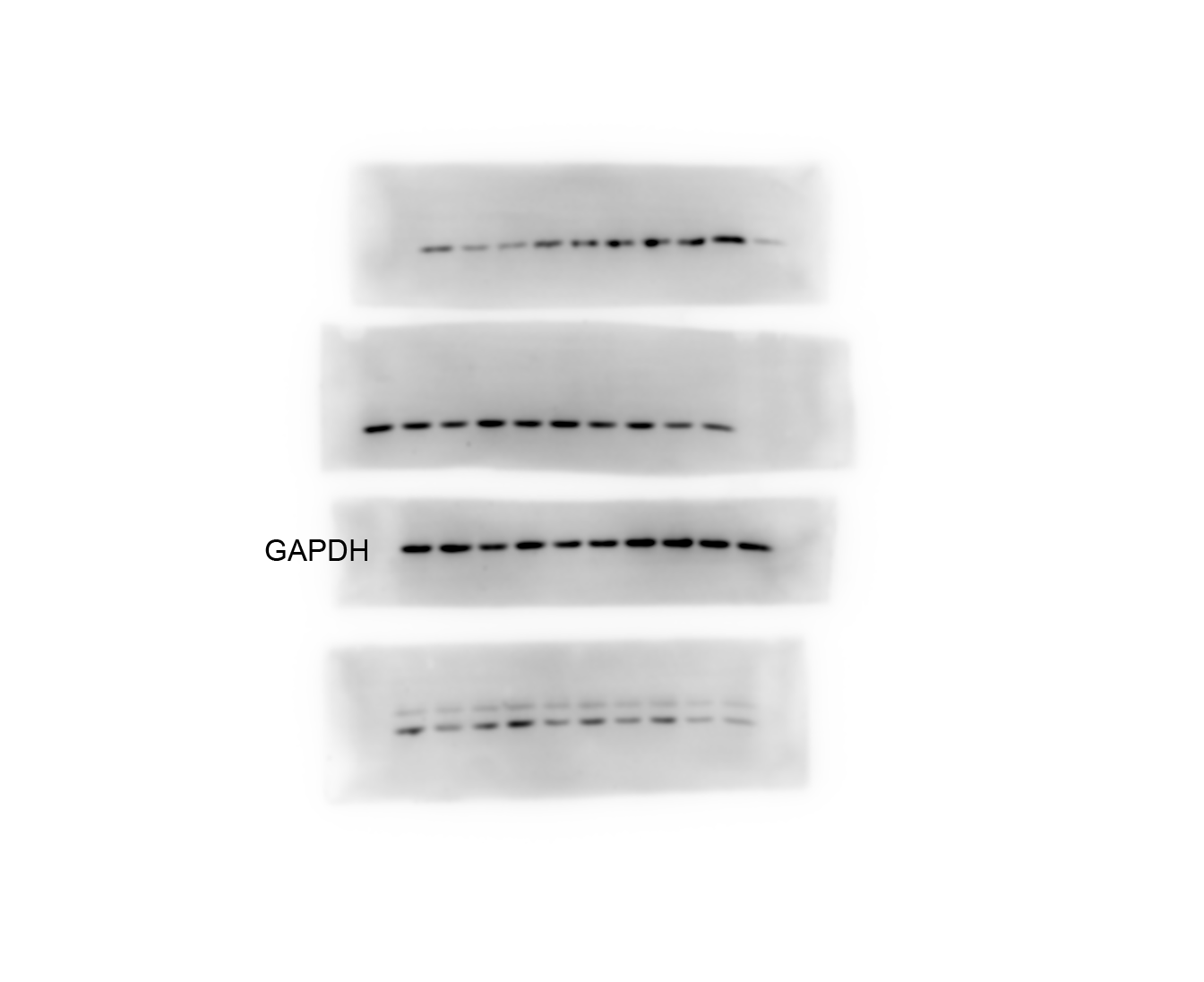

Supplement: Supplementary file 1 [file DataSheet1.ZIP › original WB figures/Figure6/Figure6B-GAPDH.tif]

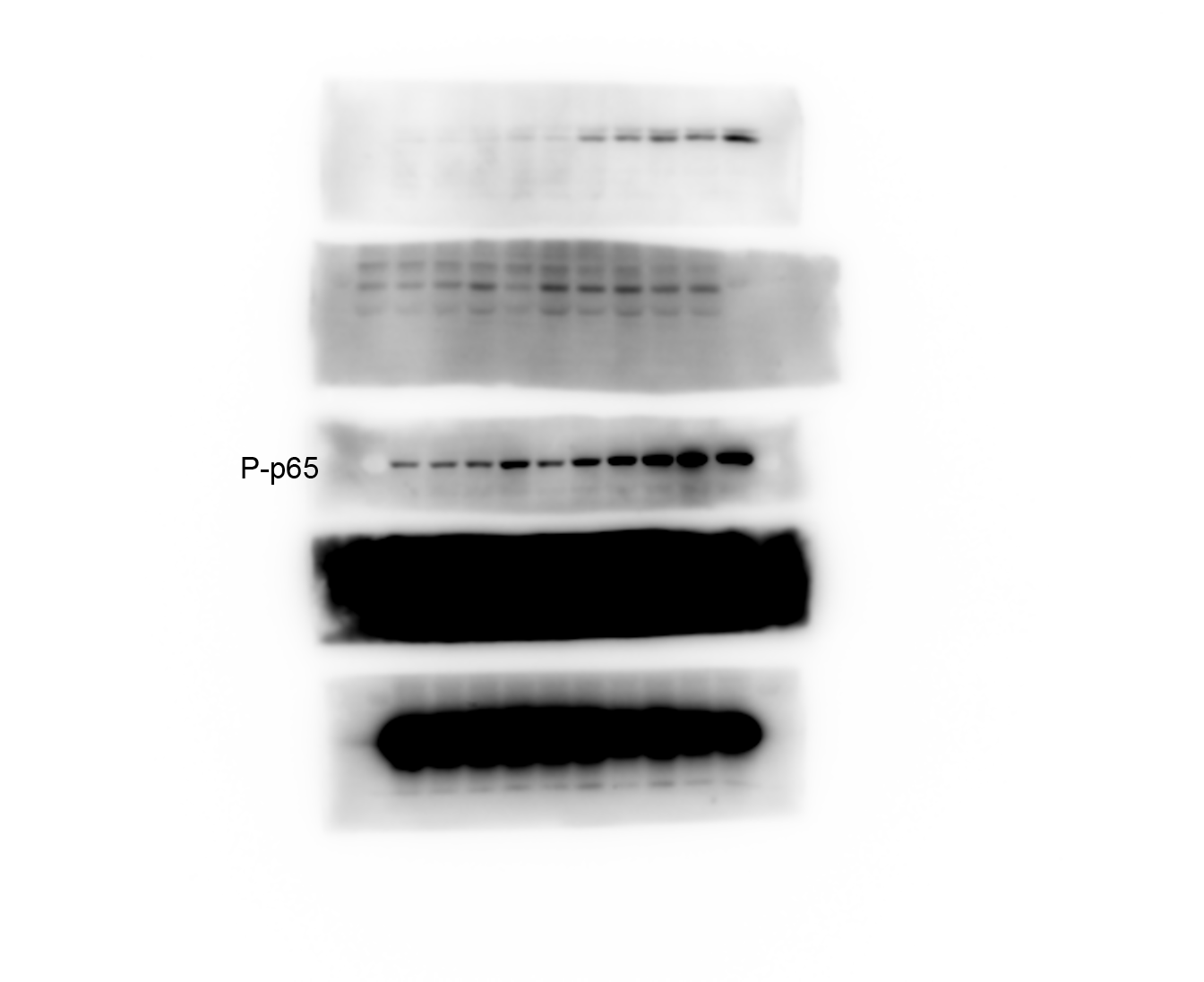

Supplement: Supplementary file 1 [file DataSheet1.ZIP › original WB figures/Figure6/Figure6B-P-p65.tif]

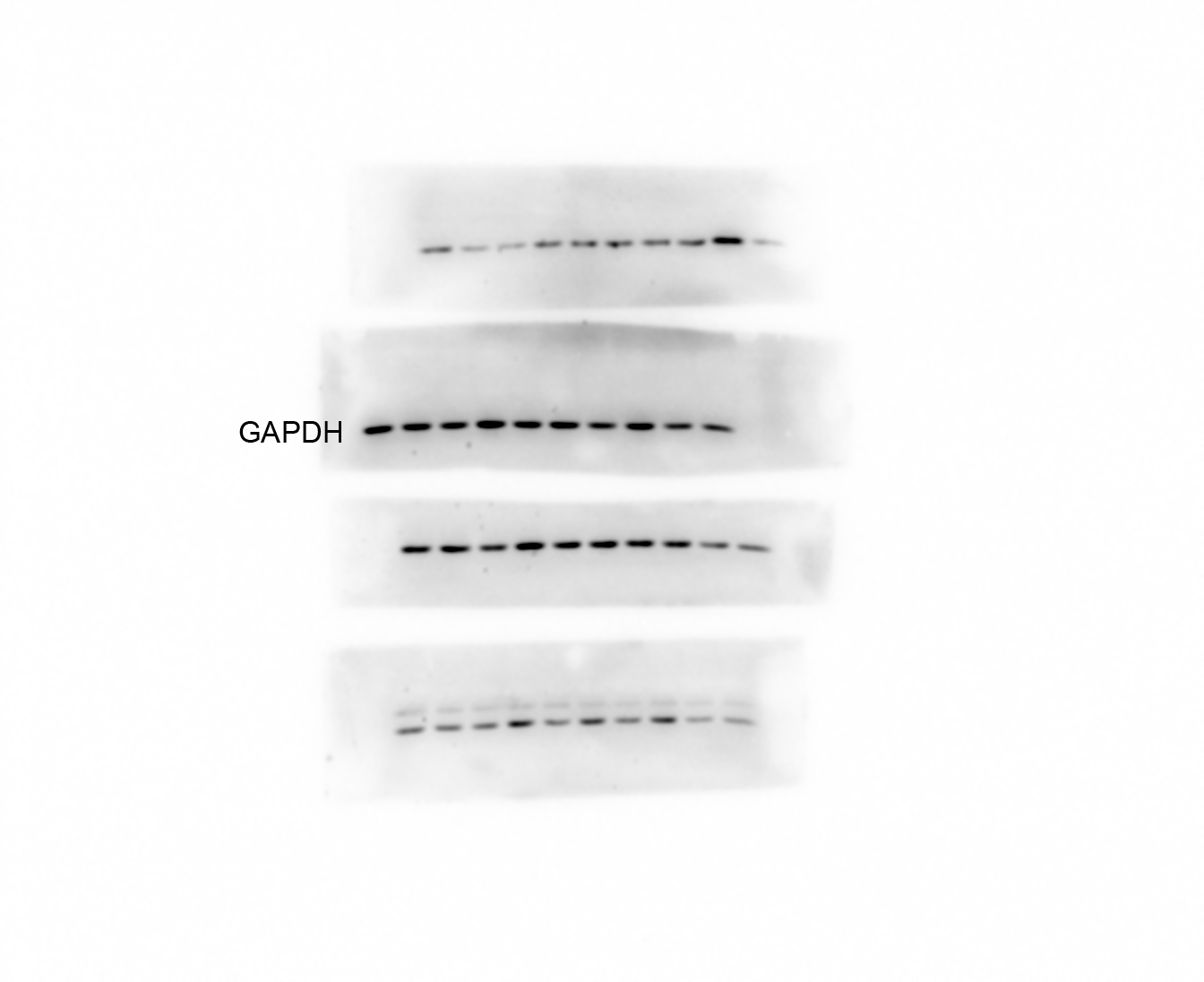

Supplement: Supplementary file 1 [file DataSheet1.ZIP › original WB figures/Figure6/Figure6C-GAPDH.tif]

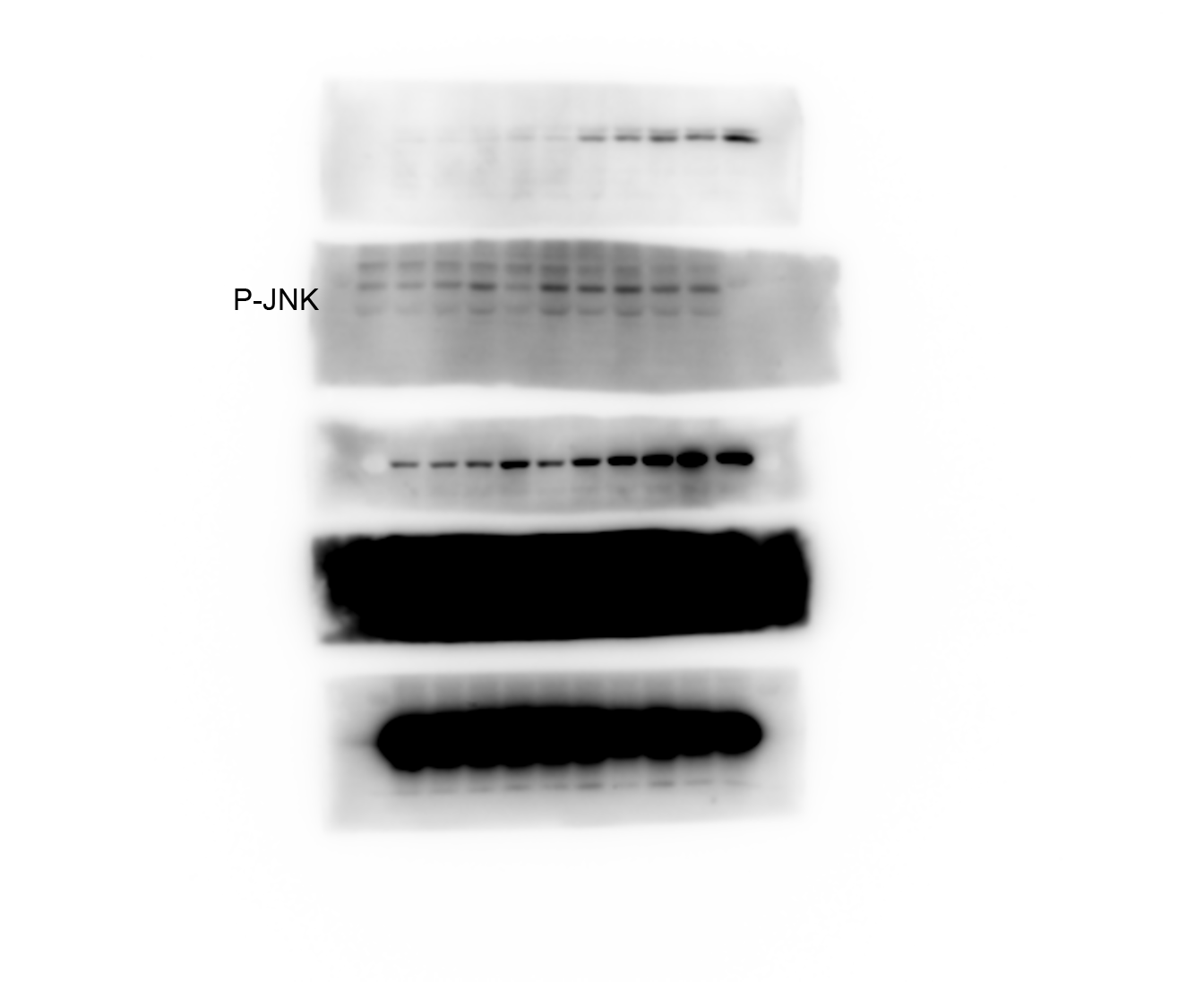

Supplement: Supplementary file 1 [file DataSheet1.ZIP › original WB figures/Figure6/Figure6C-P-JNK.tif]

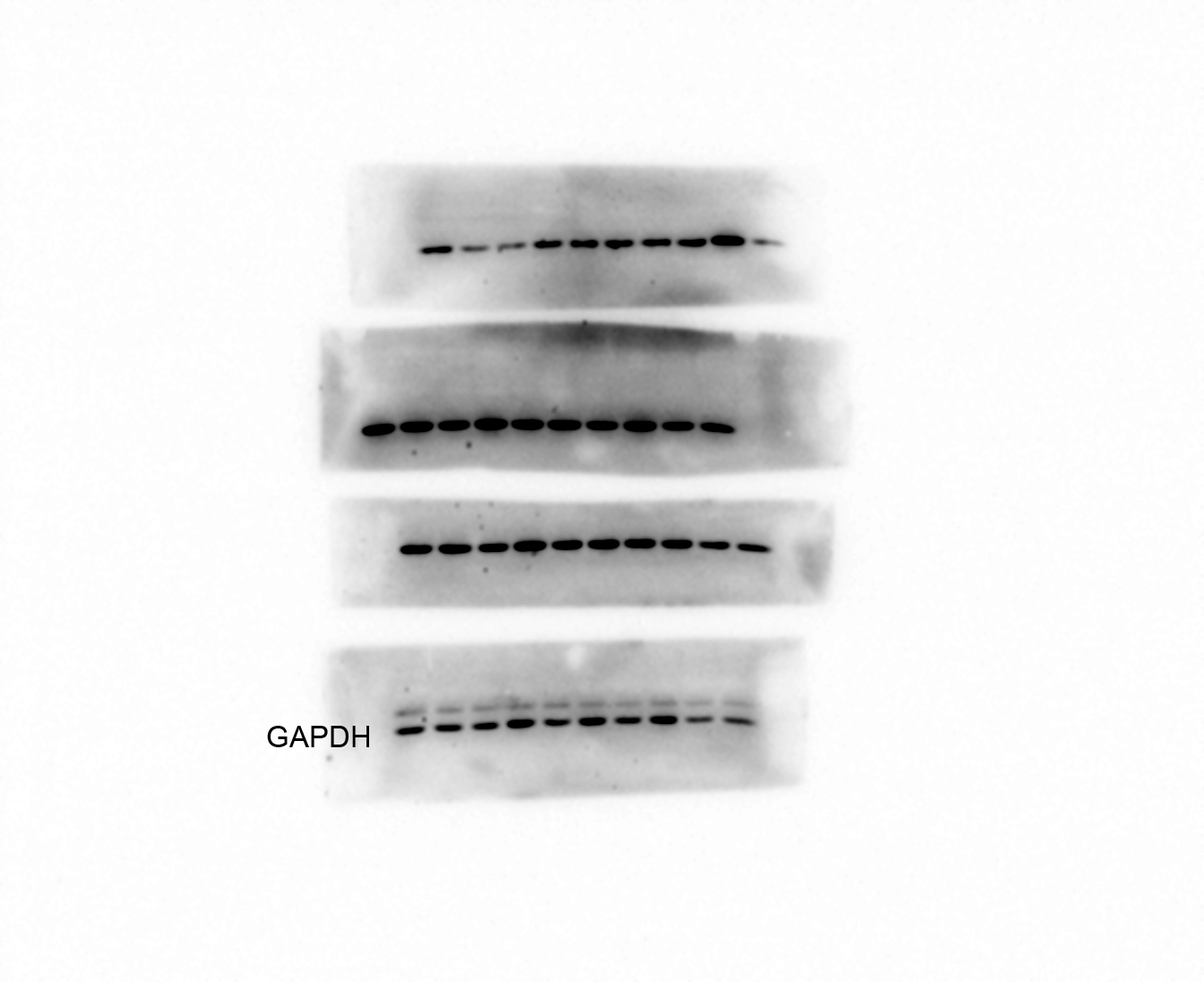

Supplement: Supplementary file 1 [file DataSheet1.ZIP › original WB figures/Figure6/Figure6D-GAPDH.tif]

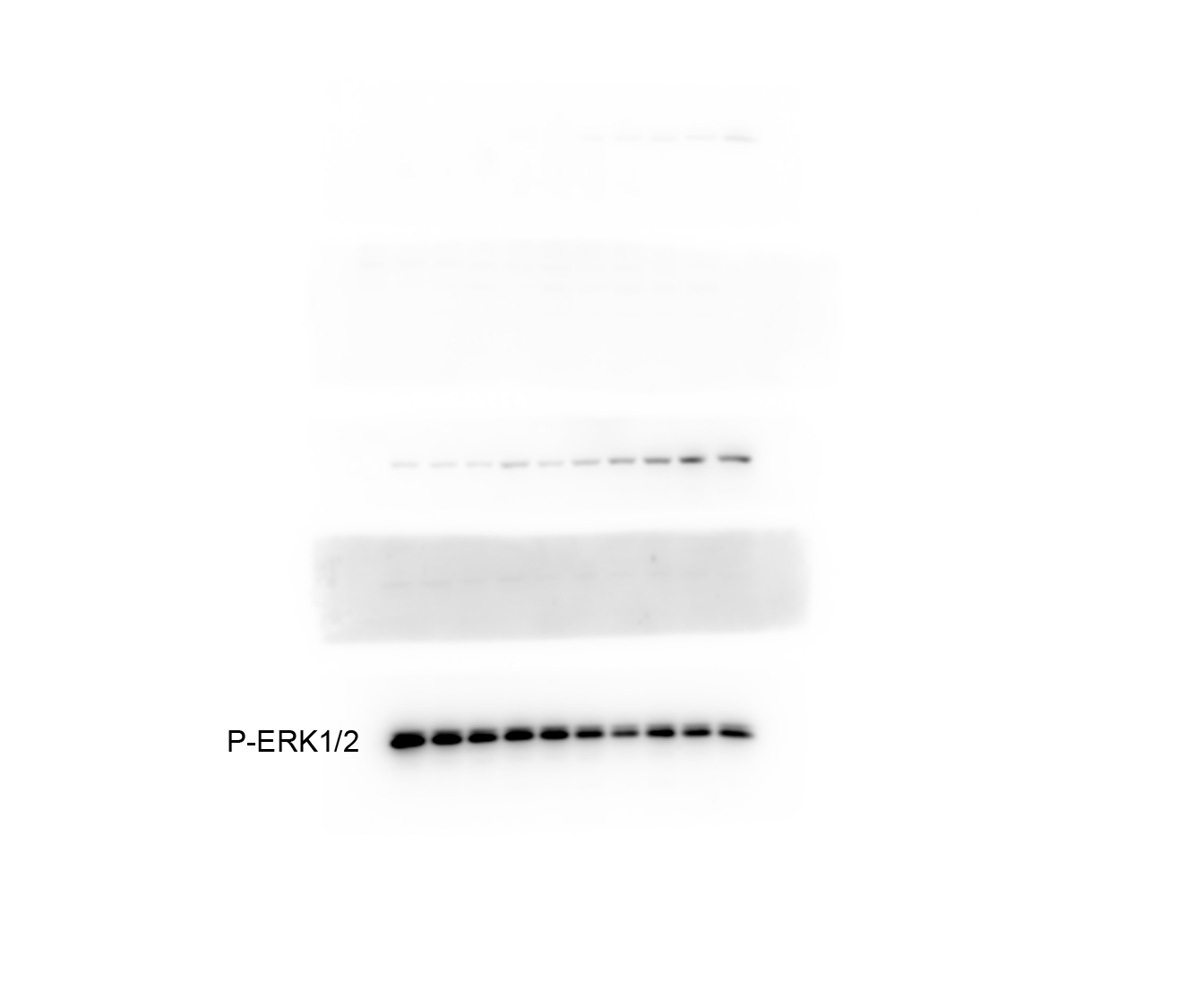

Supplement: Supplementary file 1 [file DataSheet1.ZIP › original WB figures/Figure6/Figure6D-P-ERK.tif]

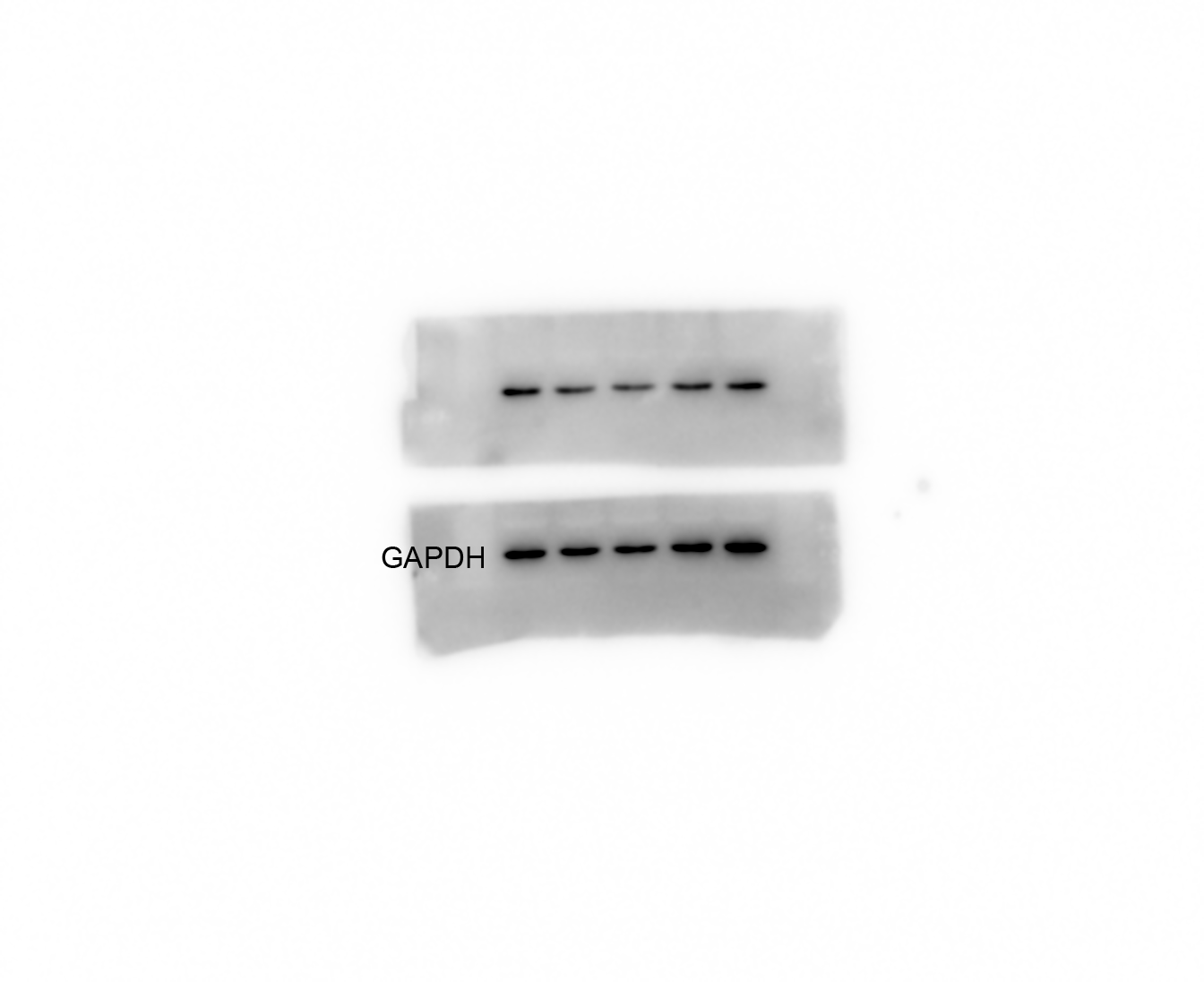

Supplement: Supplementary file 1 [file DataSheet1.ZIP › original WB figures/Figure6/Figure6E-GAPDH.tif]

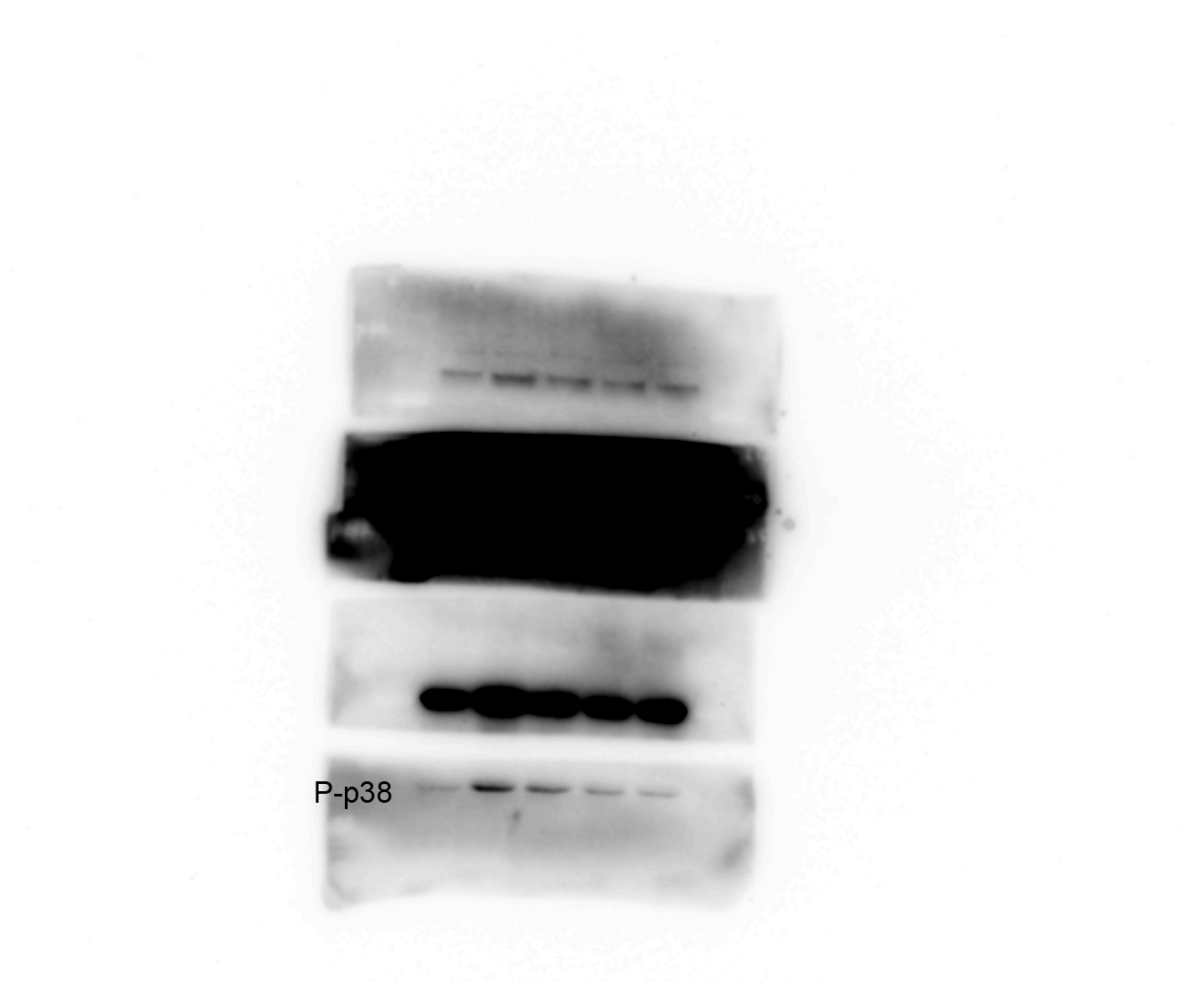

Supplement: Supplementary file 1 [file DataSheet1.ZIP › original WB figures/Figure6/Figure6E-P-p38.tif]

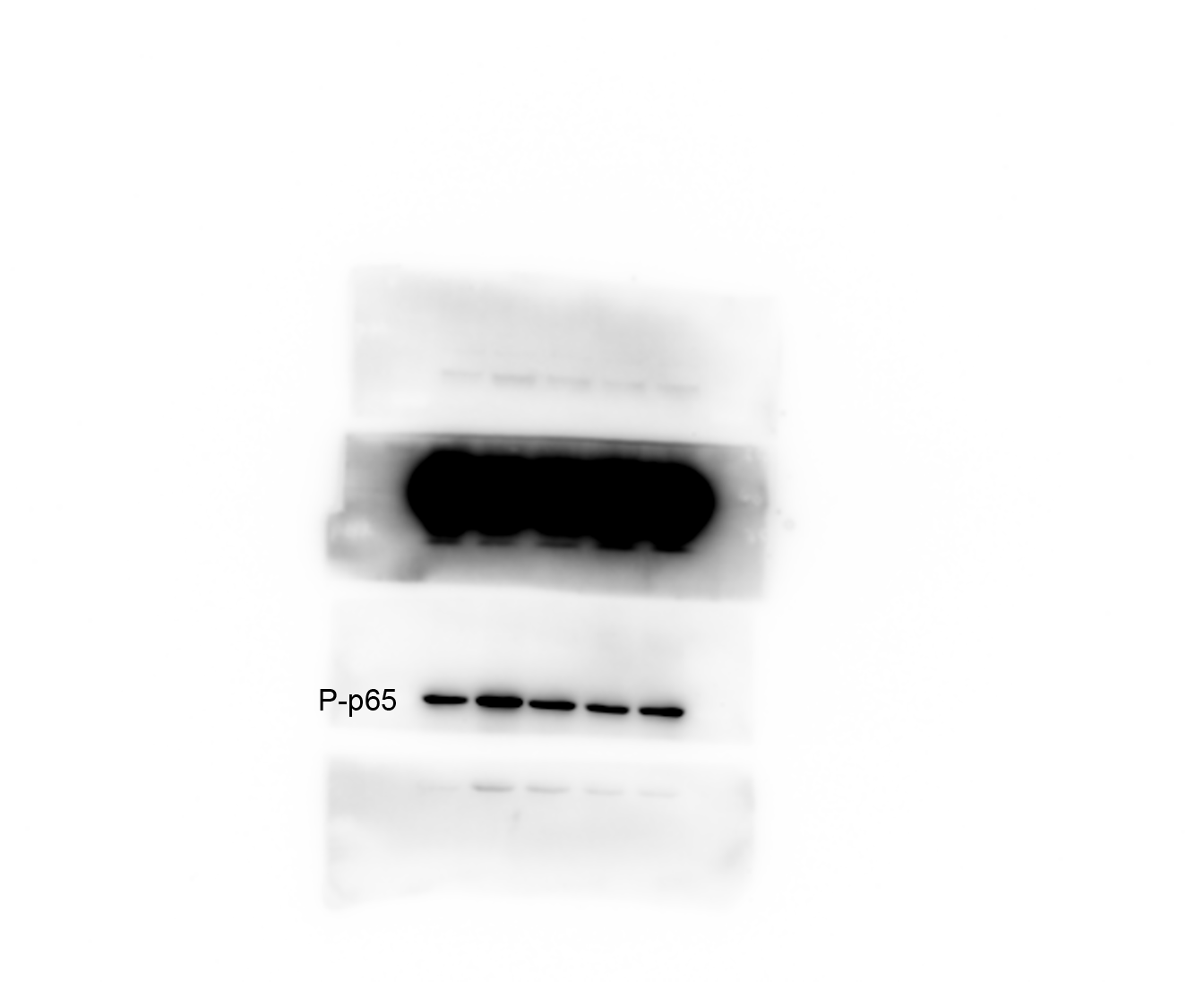

Supplement: Supplementary file 1 [file DataSheet1.ZIP › original WB figures/Figure6/Figure6E-P-p65.tif]

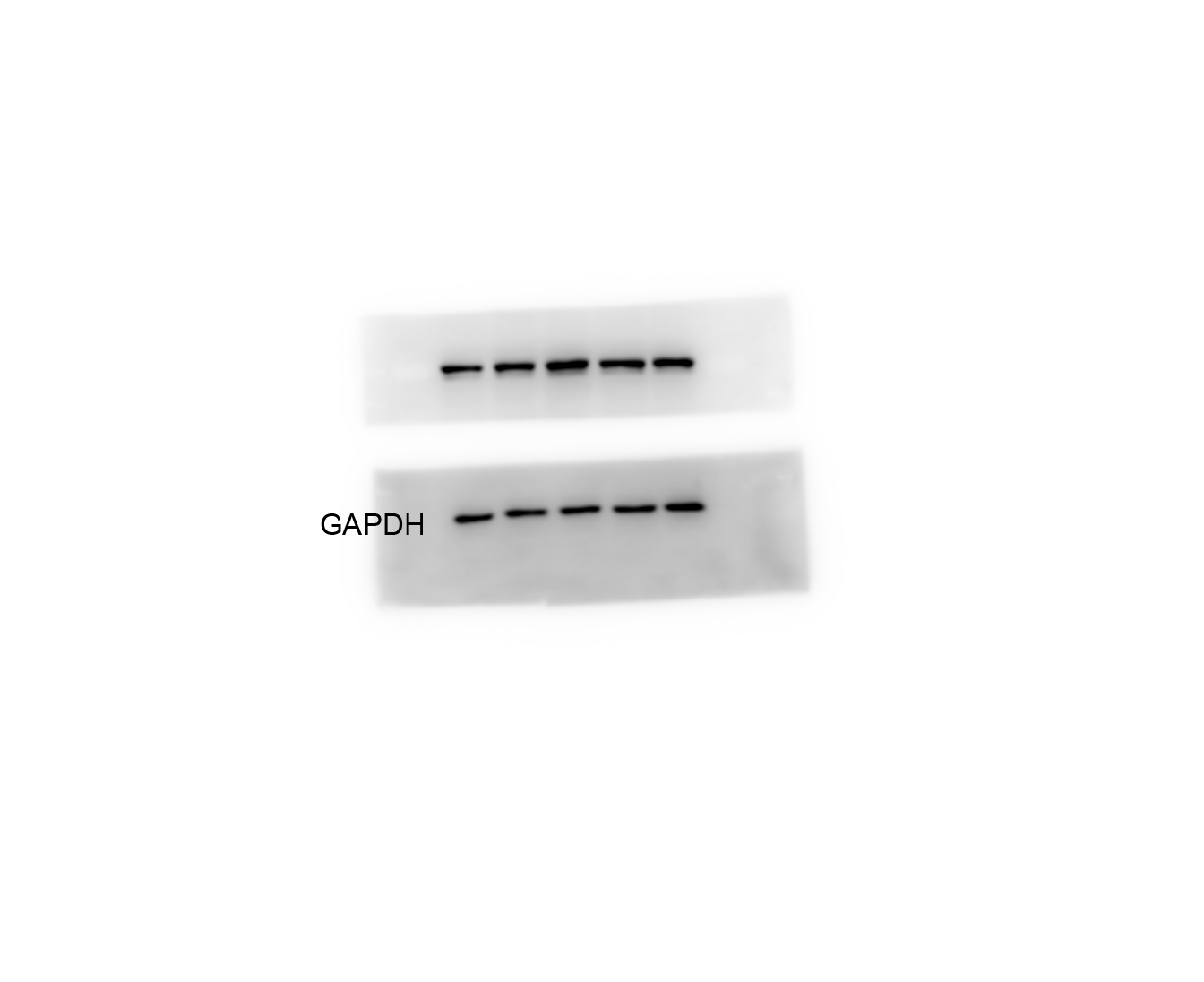

Supplement: Supplementary file 1 [file DataSheet1.ZIP › original WB figures/Figure6/Figure6F-GAPDH.tif]

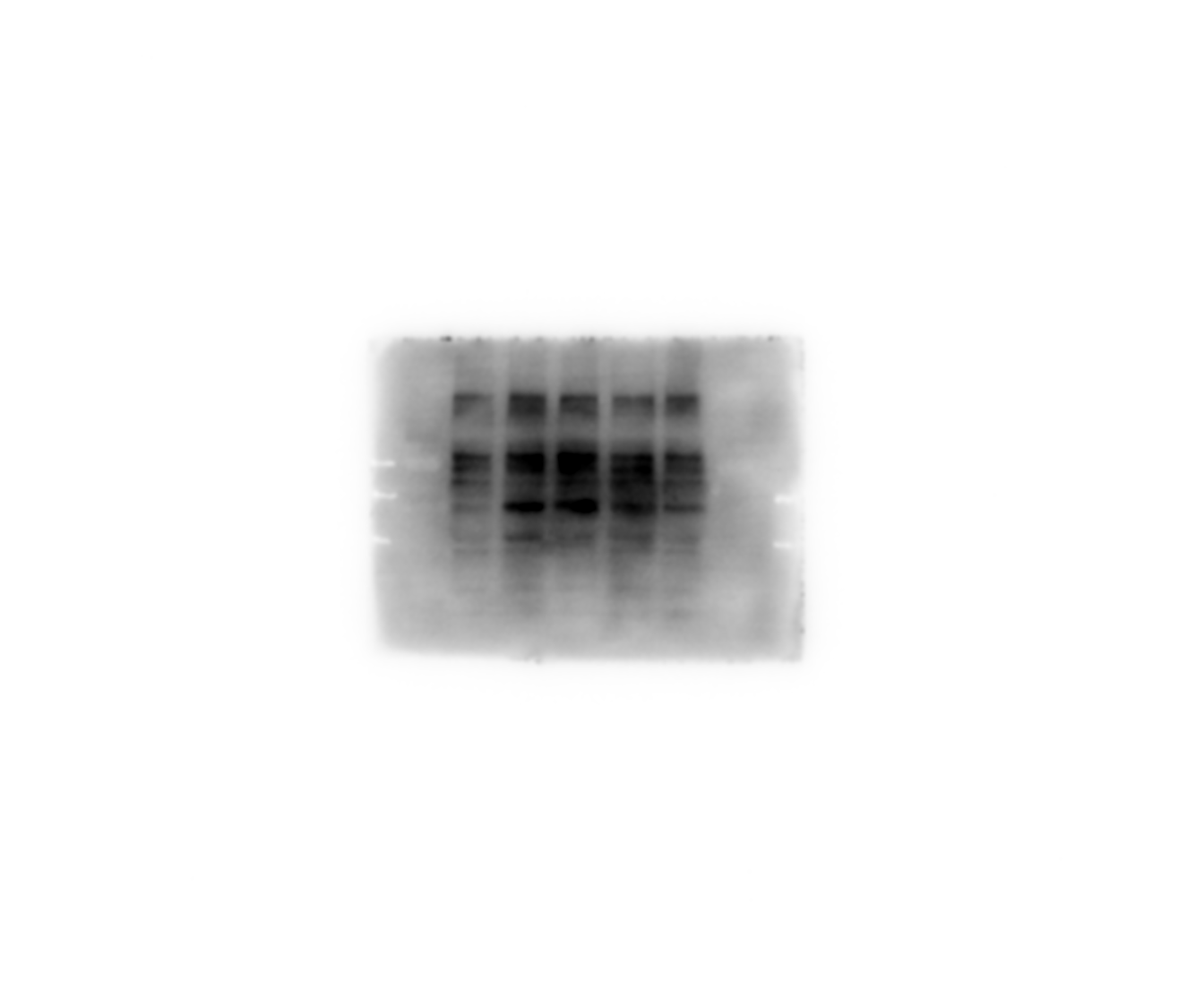

Supplement: Supplementary file 1 [file DataSheet1.ZIP › original WB figures/Figure6/Figure6F-P-JNK.tif]

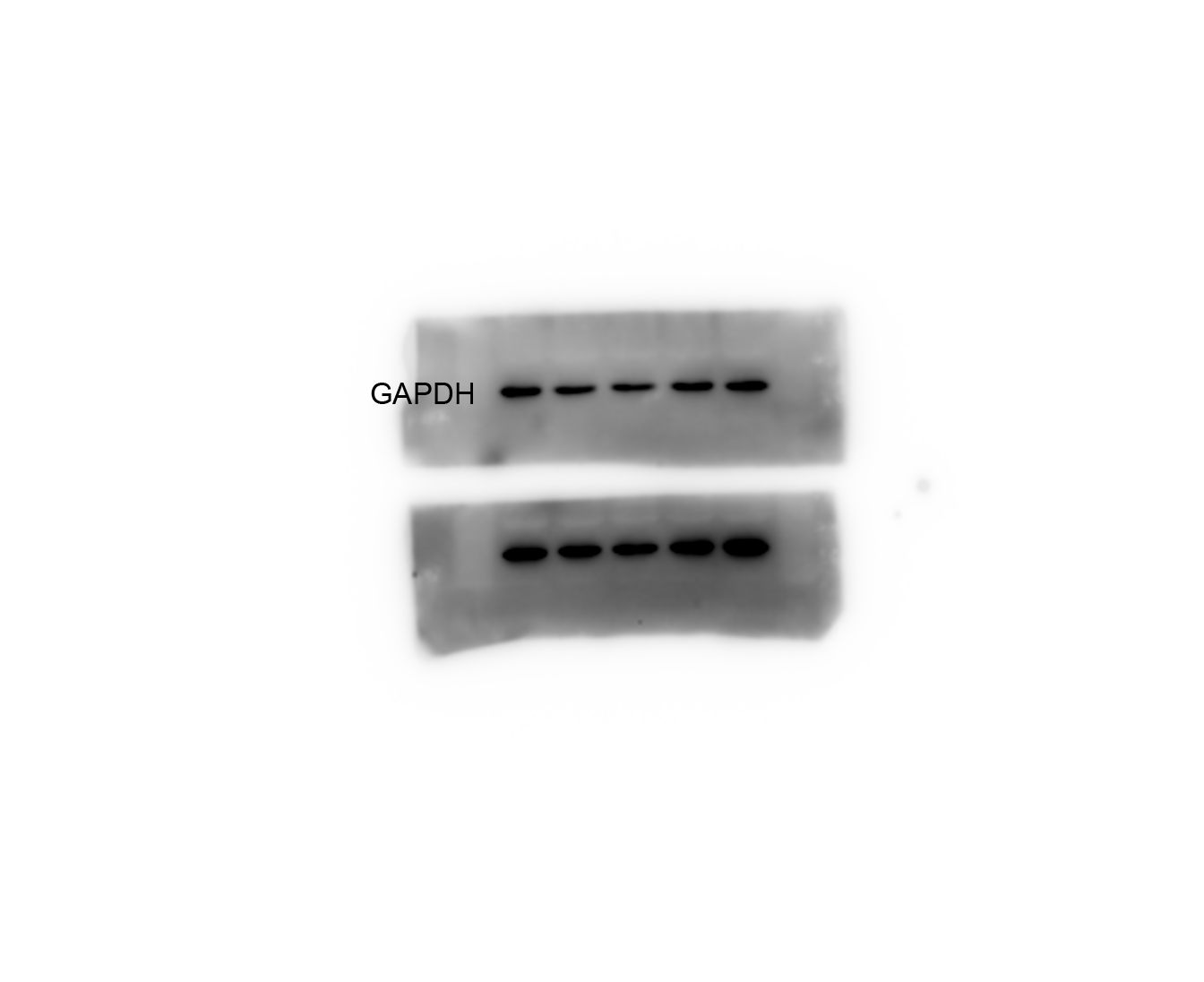

Supplement: Supplementary file 1 [file DataSheet1.ZIP › original WB figures/Figure6/Figure6G-GAPDH.tif]

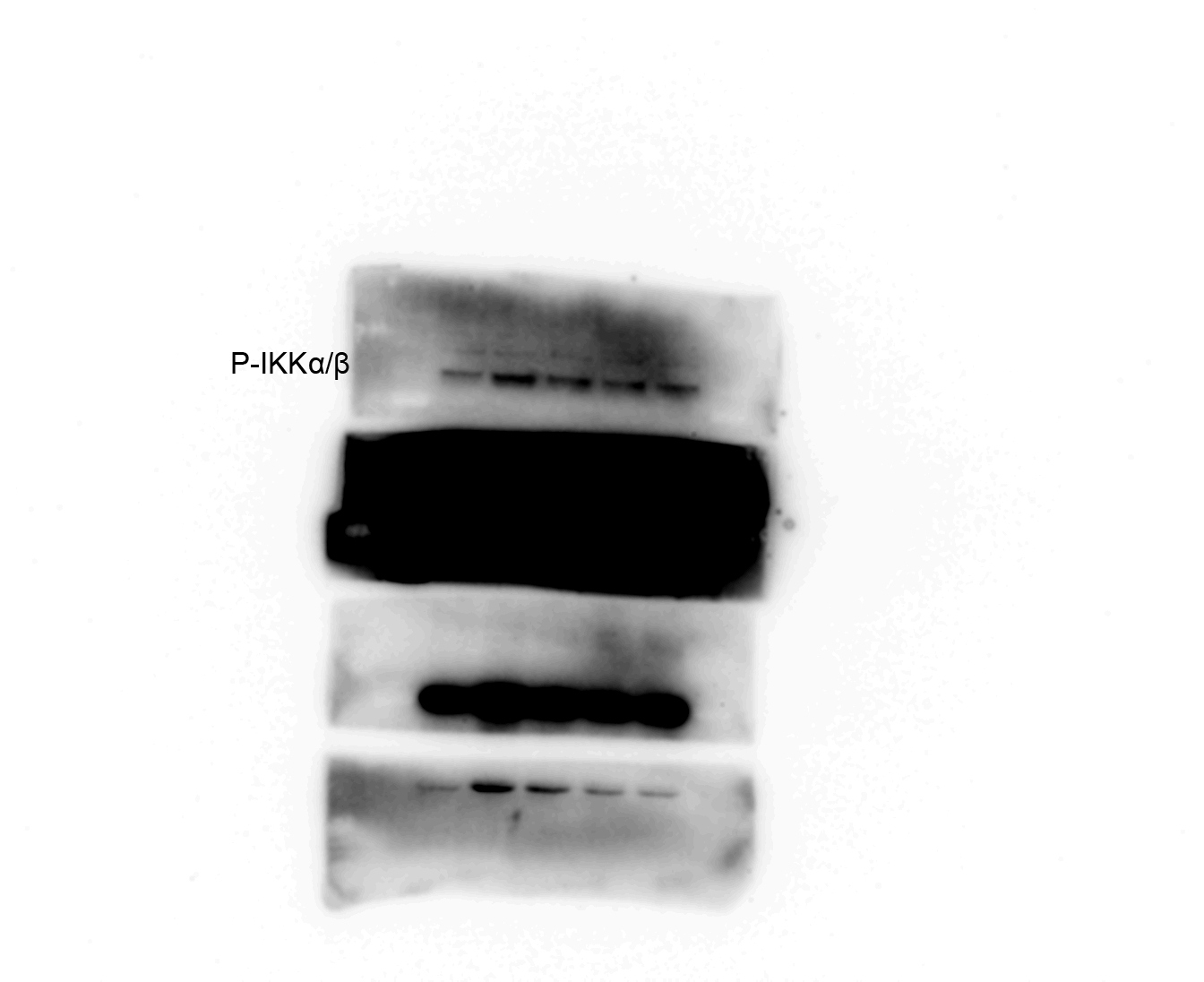

Supplement: Supplementary file 1 [file DataSheet1.ZIP › original WB figures/Figure6/Figure6G-P-IKKαβ.tif]

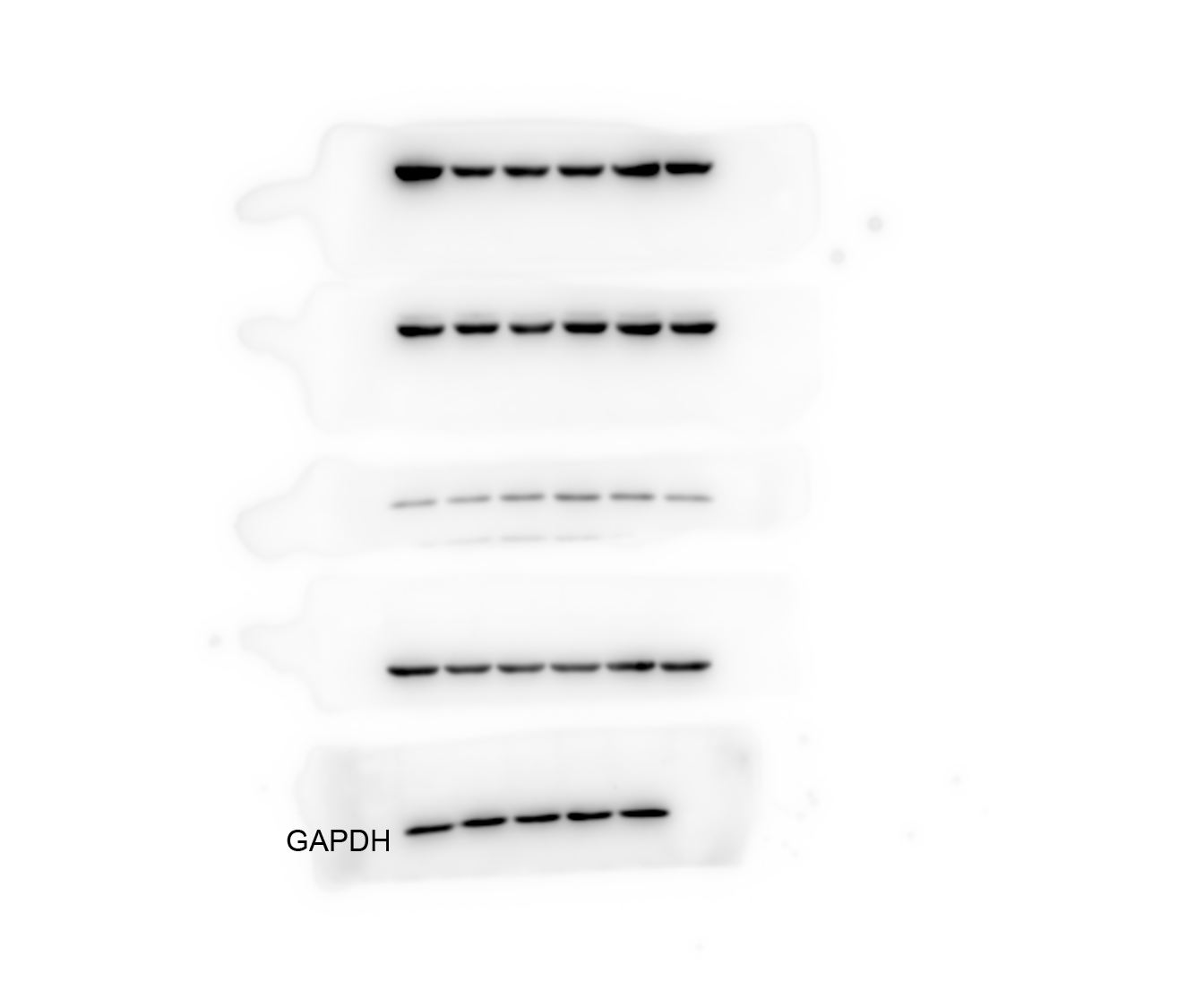

Supplement: Supplementary file 1 [file DataSheet1.ZIP › original WB figures/Figure6/Figure6H-GAPDH.tif]

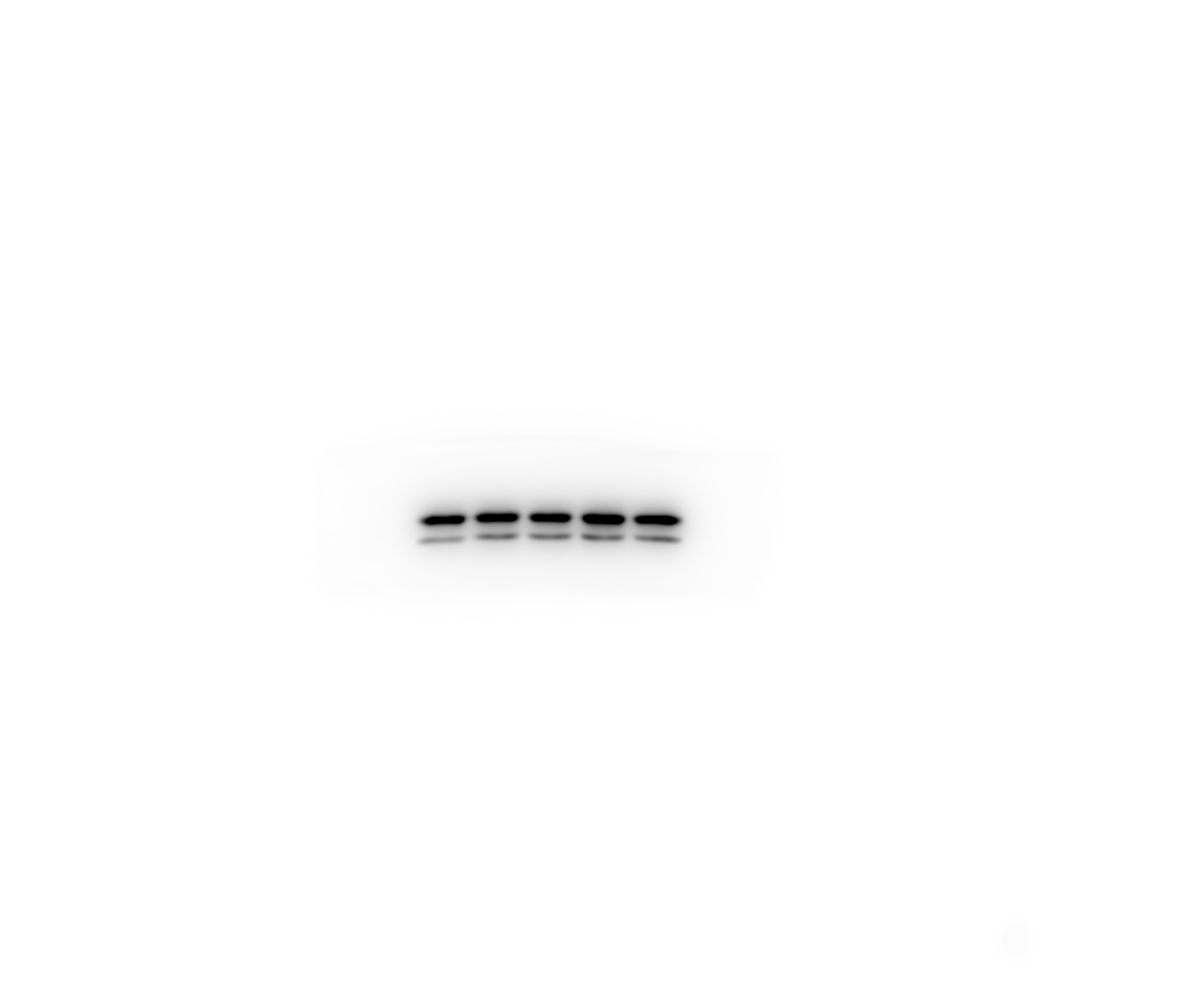

Supplement: Supplementary file 1 [file DataSheet1.ZIP › original WB figures/Figure6/Figure6H-IκBα.tif]

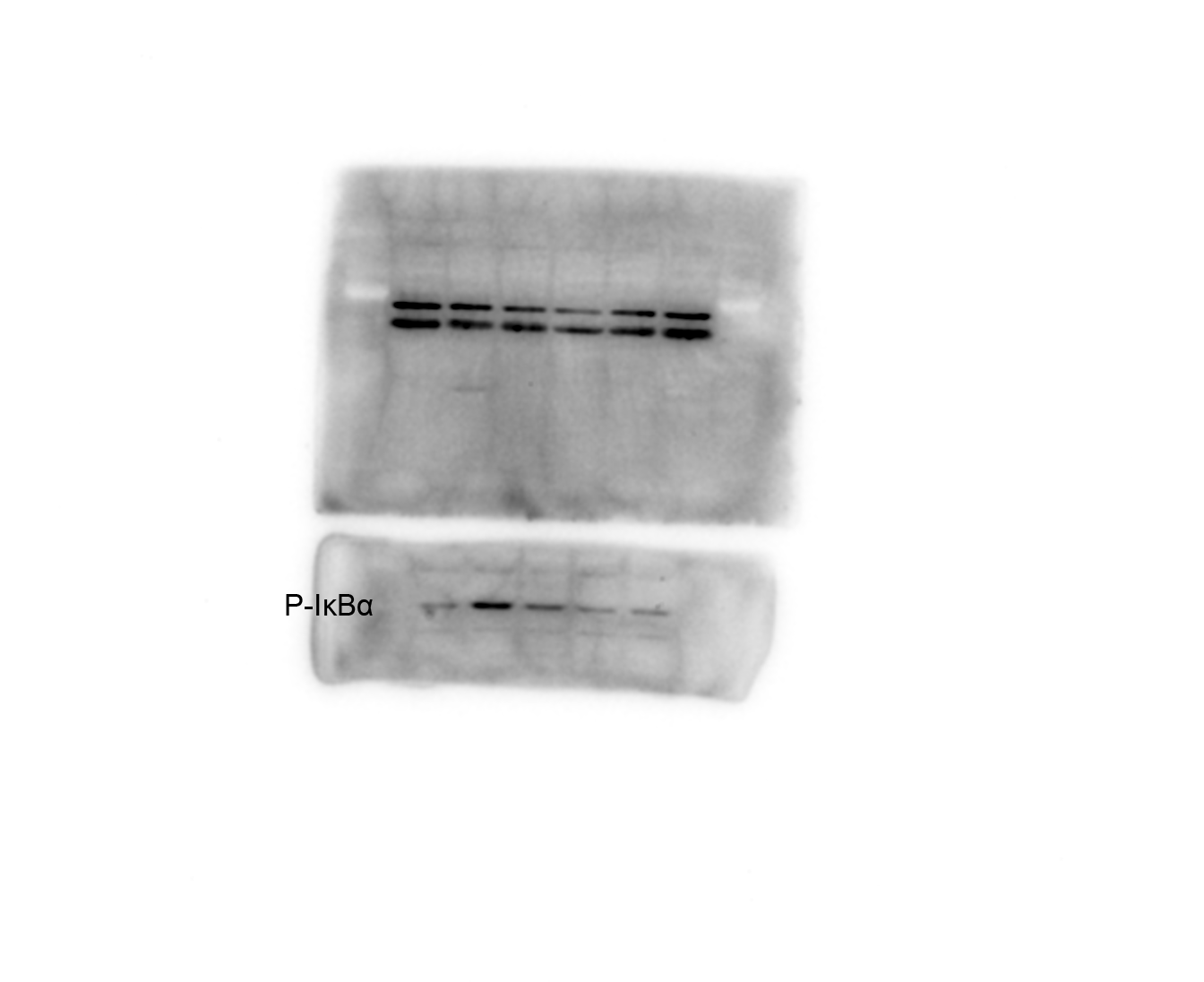

Supplement: Supplementary file 1 [file DataSheet1.ZIP › original WB figures/Figure6/Figure6H-P-IκBα.tif]

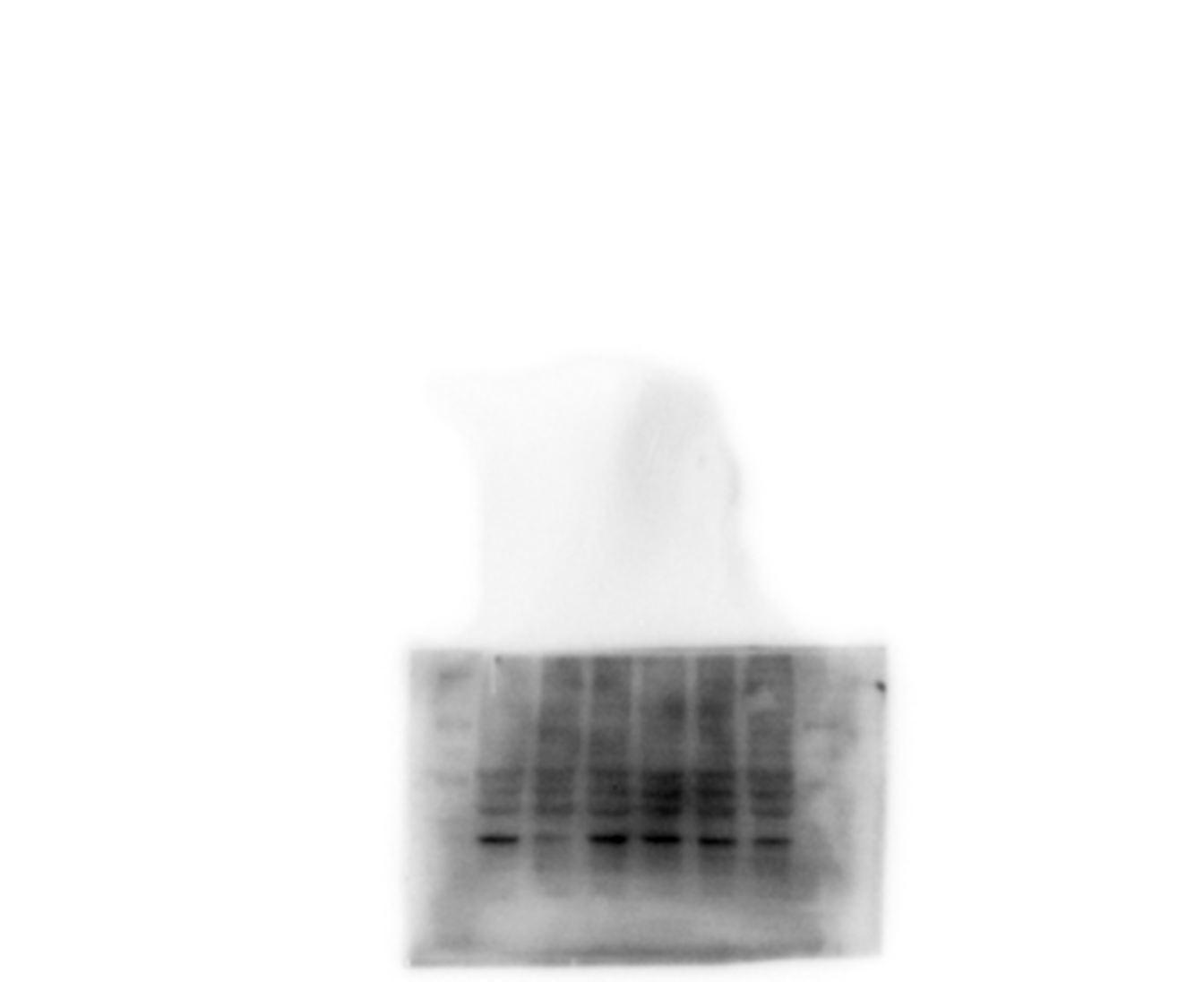

Supplement: Supplementary file 1 [file DataSheet1.ZIP › original WB figures/Figure7/Figure7A-IL-1β-in Lys.tif]

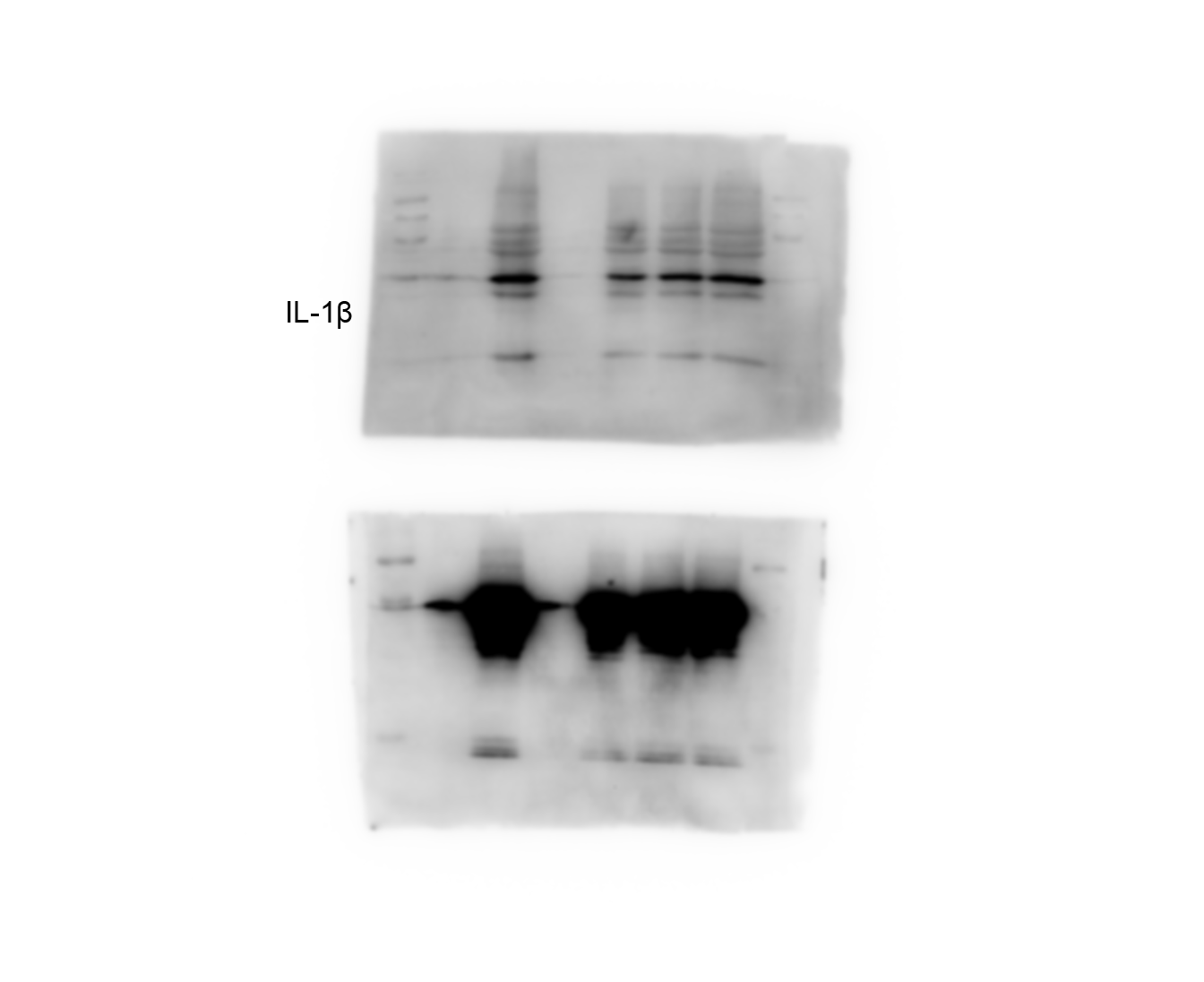

Supplement: Supplementary file 1 [file DataSheet1.ZIP › original WB figures/Figure7/Figure7A-IL-1β-in Sup.tif]

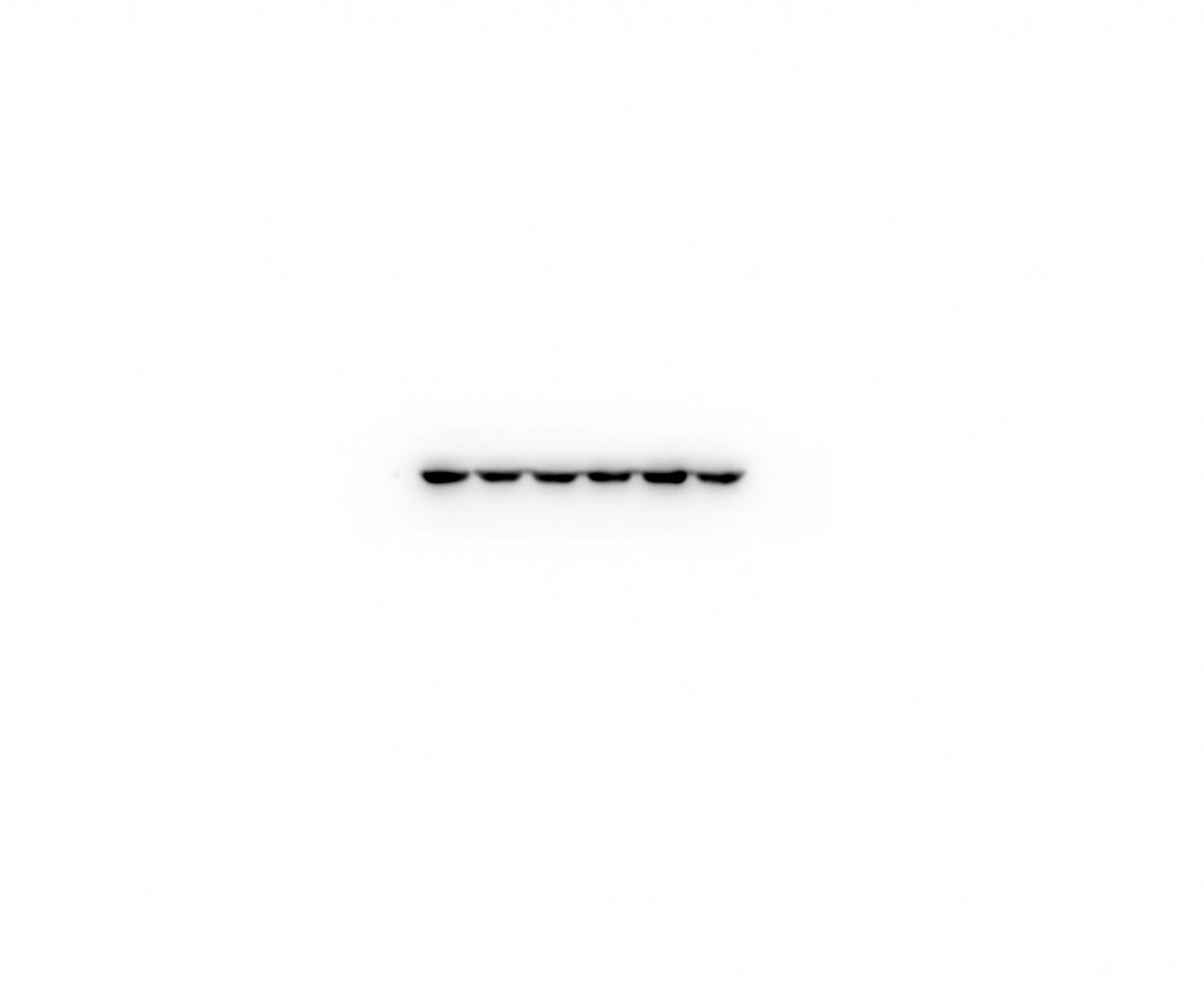

Supplement: Supplementary file 1 [file DataSheet1.ZIP › original WB figures/Figure7/Figure7A-β-actin-in Lys.tif]

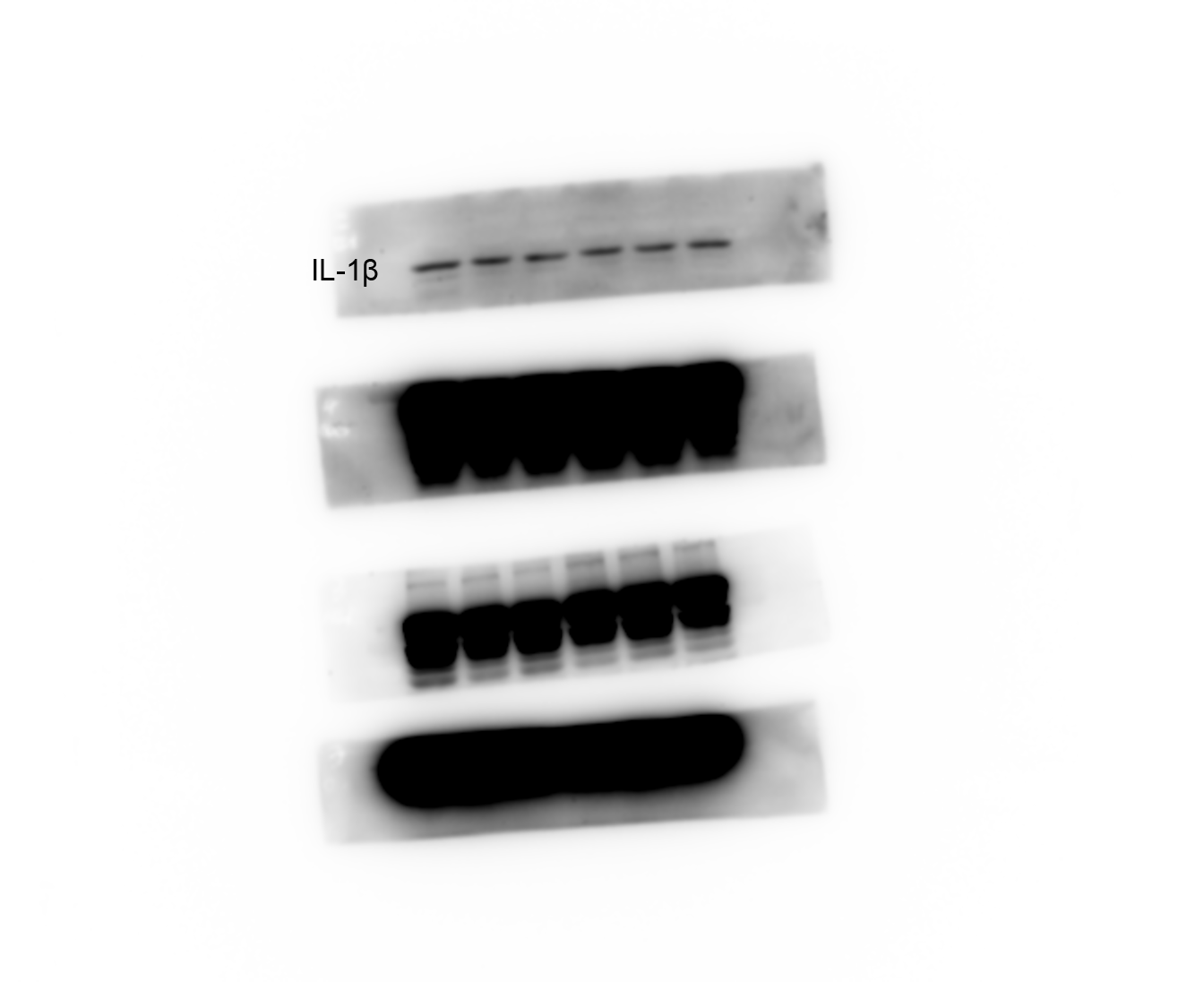

Supplement: Supplementary file 1 [file DataSheet1.ZIP › original WB figures/Figure7/Figure7B-IL-1β-in Lys.tif]

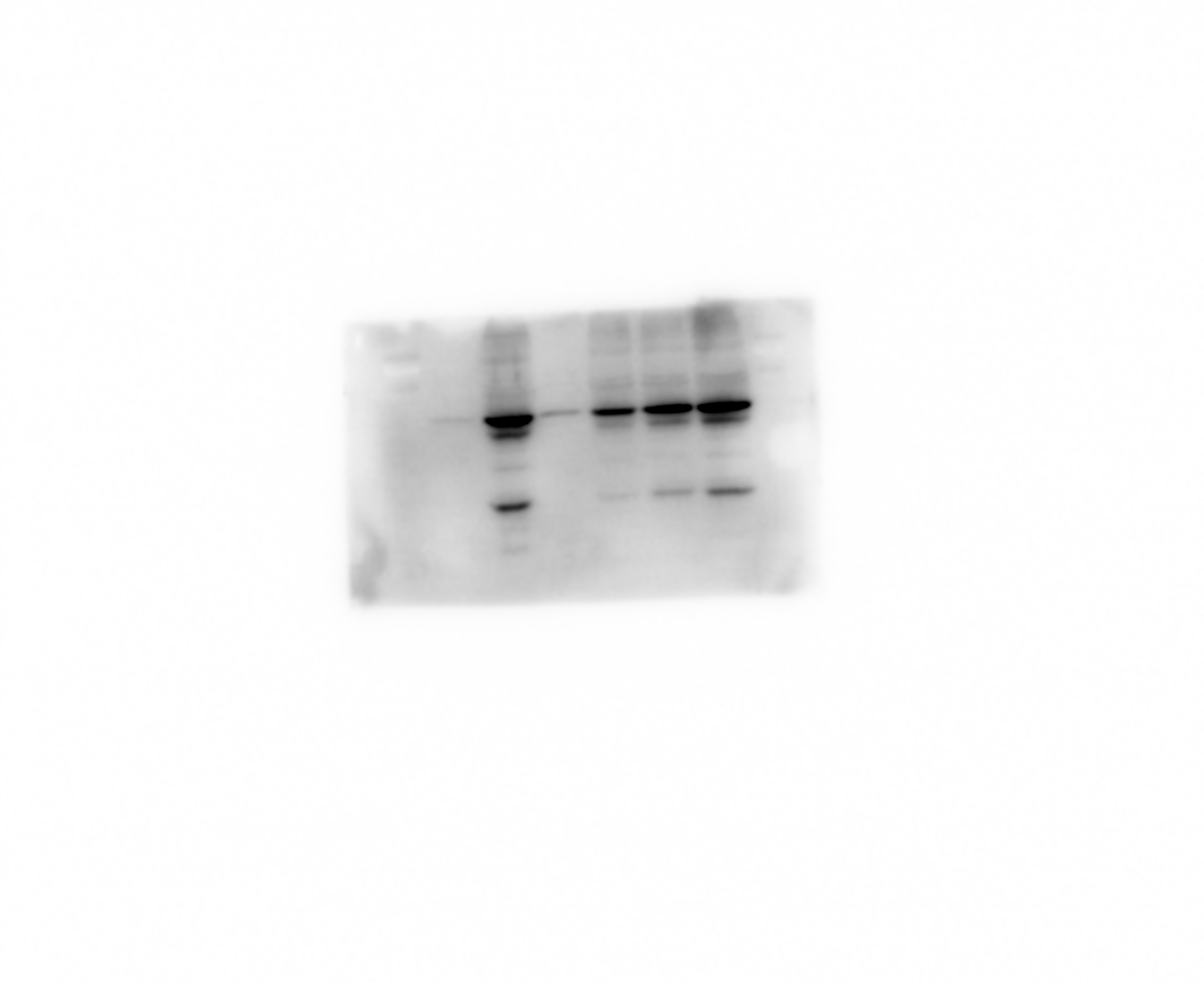

Supplement: Supplementary file 1 [file DataSheet1.ZIP › original WB figures/Figure7/Figure7B-IL-1β-in Sup.tif]

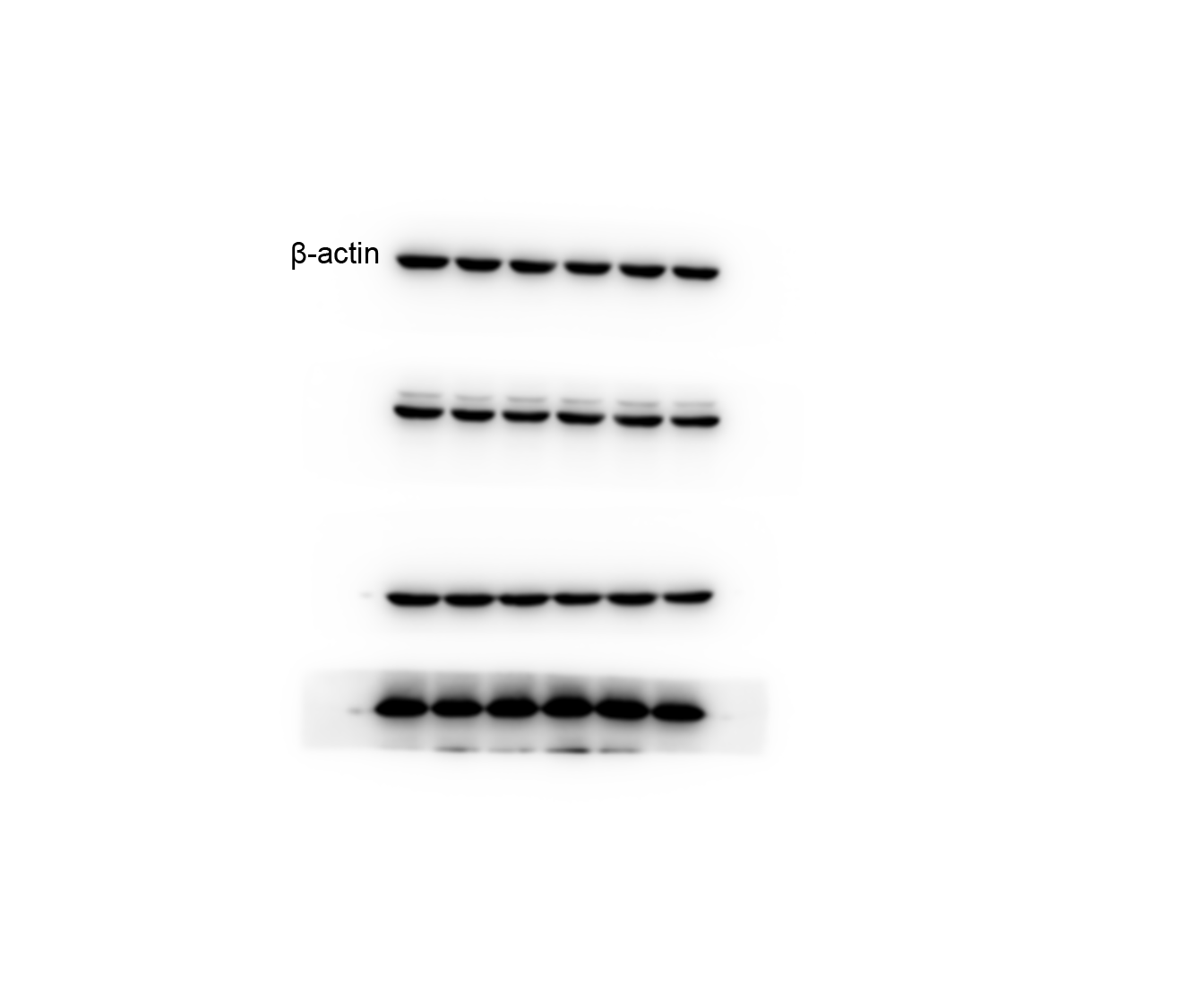

Supplement: Supplementary file 1 [file DataSheet1.ZIP › original WB figures/Figure7/Figure7B-β-actin-in Lys.tif]

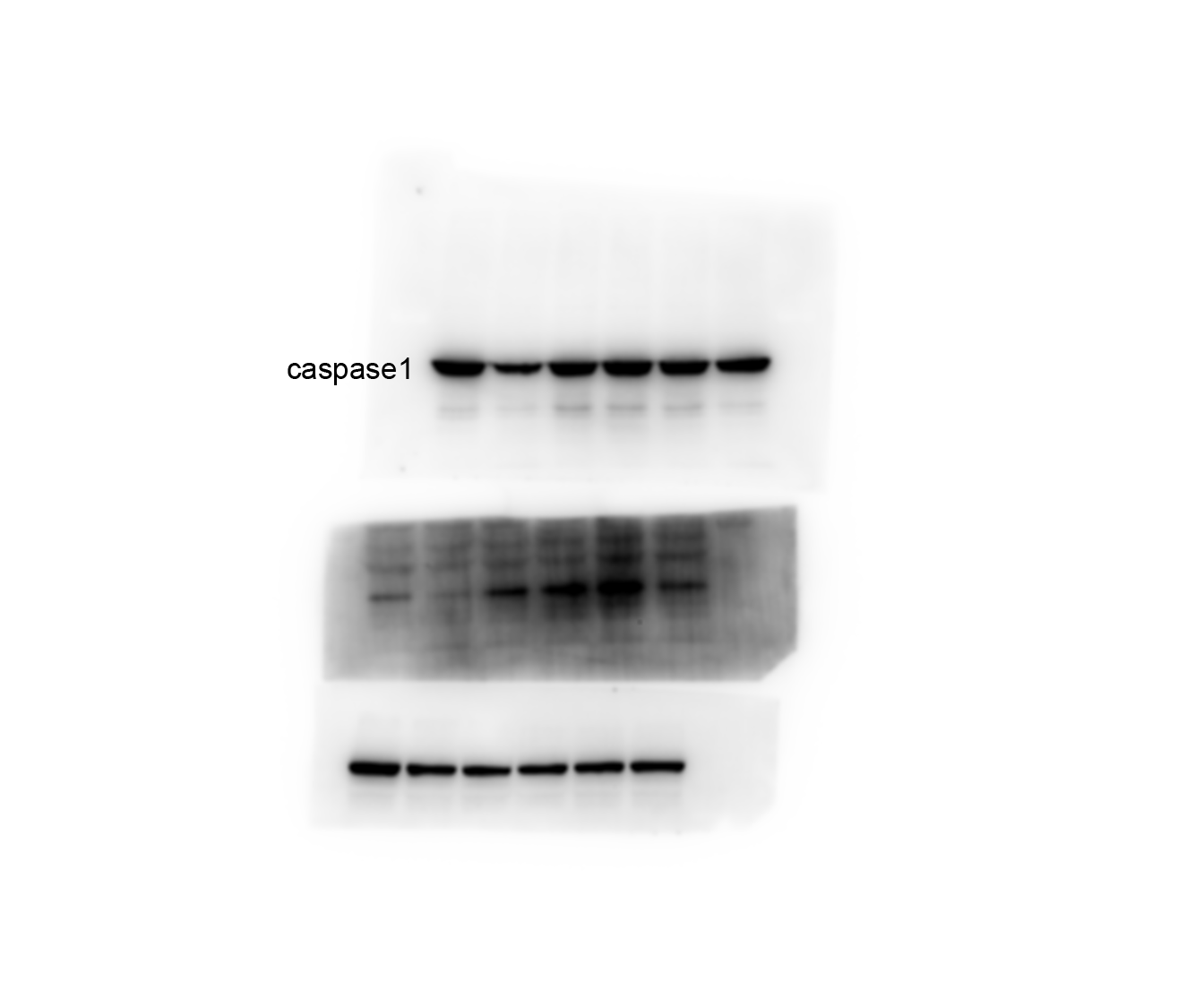

Supplement: Supplementary file 1 [file DataSheet1.ZIP › original WB figures/Figure7/Figure7C-caspase1-in Lys.tif]

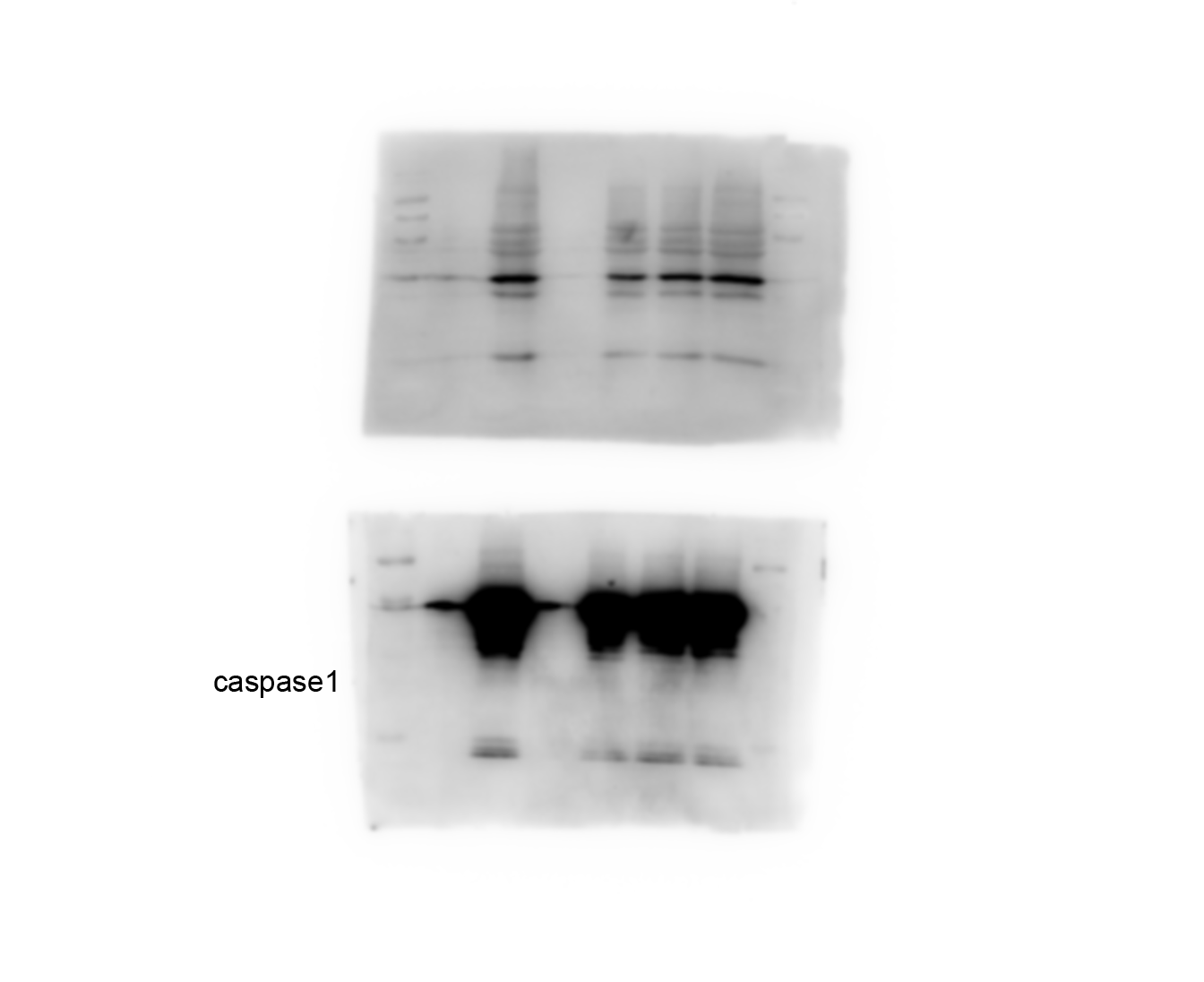

Supplement: Supplementary file 1 [file DataSheet1.ZIP › original WB figures/Figure7/Figure7C-caspase1-in Sup.tif]

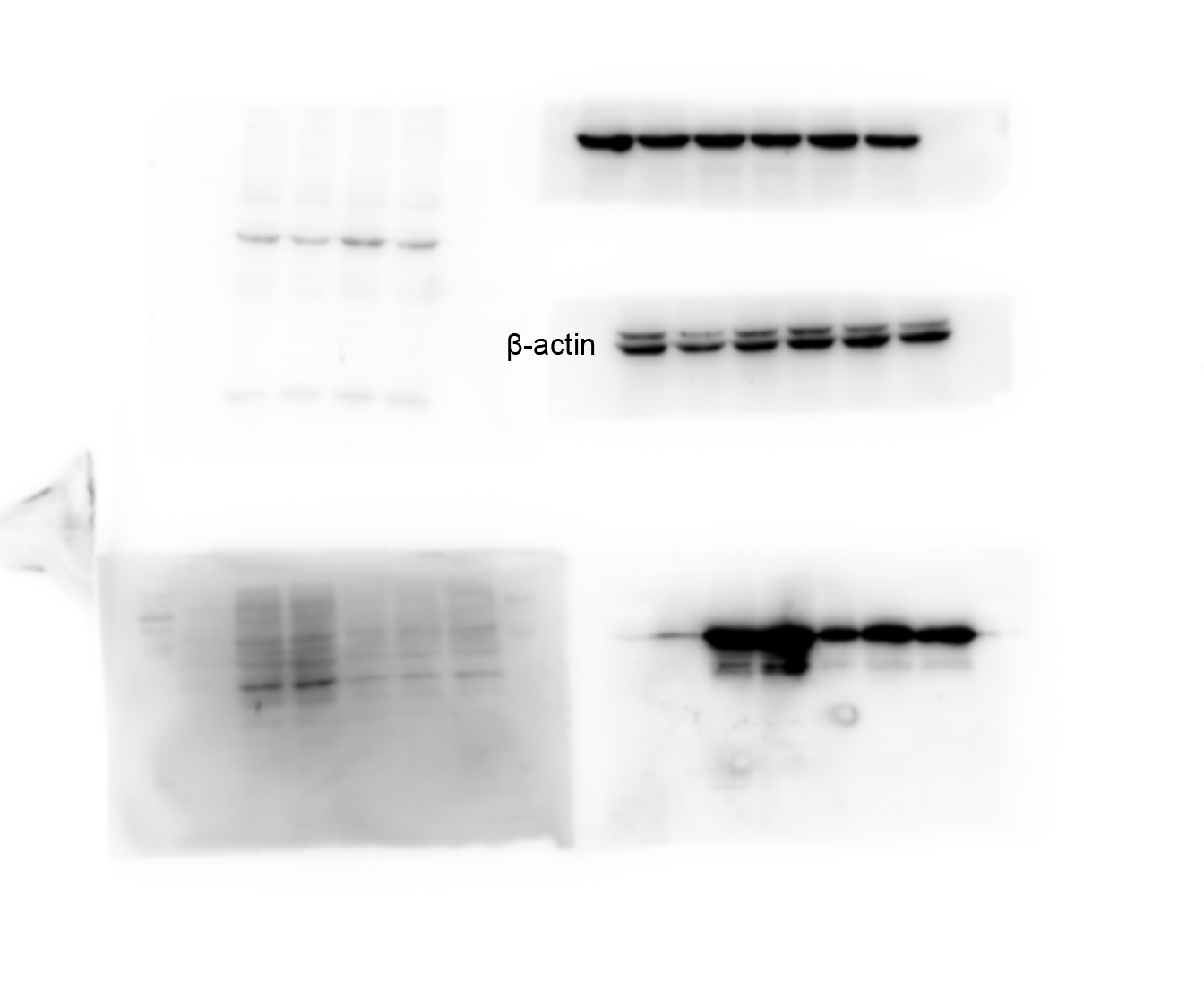

Supplement: Supplementary file 1 [file DataSheet1.ZIP › original WB figures/Figure7/Figure7C-β-actin-in Lys.tif]

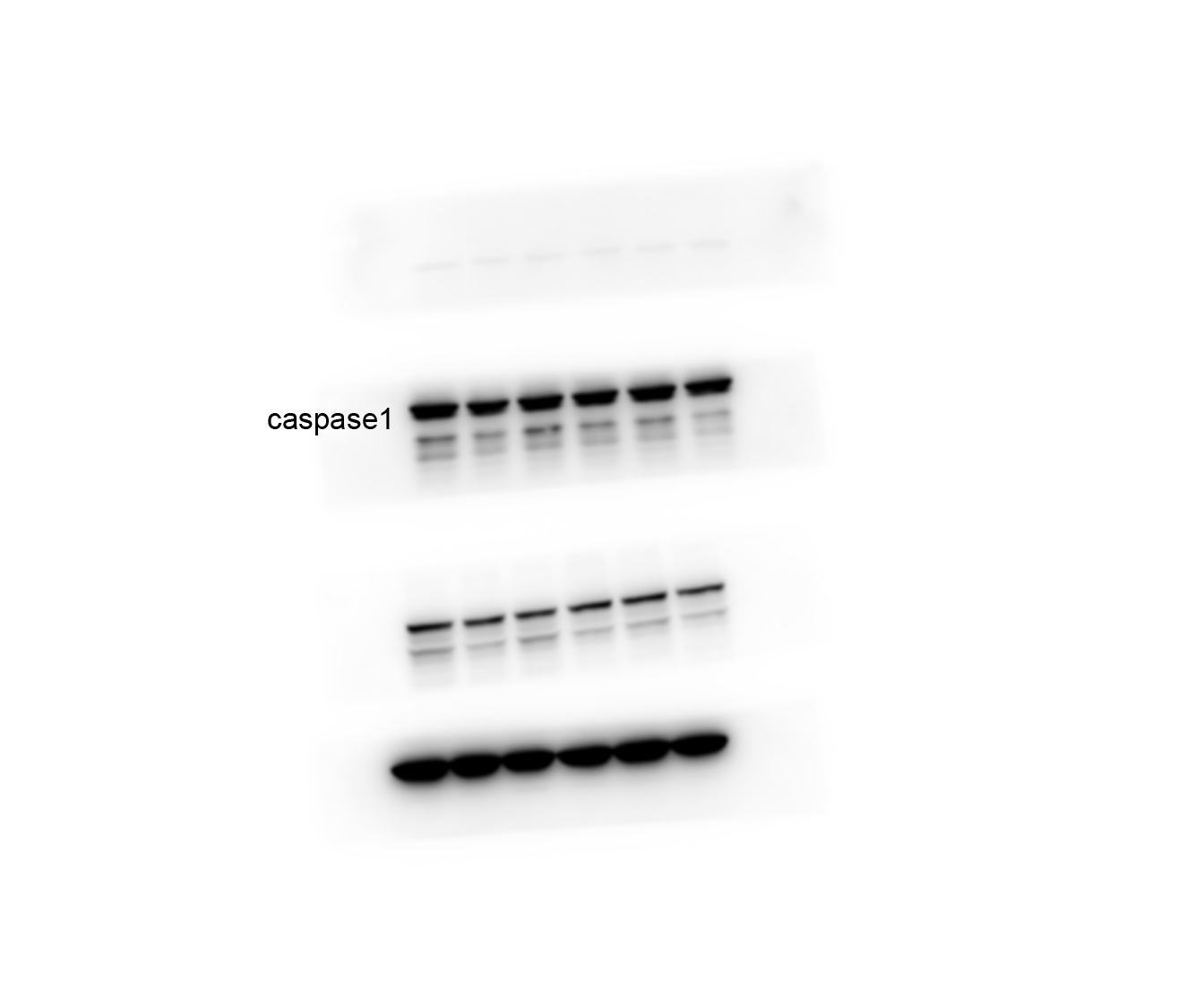

Supplement: Supplementary file 1 [file DataSheet1.ZIP › original WB figures/Figure7/Figure7D-caspase1-in Lys.tif]

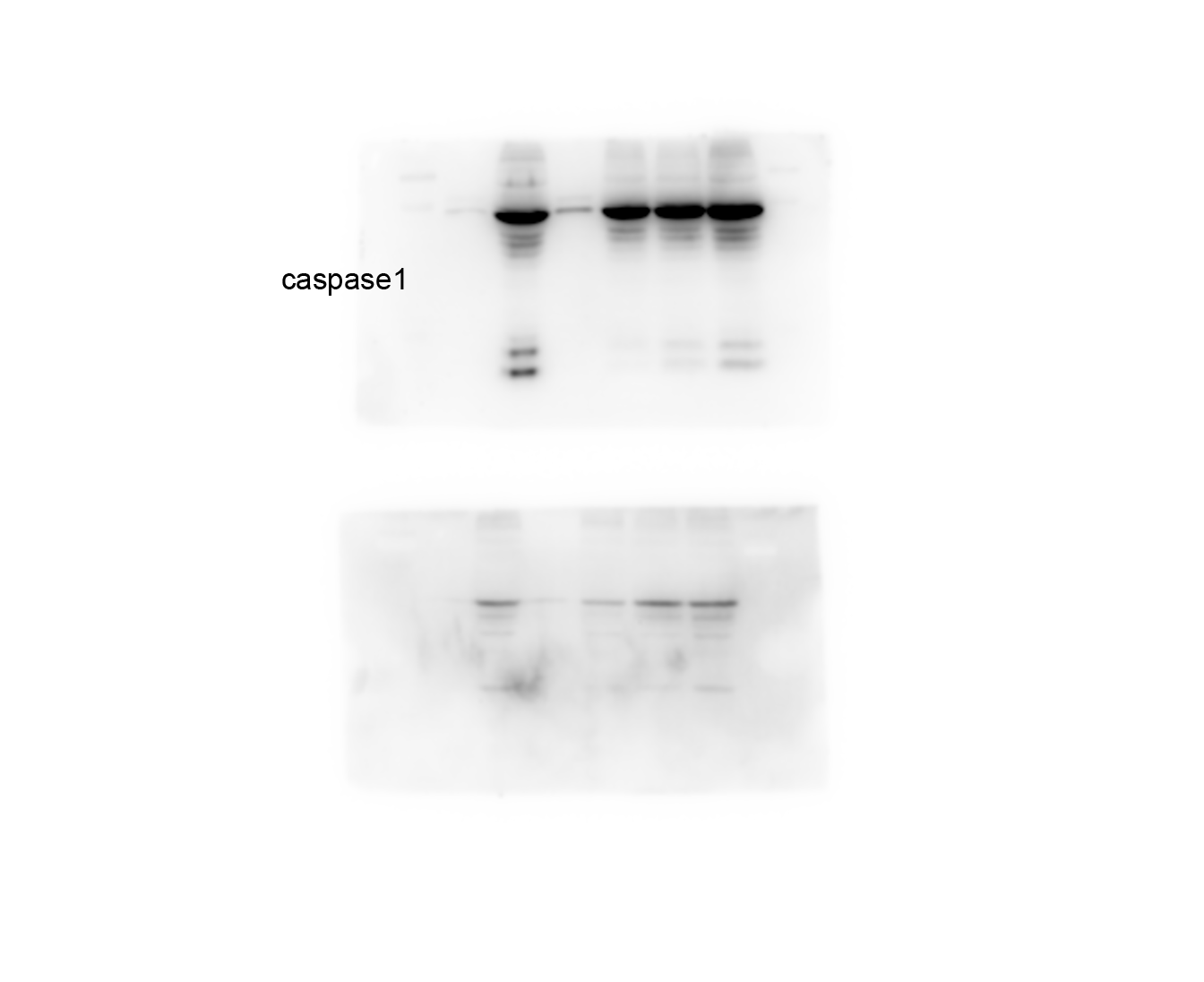

Supplement: Supplementary file 1 [file DataSheet1.ZIP › original WB figures/Figure7/Figure7D-caspase1-in Sup.tif]

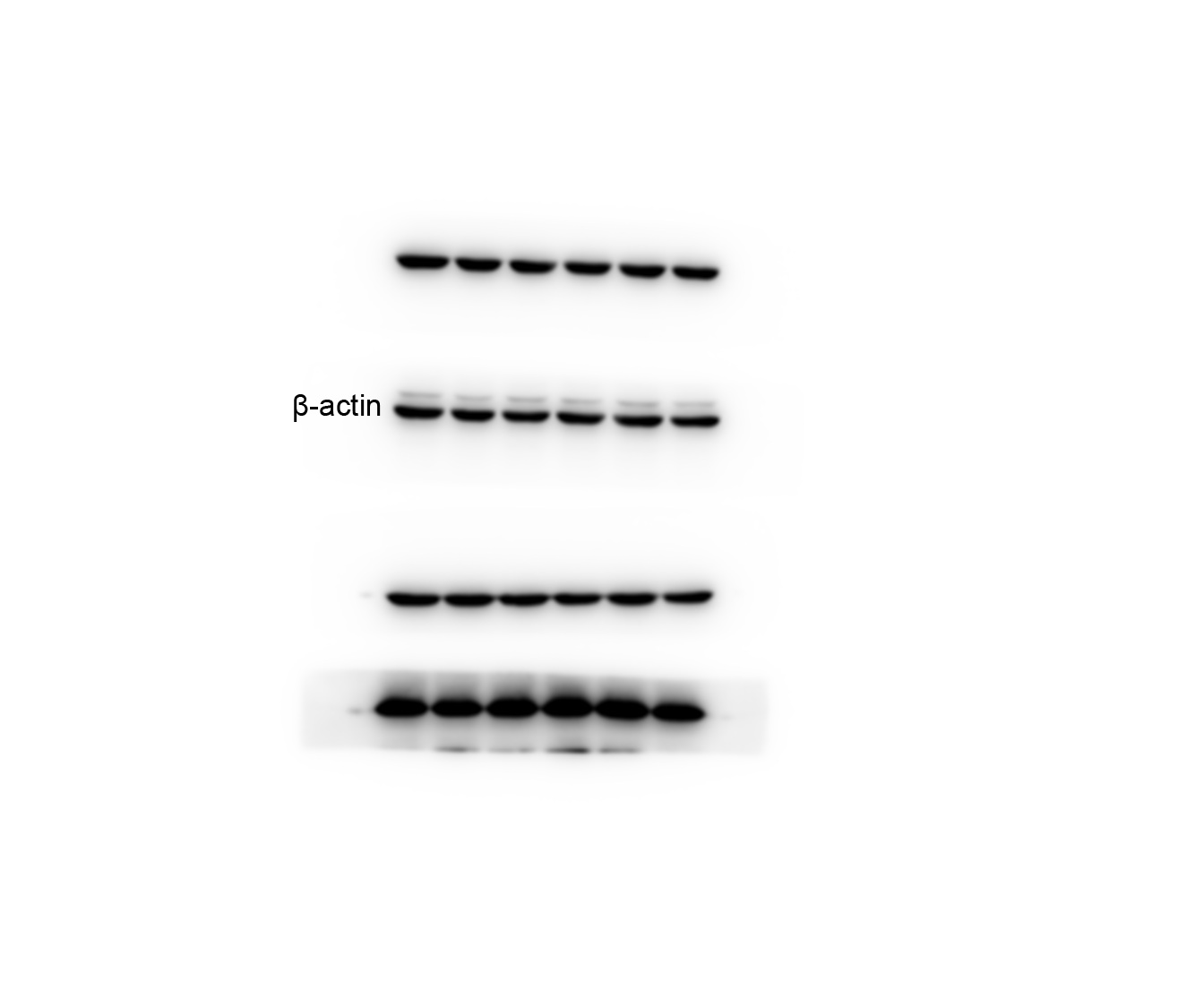

Supplement: Supplementary file 1 [file DataSheet1.ZIP › original WB figures/Figure7/Figure7D-β-actin-in Lys.tif]

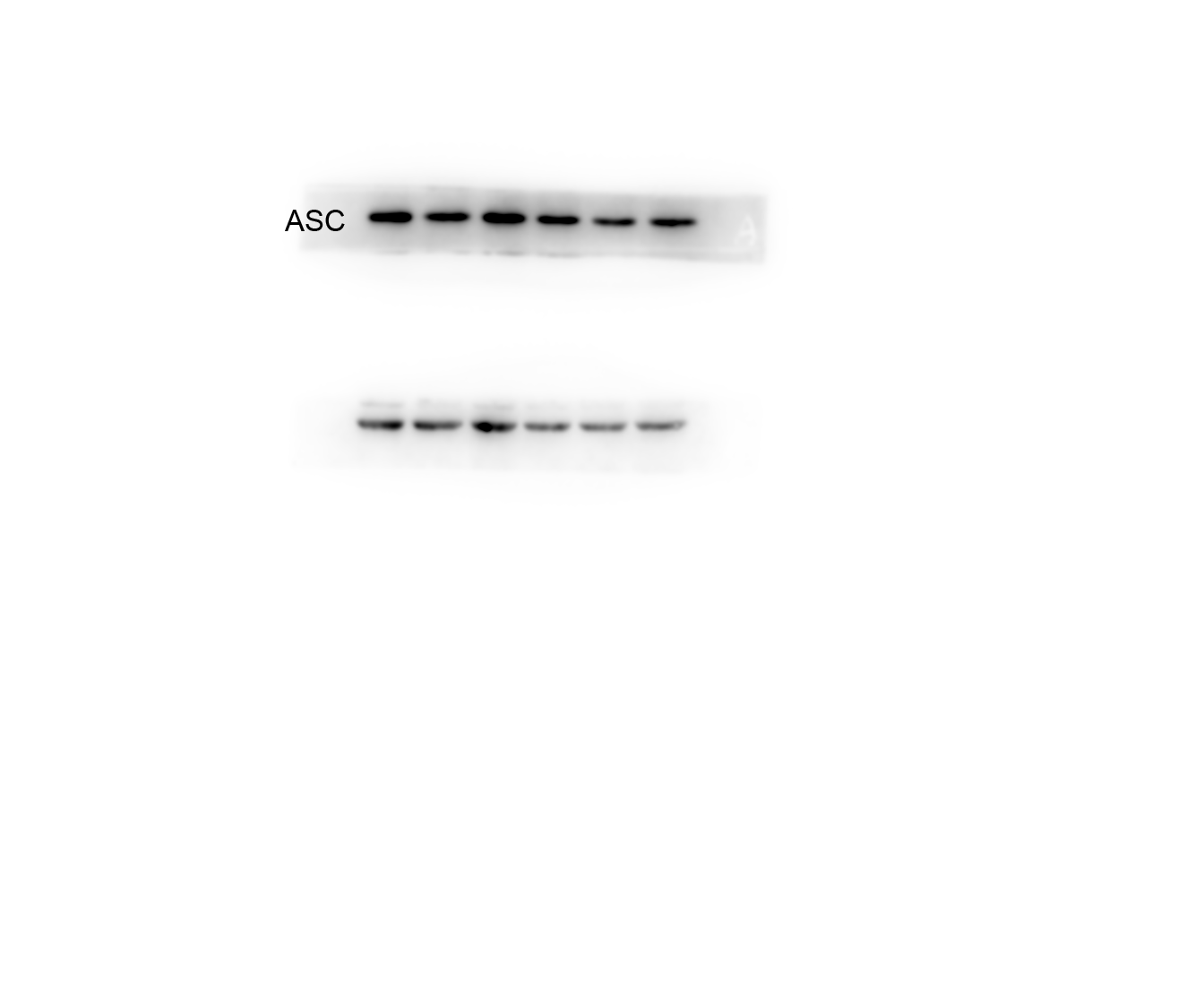

Supplement: Supplementary file 1 [file DataSheet1.ZIP › original WB figures/Figure8/Figure8A-ASC.tif]

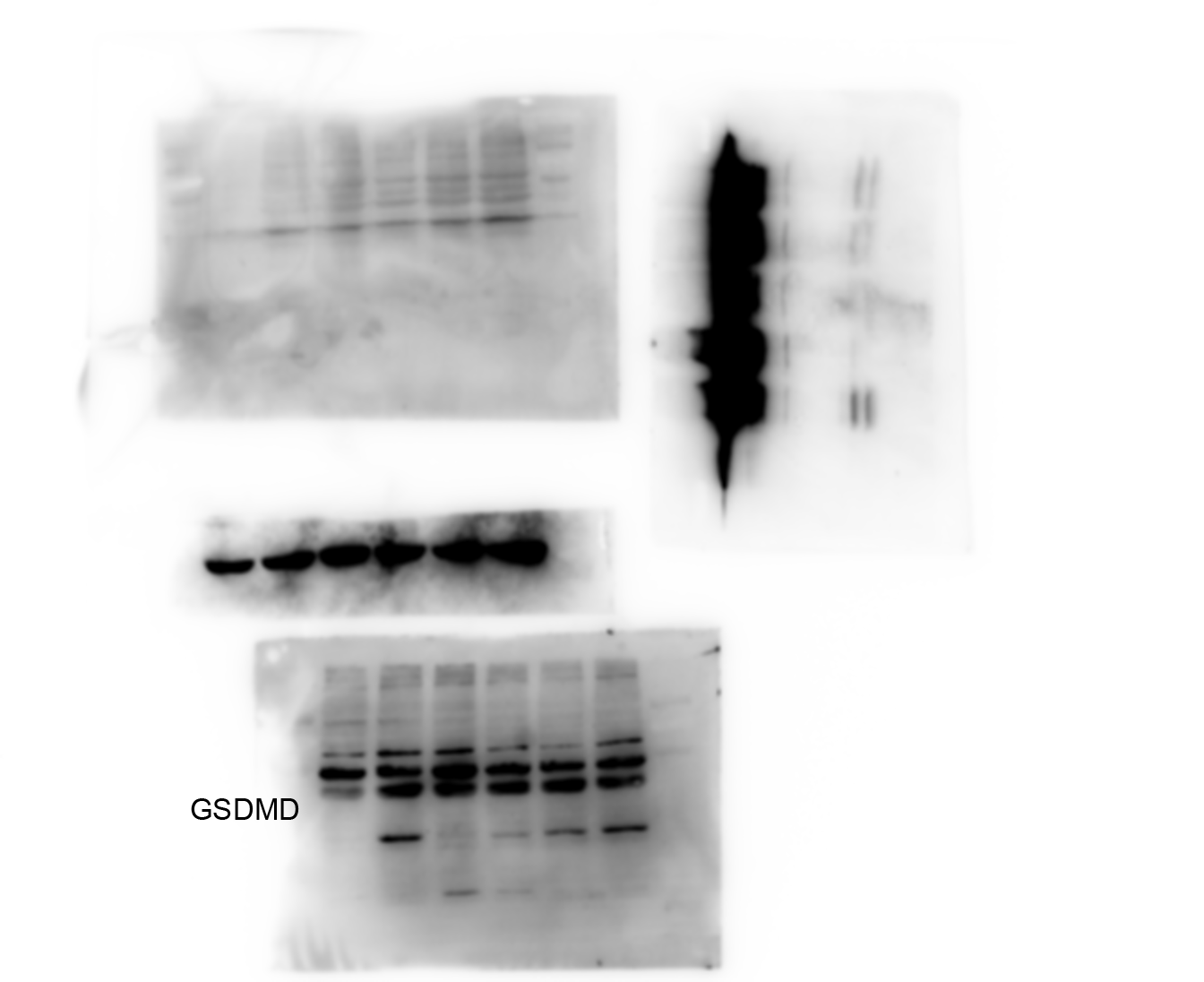

Supplement: Supplementary file 1 [file DataSheet1.ZIP › original WB figures/Figure8/Figure8A-GSDMD.tif]

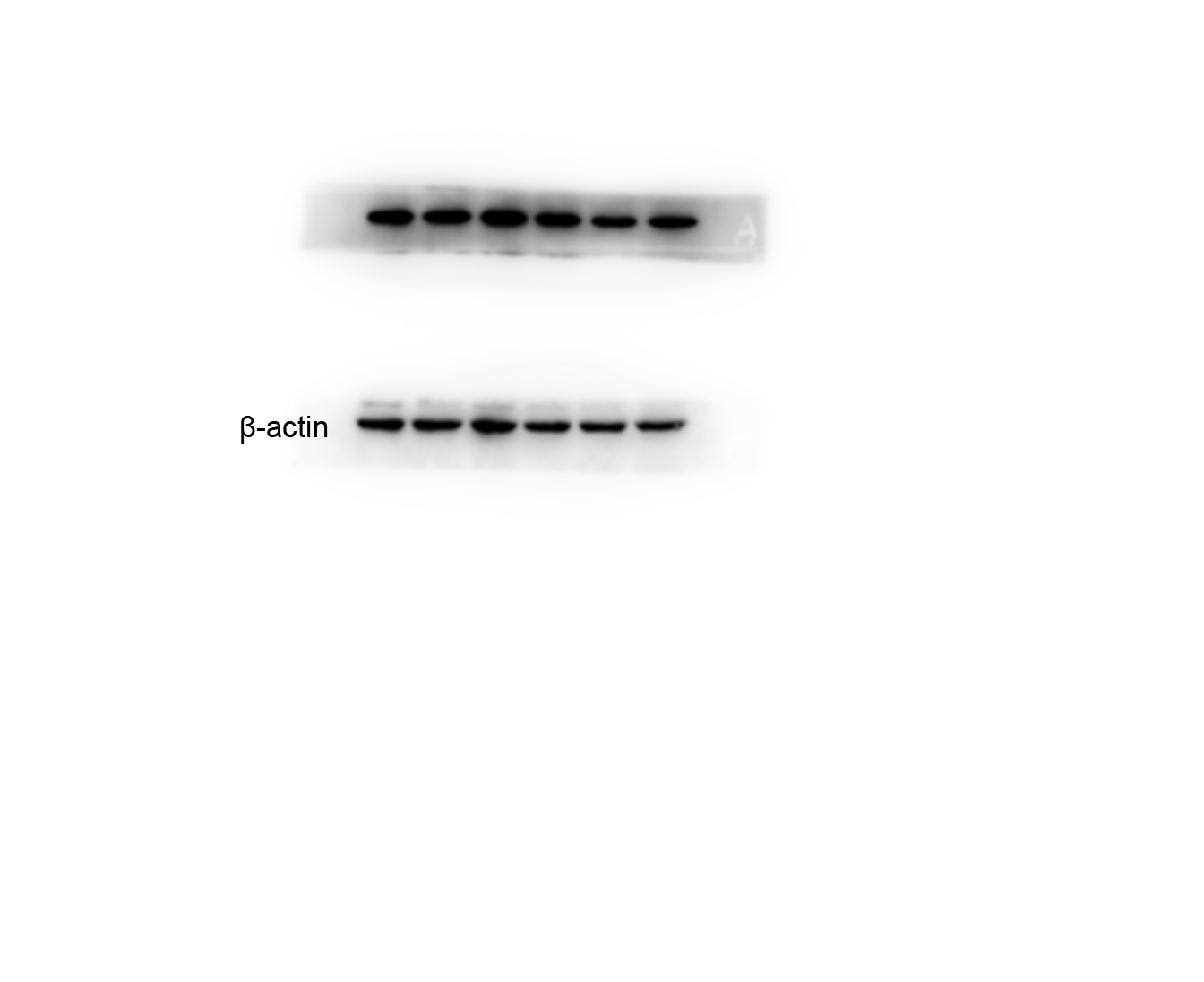

Supplement: Supplementary file 1 [file DataSheet1.ZIP › original WB figures/Figure8/Figure8A-β-actin.tif]

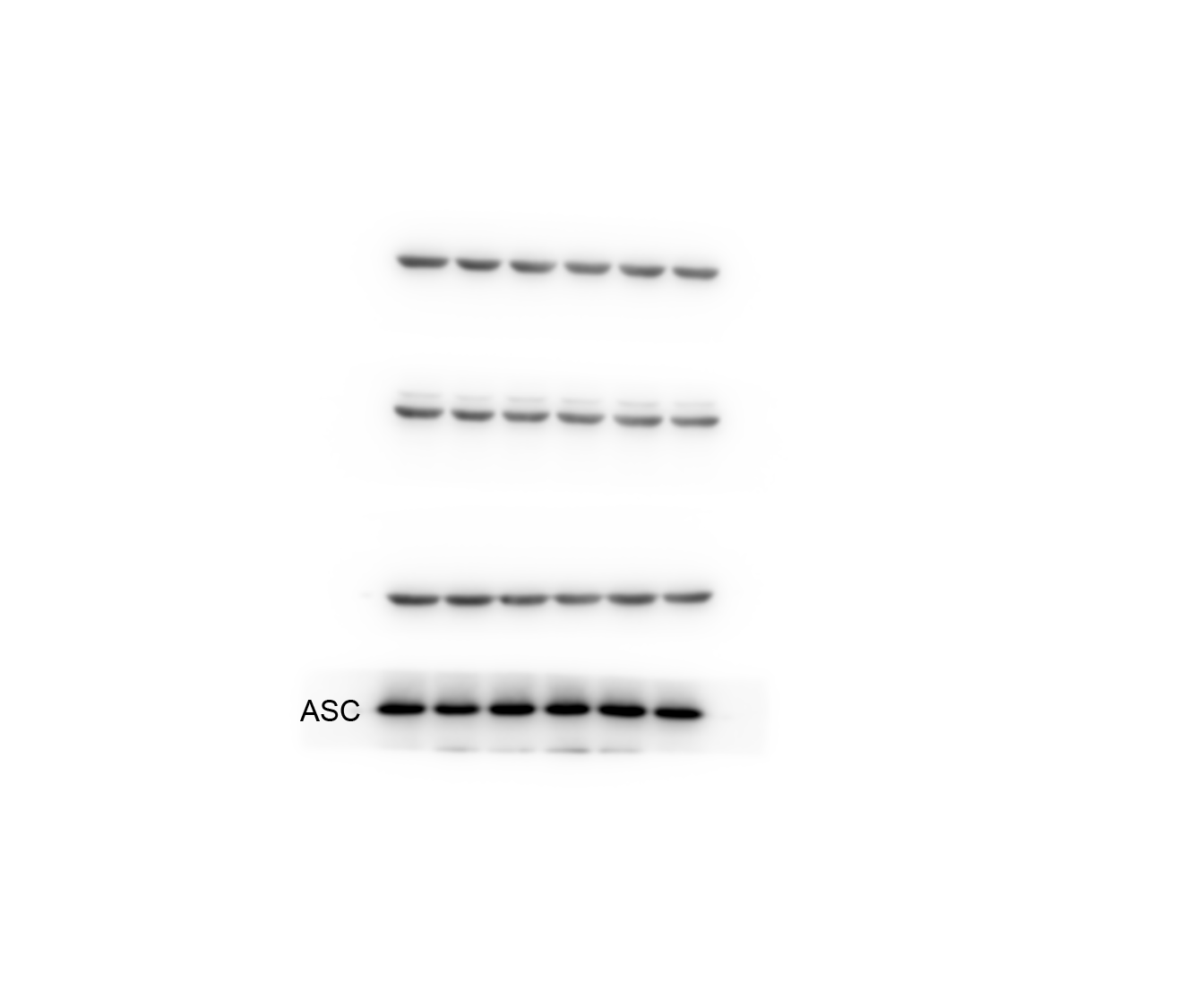

Supplement: Supplementary file 1 [file DataSheet1.ZIP › original WB figures/Figure8/Figure8B-ASC.tif]

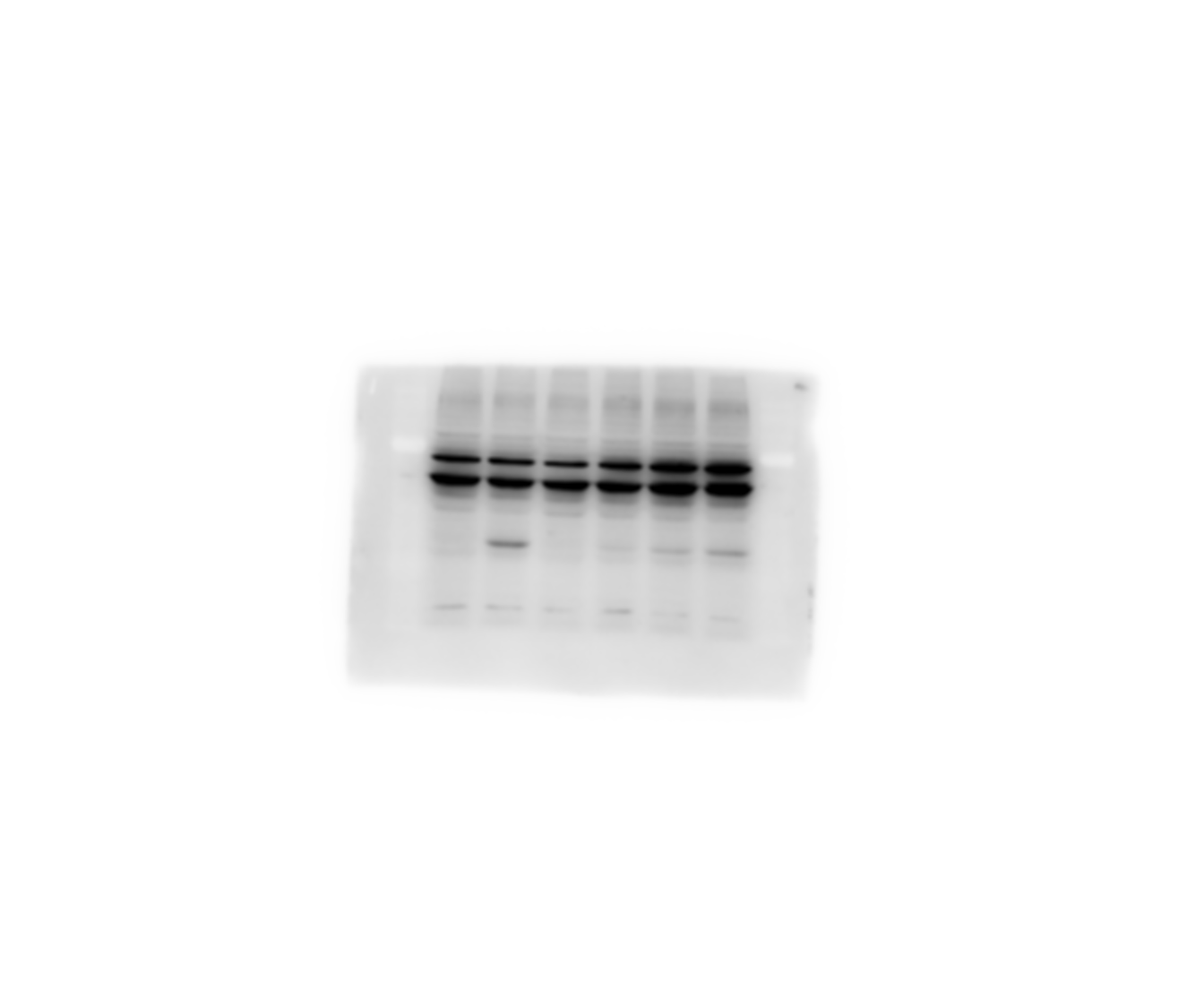

Supplement: Supplementary file 1 [file DataSheet1.ZIP › original WB figures/Figure8/Figure8B-GSDMD.tif]

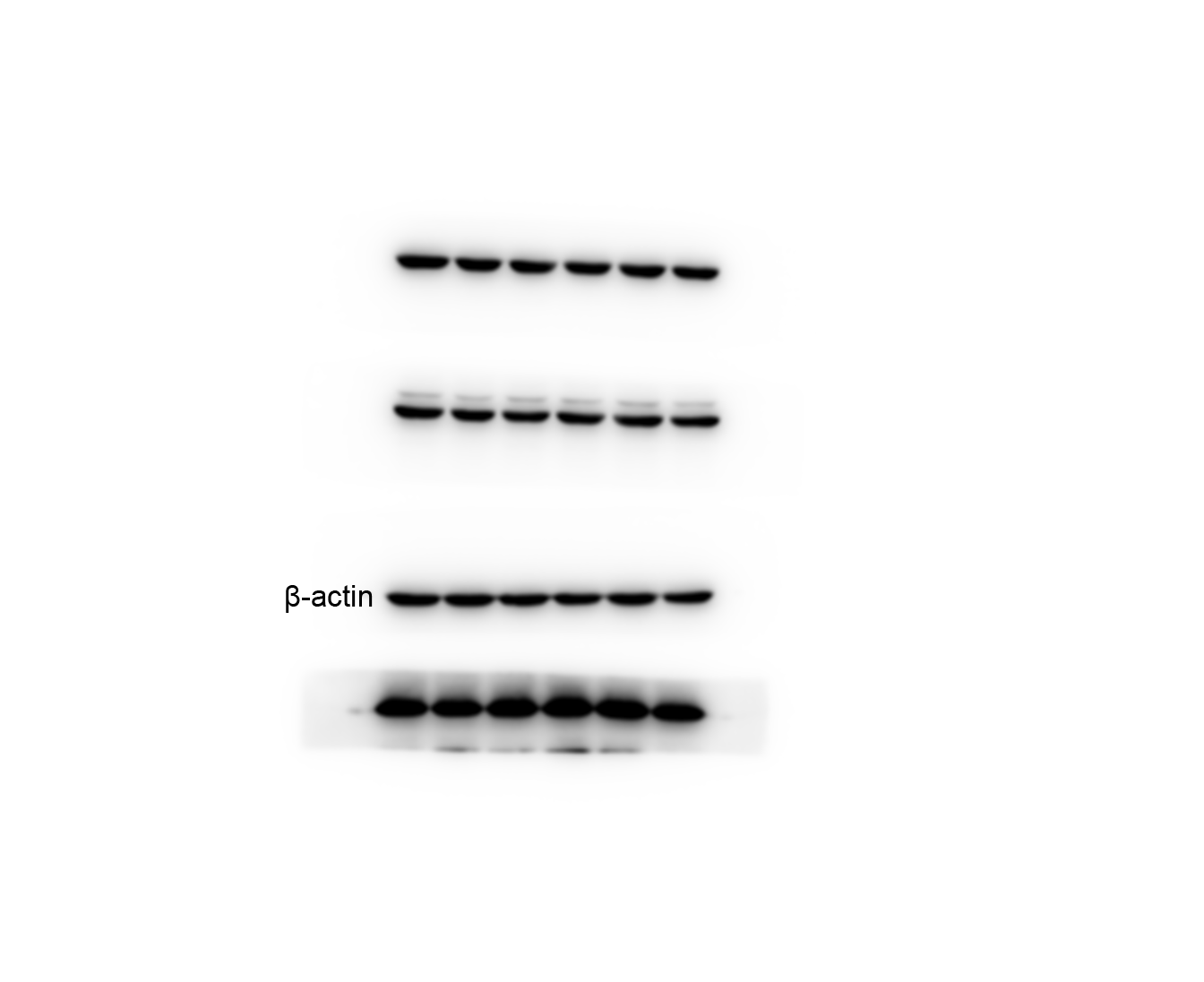

Supplement: Supplementary file 1 [file DataSheet1.ZIP › original WB figures/Figure8/Figure8B-β-actin.tif]

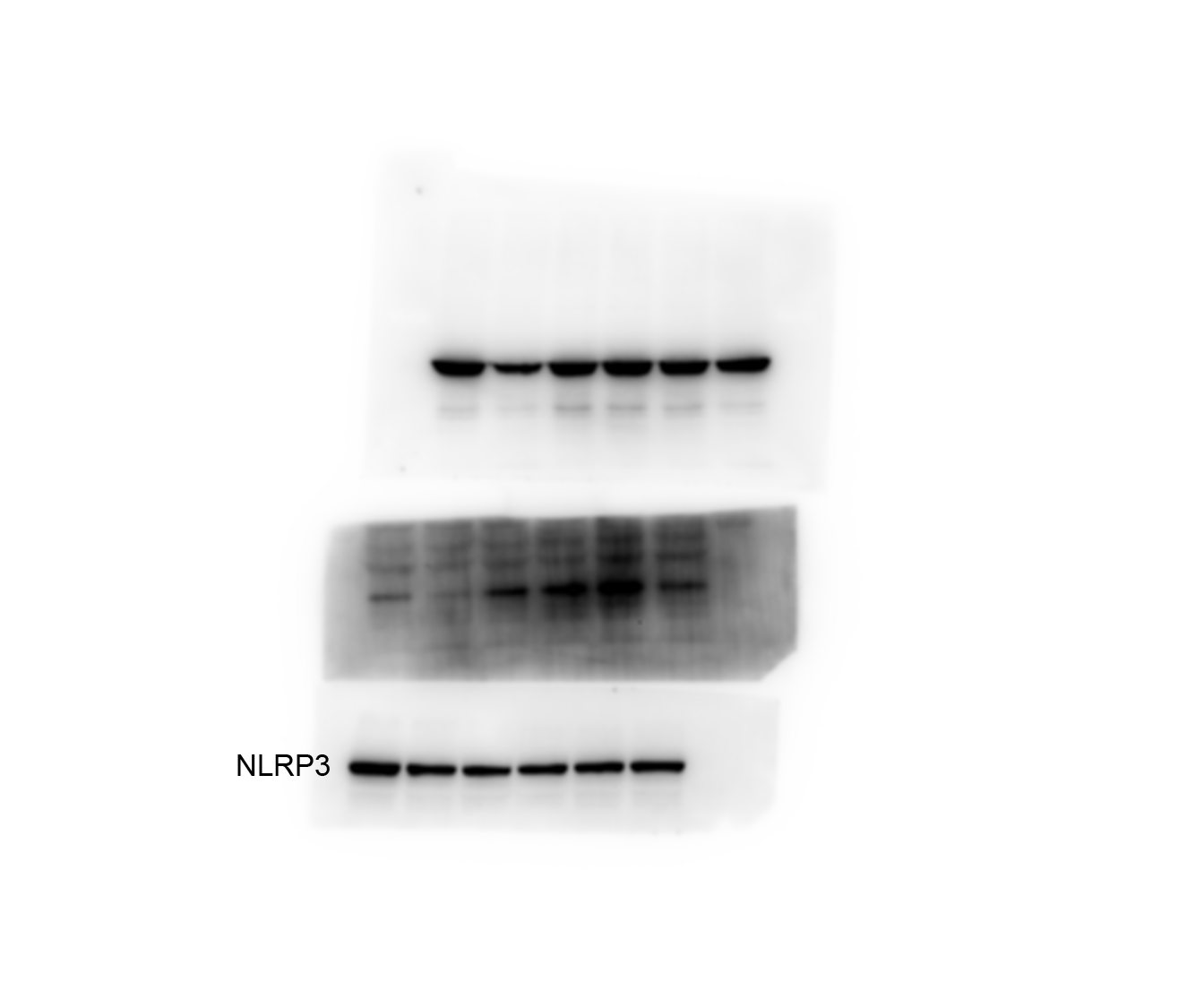

Supplement: Supplementary file 1 [file DataSheet1.ZIP › original WB figures/Figure8/Figure8C-NLRP3.tif]

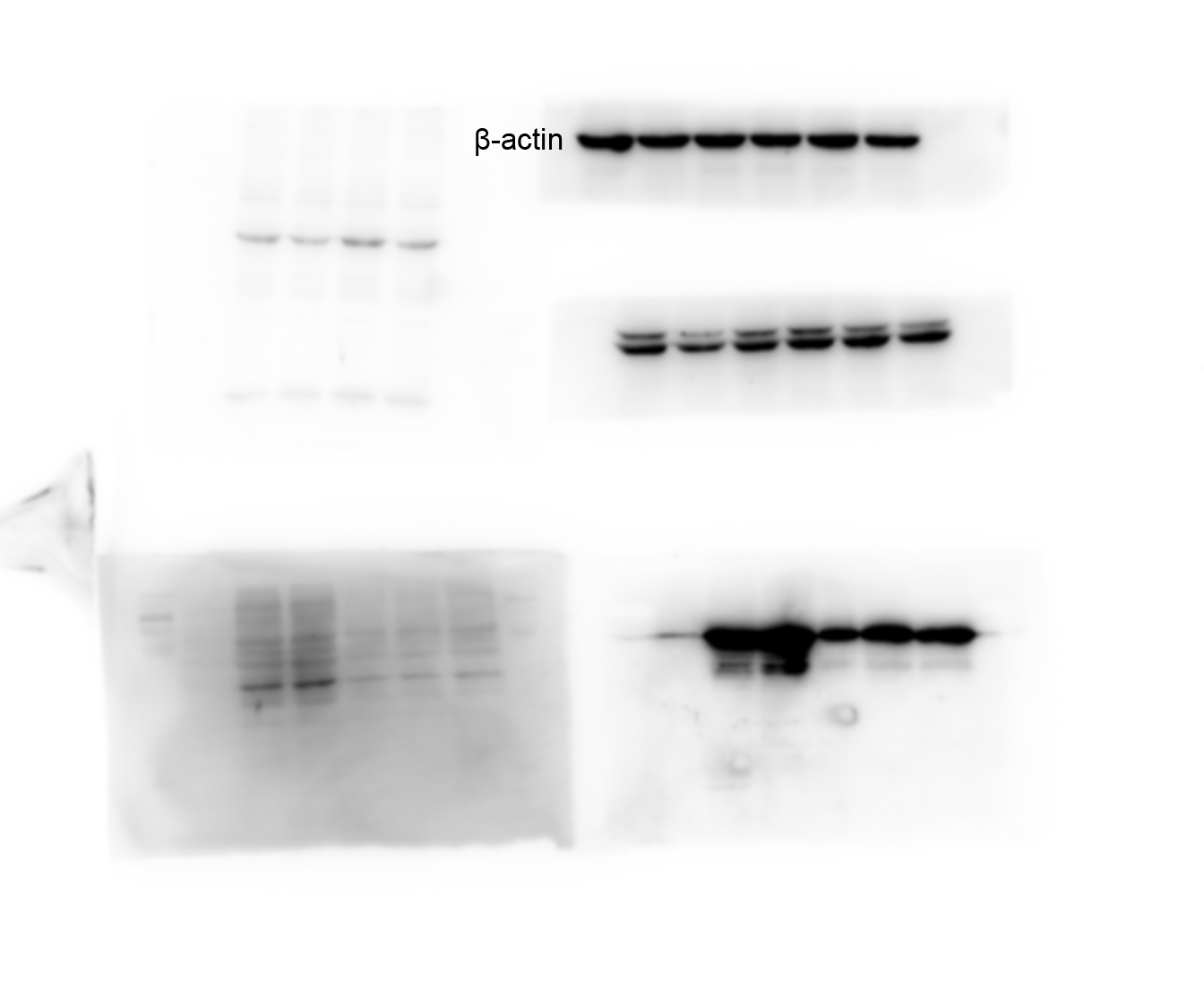

Supplement: Supplementary file 1 [file DataSheet1.ZIP › original WB figures/Figure8/Figure8C-β-actin.tif]

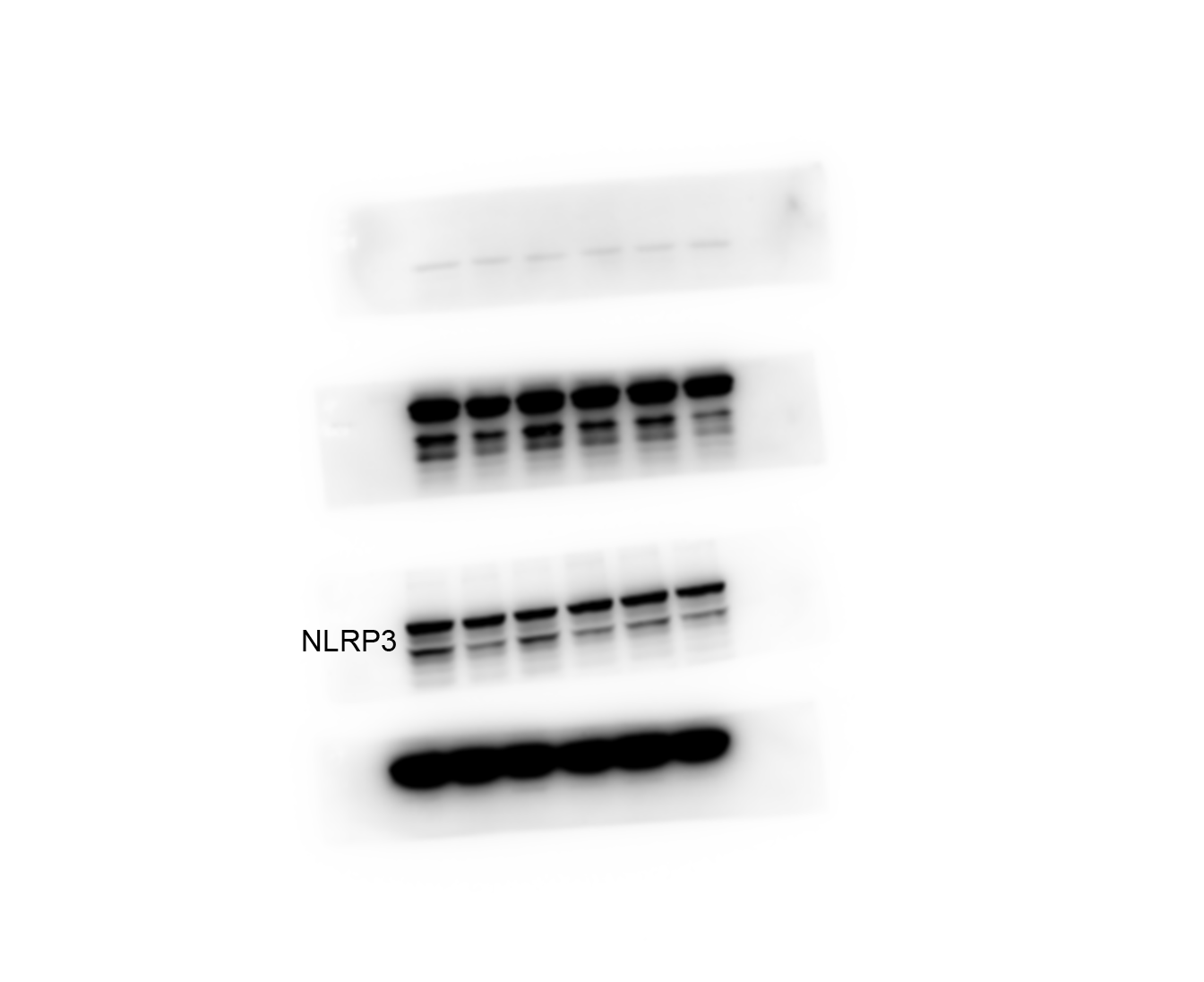

Supplement: Supplementary file 1 [file DataSheet1.ZIP › original WB figures/Figure8/Figure8D-NLRP3.tif]

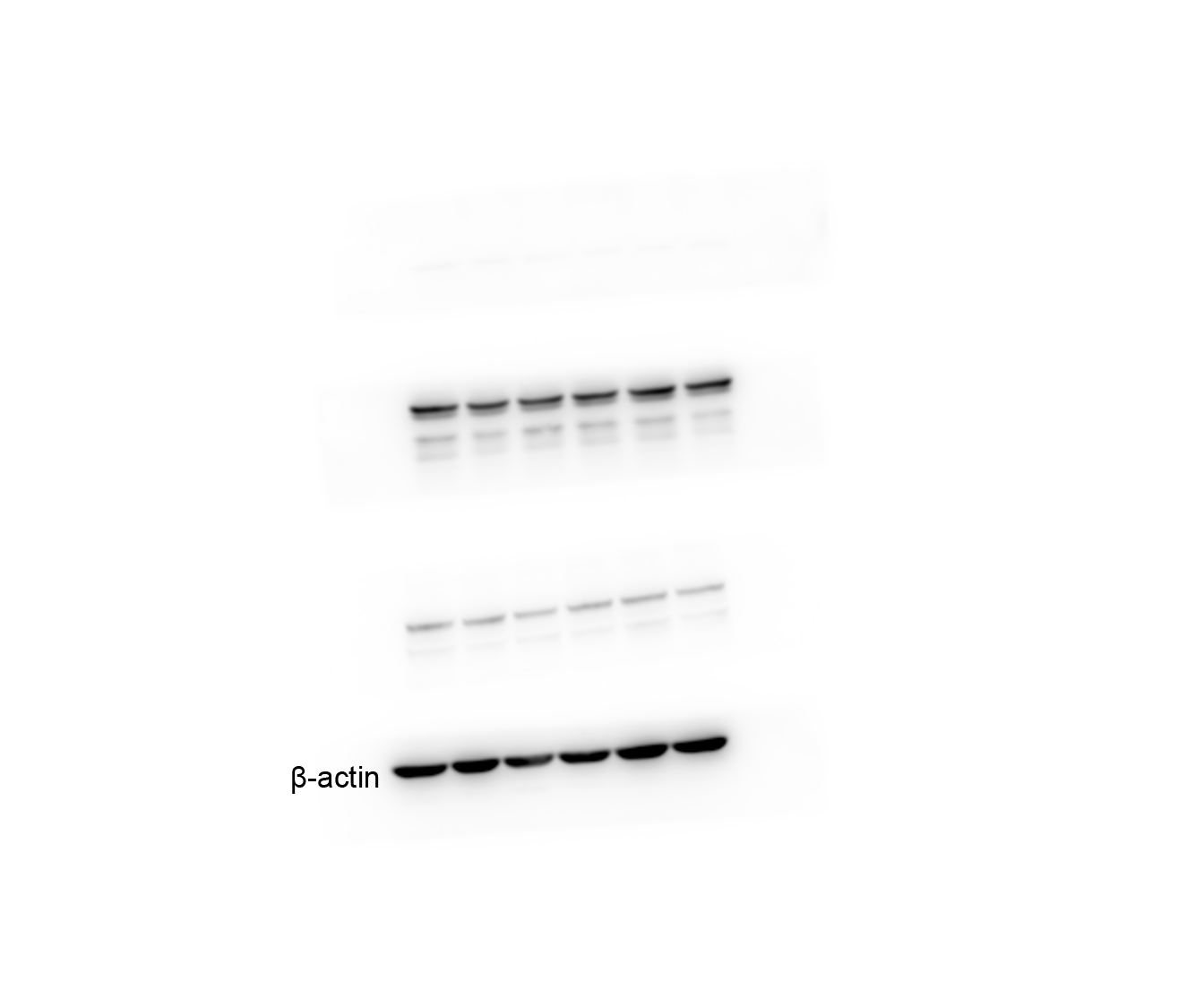

Supplement: Supplementary file 1 [file DataSheet1.ZIP › original WB figures/Figure8/Figure8D-β-actin.tif]

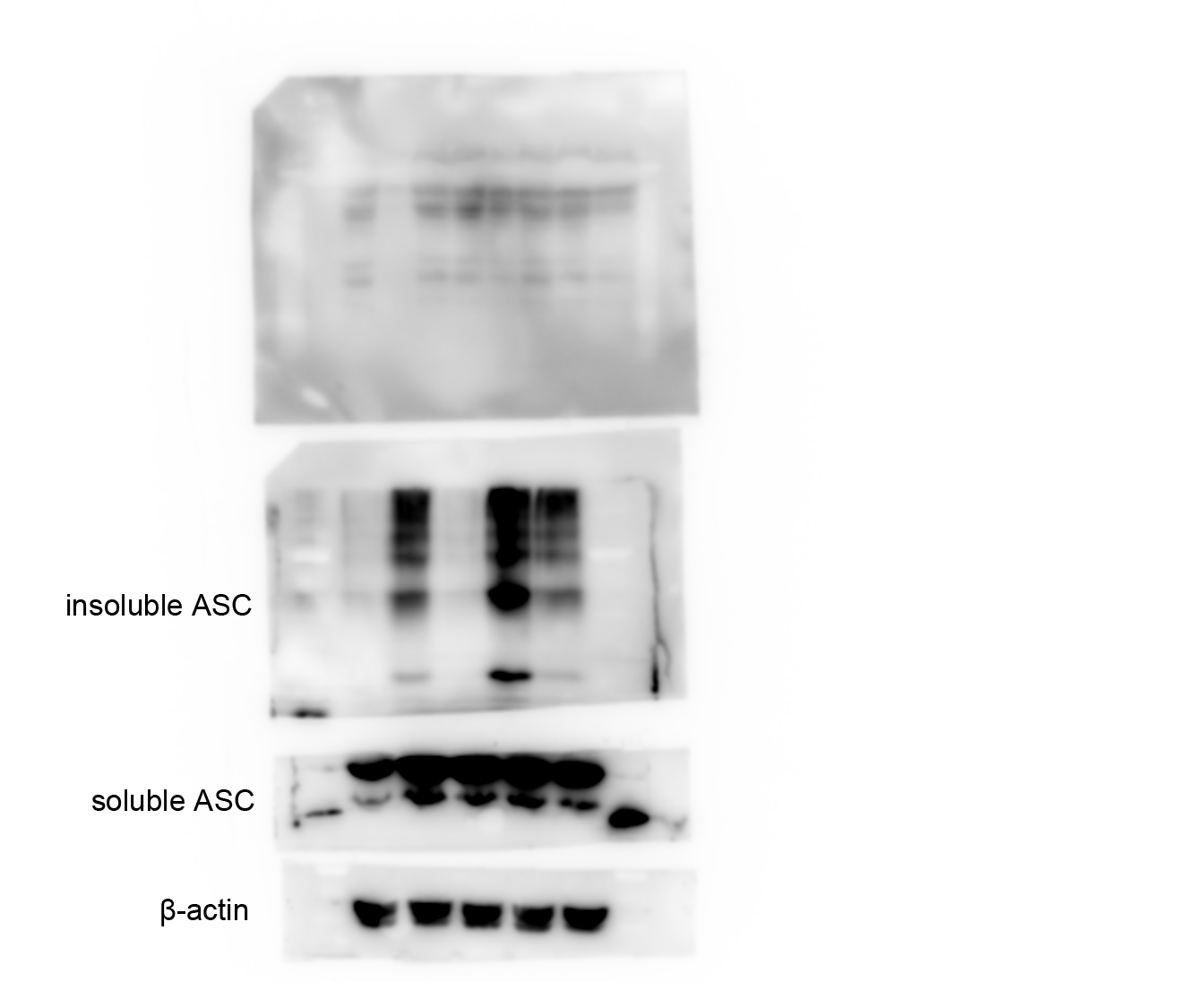

Supplement: Supplementary file 1 [file DataSheet1.ZIP › original WB figures/Figure8/Figure8G.tif]
