# Supplementary material for: The Essential Oil of Artemisia argyi H.Lév. and Vaniot Attenuates NLRP3 Inflammasome Activation in THP-1 Cells
Source: Front Pharmacol. 2021 Sep 16;12:712907. doi: 10.3389/fphar.2021.712907 (PMC8481632; doi:10.3389/fphar.2021.712907)
Supplement: Supplementary file 2 [file Image1.pdf]

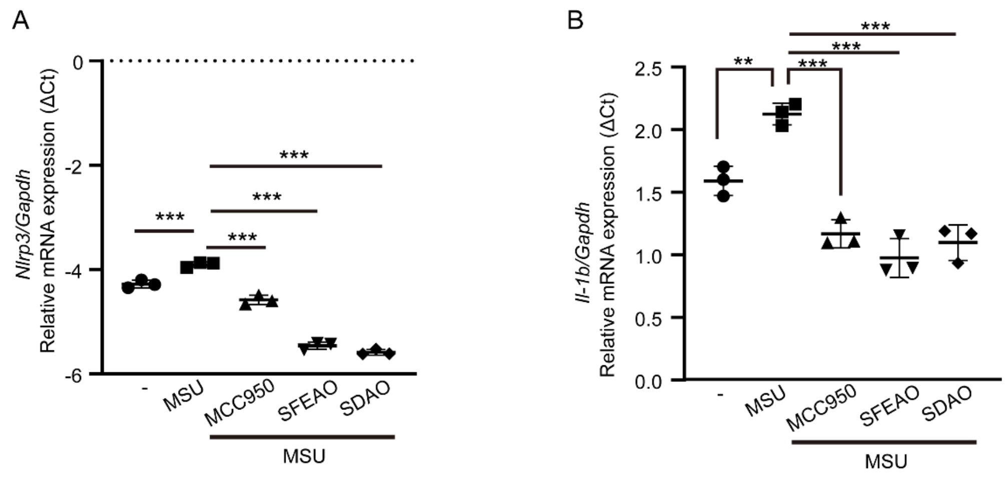

Supplementary Figure 1. EOAA inhibits the gene expression of NLRP3 and IL-1β after MSU treatment

PMA-primed THP-1 cells were treated with or without MCC950 (20 μM) or SDAO (45 μg/ml) or SFEAO (136 μg/ml) upon stimulation with MSU (500 μg/ml) for 6 h. The relative gene expression of NLRP3 (A) and IL-1β (B) in cell were measured by Quantitative Real-time PCR.GAPDH was acted as internal reference gene. ΔCt were from three independent experiments (A,B; mean and SD of n = 3).Statistics were analyzed using one-way ANOVA by Dunnett's t test, *P < 0.05, **P < 0.01, ***P < 0.001.
